# Collaborative care for the detection and management of depression among adults receiving antiretroviral therapy in South Africa: study protocol for the CobALT randomised controlled trial

**DOI:** 10.1186/s13063-018-2517-7

**Published:** 2018-03-22

**Authors:** Lara Fairall, Inge Petersen, Babalwa Zani, Naomi Folb, Daniella Georgeu-Pepper, One Selohilwe, Ruwayda Petrus, Ntokozo Mntambo, Arvin Bhana, Carl Lombard, Max Bachmann, Crick Lund, Jill Hanass-Hancock, Daniel Chisholm, Paul McCrone, Sergio Carmona, Thomas Gaziano, Naomi Levitt, Tasneem Kathree, Graham Thornicroft, the CobALT research team

**Affiliations:** 1 Knowledge Translation Unit, University of Cape Town Lung Institute, Cape Town, South Africa; 2 Centre for Rural Health, School of Nursing and Public Health, University of KwaZulu-Natal, Durban, South Africa; 3 Health Systems Research Unit, South African Medical Research Council, Durban, South Africa; 4 Biostatistics Unit, South African Medical Research Council, Cape Town, South Africa; 5 School of Public Health and Family Medicine, University of Cape Town, Cape Town, South Africa; 6 Department of Population Health and Primary Care, Norwich Medical School, University of East Anglia, Norwich, United Kingdom; 7 Alan J Flisher Centre for Public Mental Health, Department of Psychiatry and Mental Health, University of Cape Town, Cape Town, South Africa; 8 Centre for Global Mental Health, Institute of Psychiatry, Psychology and Neuroscience, King′s College London, London, United Kingdom; 9 HIV Prevention Research Unit, South African Medical Research Cuncil, Durban, South Africa; 10 School of Health Sciences, University of KwaZulu-Natal, Durban, South Africa; 11 World Health Organization Regional Office for Europe, Copenhagen, Denmark; 12 King’s Health Economics, Institute of Psychiatry, Psychology & Neuroscience, King’s College London, London, United Kingdom; 13 Department of Molecular Medicine and Haematology, University of the Witwatersrand, National Health Laboratory Service, Johannesburg, South Africa; 14 Cardiovascular Medicine, Brigham & Women’s Hospital, Boston, USA; 15 Department of Medicine, University of Cape Town, Cape Town, South Africa

**Keywords:** Antiretroviral therapy, Viral load, Depression, Mental health gap, Primary healthcare, Pragmatic trials, Low- and middle-income countries, Implementation science

## Abstract

**Background:**

The scale-up of antiretroviral treatment (ART) programmes has seen HIV/AIDS transition to a chronic condition characterised by high rates of comorbidity with tuberculosis, non-communicable diseases (NCDs) and mental health disorders. Depression is one such disorder that is associated with higher rates of non-adherence, progression to AIDS and greater mortality. Detection and treatment of comorbid depression is critical to achieve viral load suppression in more than 90% of those on ART and is in line with the recent 90-90-90 Joint United Nations Programme on HIV/AIDS (UNAIDS) targets. The CobALT trial aims to provide evidence on the effectiveness and cost-effectiveness of scalable interventions to reduce the treatment gap posed by the growing burden of depression among adults on lifelong ART.

**Methods:**

The study design is a pragmatic, parallel group, stratified, cluster randomised trial in 40 clinics across two rural districts of the North West Province of South Africa. The unit of randomisation is the clinic, with outcomes measured among 2000 patients on ART who screen positive for depression using the Patient Health Questionnaire (PHQ-9). Control group clinics are implementing the South African Department of Health’s Integrated Clinical Services Management model, which aims to reduce fragmentation of care in the context of rising multimorbidity, and which includes training in the Primary Care 101 (PC101) guide covering communicable diseases, NCDs, women’s health and mental disorders. In intervention clinics, we supplemented this with training specifically in the mental health components of PC101 and clinical communications skills training to support nurse-led chronic care. We strengthened the referral pathways through the introduction of a clinic-based behavioural health counsellor equipped to provide manualised depression counselling (eight sessions, individual or group), as well as adherence counselling sessions (one session, individual). The co-primary patient outcomes are a reduction in PHQ-9 scores of at least 50% from baseline and viral load suppression rates measured at 6 and 12 months, respectively.

**Discussion:**

The trial will provide real-world effectiveness of case detection and collaborative care for depression including facility-based counselling on the mental and physical outcomes for people on lifelong ART in resource-constrained settings.

**Trial registration:**

ClinicalTrials.gov (NCT02407691) registered on 19 March 2015; Pan African Clinical Trials Registry (201504001078347) registered on 19/03/2015; South African National Clinical Trials Register (SANCTR) (DOH-27-0515-5048) NHREC number 4048 issued on 21/04/2015.

**Electronic supplementary material:**

The online version of this article (10.1186/s13063-018-2517-7) contains supplementary material, which is available to authorized users.

## Background

In late 2014, the Joint United Nations Programme on HIV/AIDS set the ambitious 90-90-90 targets to renew efforts required to achieve an AIDS-free generation, stipulating 90% of people with HIV diagnosed, 90% of those who know their HIV-positive status on antiretroviral treatment (ART), and 90% of people on ART virally suppressed by 2020 [1]. Current debate regarding the targets has concerned the funding required and the shape of the interventions needed to be scaled in order to meet them [2]. These include focusing on key populations such as young women [3, 4], the introduction of universal test and treat programmes [5], and integration with related programmes including those for tuberculosis, maternal and child health, and sexually transmitted infections [6]. The intersection between HIV, non-communicable diseases (NCDs) and mental health is notably absent from this discourse, despite recent evidence demonstrating the scale and consequences of the collision of these respective epidemics. Increased incidence of hypertension, cardiovascular events and depression are well documented in people living with HIV and are two- to five-fold more common than in the general population [7–10]. This is against a backdrop where the 2013 Global Burden of Disease Study showed that major depressive disorder is emerging as a leading cause of Years Lived with Disability globally, secondary only to ischaemic heart disease, and among the top three causes of Years Lived with Disability in as many as 146 countries [11, 12].

Depression prevalence varies widely across HIV populations [13, 14]. Among those who are depressed, poor adherence is associated with acute life event threats and stresses, which predicts a decline in treatment adherence for HIV [14, 15]. These findings suggest that depression-prone HIV-infected individuals in the context of life stress are more likely to have lapses in adherence to treatment and may potentially compromise attainment of the 90-90-90 targets, especially as it pertains to adherence. This is particularly troubling where adherence to ART is paramount to achieving sustained virologic suppression, better clinical outcomes and avoiding the emergence of drug resistance [13].

Studies from high-income countries suggest that pharmacological and psychological treatments for depression and anxiety are equally effective in people living with HIV as they are in people not infected with HIV [10, 16]. However, studies from low- and middle-income countries (LMICs) are limited. In Uganda, Zimbabwe and Cameroon, pilot studies have shown that counselling [17, 18] or antidepressants [19] are effective in reducing depression scores. Additionally, counselling interventions have also been found to improve ART adherence [20], CD4 cell count and viral load suppression [19]. The studies from LMICs, however, were relatively small, involving around 40 to 320 participants and had a short duration of follow-up of only a few weeks to 4 months.

South Africa has the unfortunate distinction of having the highest number of people living with HIV/AIDS of any single country in the world. The country is home to 0.7% of the world’s population, yet 17% of all people infected with HIV live here [21]. After a much-publicised delay in rolling out ART, the South African National Health Department has now made considerable progress and runs the world’s largest treatment programme with roughly 3.4 million of the approximately 7 million people infected being on treatment [22]. The initial estimates of viral suppression rates were encouraging [23, 24], but have not been sustained as the programme is scaled up and people come to terms with the challenges of life-long treatment for sustained viral suppression, despite feeling clinically considerably better [25, 26]. The introduction, in 2009, of a fixed dose ART combination (FDC) has reduced pill burden substantively, comprising one tablet once a day, and approximately 90% of all people on ART in South Africa are receiving this. The South African programme has adopted a public health approach to monitoring, with viral loads recommended at 6 and 12 months after initiation and yearly thereafter. Over 4 million viral loads were measured in 2015; of the 75% of people in which a viral load test was performed in the previous 12 months, 78% were virally suppressed [27]. Heterogeneity is usual, with rates of viral load suppression varying between 69% and 82% across provinces. Interventions to strengthen adherence include spaced and fast lane appointment systems, adherence clubs and central chronic medicine dispensing and distribution. However, there has been limited attention paid to identifying and addressing underlying depression, which may be a major contributing factor to declining viral suppression rates [28].

This is consistent with the gap in mental healthcare provision in general. The South African Stress and Health study completed in 2003–2004, in which approximately 4500 adults were surveyed from communities in all nine provinces, found a lifetime prevalence of mental disorders of 30% and a 12 month prevalence of 17%. However, only a quarter of people with a 12-month diagnosis of mental disorder reported receiving any treatment from any source [29–31]. More recently, data from one of our previous trials undertaken in rural areas of the Western Cape showed that this gap had not narrowed; indeed, only 614 of 2466 patients who screened positive for depressive symptoms in primary care clinics reported any treatment (25%) and less than half of these reported treatment with antidepressants (294/2466, 12%) [32].

On the plus side, in 2013, the national Department of Health adopted a new national Mental Health Policy Framework, which, among other things, provides for the strengthening of mental health service delivery through primary care [33]. Mental health has also been included in an integrated approach to people living with chronic conditions, called the Integrated Clinical Services Management (ICSM) programme, and being introduced by the South African Department of Health [34]. The ICSM aims to promote a one-stop comprehensive service for people with multiple chronic and/or acute diseases, whether communicable or not, at the primary care level [35, 36]. One of its key elements is PC101, developed by the Knowledge Translation Unit at the University of Cape Town Lung Institute, which comprises a concise (101-page) guide of algorithms and checklists covering the main symptoms and chronic conditions that present in primary care [37]. Twelve onsite interactive training sessions in case scenarios are used to familiarise health workers with the content of the guide and to promote their use of it during consultations. Randomised controlled trials (RCTs) of PC101 and its precursors have shown positive impacts on a range of quality of care indicators, mainly in the area of communicable diseases, but no effect on depression case detection or treatment [38–40]. Process evaluation suggests that training alone is insufficient to overcome structural health system barriers presented by the limited availability of counselling and referral pathways, and restriction of antidepressant prescribing to doctors who are in short supply.

The Programme for Improving Mental Health Care (PRIME) [41] in South Africa has been working in one of the pilot districts for the ICSM for the past 4 years to develop and evaluate the feasibility of an integrated collaborative care model for priority mental disorders, including depression. The model includes strengthening of the mental health component of the PC101 training and referral pathways for management of common mental disorders [42]. These include the use of facility-based community health worker-level behavioural health counsellors to provide manualised counselling for chronic care patients with depression that has shown promise in a small pilot trial [43]; strengthened referral pathways for initiation of antidepressant medications by primary healthcare doctors; and onward referral to mental health specialists for more severe and treatment resistant depression. In addition, systems strengthening innovations to support these efforts have been introduced through the Emerald (emerging mental health systems in LMICs) programme [44], including clinical communication skills for nurse-led chronic care and a strengthened employee assistance programme for primary healthcare providers experiencing emotional problems and burn-out.

Development of the strengthened mental health intervention has been iterative, guided by the Medical Research Council framework for the development of complex interventions [45] and refined during two successive pilots, with the first in a single clinic and the second in a further three clinics. The resultant intervention is being scaled up to 10 clinics in the Dr Kenneth Kaunda District and a further 10 clinics in the adjacent Bojanala district during the period 2014–2017. The intervention targets depression comorbid with all common chronic conditions being managed in primary care. A pair of trials is evaluating the effects of this intervention on mental and physical outcomes among patients receiving ART and among those receiving hypertension treatment; these two conditions account for most chronic care attendances in South Africa [46]. Similarities and differences between the two trials are described in Table 1. The main distinction is the restriction of the hypertension trial to one of these districts, the eligibility criteria for patient participants and the choice of physical outcomes. This paper describes the trial focusing on those on ART. A separate paper describes the trial focusing on those on hypertension treatment.

**Table 1:** Comparison of the pair of trials evaluating the intervention developed during PRIME-SA

Characteristic	PRIME (Programme for Improving Mental Health CarE-SA) Trial	CobALT (Comorbid Affective Disorders, AIDS/HIV, and Long Term Health) Trial
Setting	Dr Kenneth Kaunda district, North West Province, South Africa	Dr Kenneth Kaunda and Bojanala districts, North West Province, South Africa
Clinic participants	20 primary care clinics	40 primary care clinics
Patient participants	Patients 18 years or older attending for hypertension treatment with a Patient Health Questionnaire score of 9 or more (*n* = 1000, 50 per clinic)	Patients 18 years or older attending for ART with a Patient Health Questionnaire score of 9 or more (*n* = 2000, 50 per clinic)
Control arm	The Integrated Services Delivery Model, which includes distribution and training in the PC101 guide	Same
Intervention arm	Three additional elements:1. Clinical communications skills training for nurse clinicians2. Supplementary training in the mental health components of PC1013. Clinic-based behavioural health counsellors equipped to provide morning talks on mental health to promote mental health literacy, manualised counselling for depression (8 sessions, individual or group) and adherence counselling (individual)	Same
Primary mental health outcome	Response at 6 months, defined as a 50% improvement from baseline in the Patient Health Questionnaire 9 score	Same
Primary clinical health outcome	Not applicable	Viral load suppression at 12 months
Duration of fieldwork	April 2015 to October 2016	April 2015 to December 2017
Controlled Trials Registration Number	NCT02425124	NCT02407691
Funding	UK Department for International Development	National Institutes of Mental Health, United States of America

### Objectives

The primary objective of the CobALT trial is to compare the effectiveness of a scalable intervention for collaborative care of depression by non-specialist mental health workers with usual services on the mental and physical outcomes in adults on lifelong ART with comorbid depression. The two primary hypotheses are that the intervention will (1) reduce depressive symptoms at 6 months and (2) improve viral load suppression status at 12 months. Secondary objectives are (1) to assess the effect of the intervention on the provision of integrated care for chronic illnesses in the intervention facilities from the perspective of service managers and providers as well as service users and (2) to evaluate the cost effectiveness of the intervention.

### Funding and registry

**Fig. 1:**
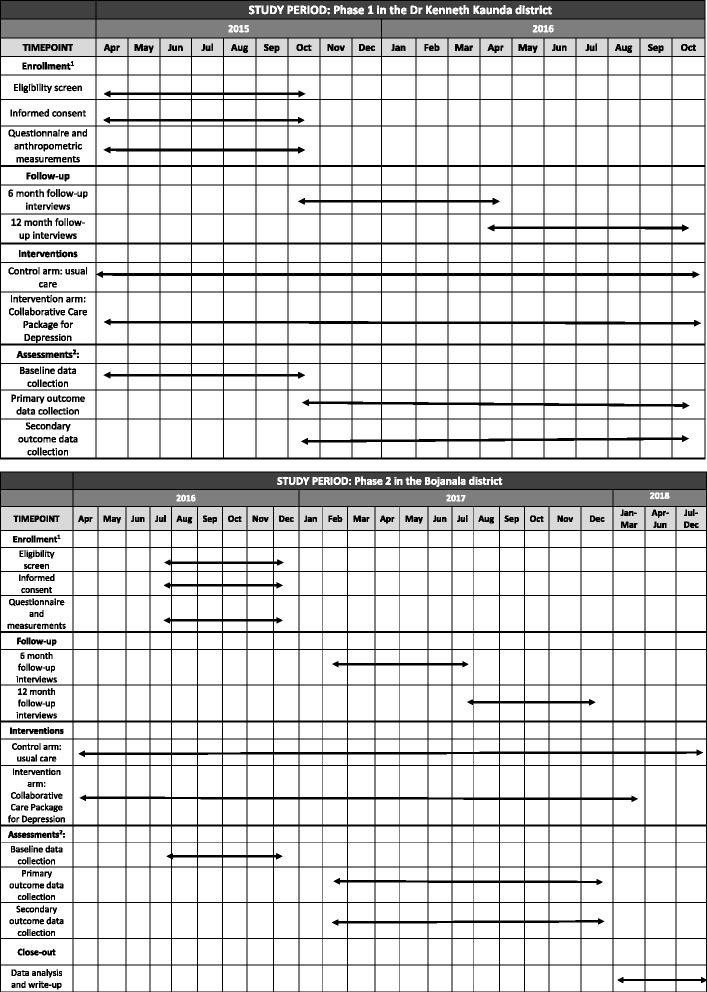
SPIRIT figure

This and other administrative information recommended by the SPIRIT checklist [47], including the structure, function and composition of all the trial committees, are summarised in Additional files 1 and 2 of the web-based supplementary files (see Additional file 1 for details, Additional file 2 for the SPIRIT checklist and Fig. 1 for the SPIRIT figure). The trial is supported through grant 5R01MH100470-03 from the National Institutes of Mental Health. The funder had no role in the design of the trial and will not have any role in the conduct of the trial, analyses, interpretation or decision to submit the results for publication.

## Methods

### Trial design

CobALT is a pragmatic, two-arm, parallel group, stratified and cluster randomised, superiority trial set in two health districts of the North West Province of South Africa. It is a type 2 hybrid design [48], evaluating an implementation strategy comprising clinical, educational and organisational elements (Table 3) in a coherent programme designed in collaboration with operational managers and health providers [42]. It combines characteristics of implementation (e.g. process evaluation and quality of care indicators) and clinical effectiveness research (e.g. primary health outcomes). We randomised primary healthcare clinics with a 1:1 allocation ratio, and will measure outcomes on individual patients. The trial is being implemented in two phases. The site for phase 1 is the Dr Kenneth Kaunda district (20 clinics, 1000 patients), where the intervention started in 10 clinics in April 2015 and patient recruitment commenced in April 2015. We are conducting phase 2 in the adjacent Bojanala district (20 clinics, 1000 patients), where the intervention started in 10 clinics in April 2016 and patient recruitment commenced in July 2016.

### Setting

The two study districts are typical of rural South Africa, where the public health sector is largely responsible for providing care to an impoverished rural population. The Dr Kenneth Kaunda district, named after the first president of Zambia, has a population of 700,000–800,000 people, and Bojanala has approximately 1,500,000 people, most of whom are Setswana speaking [49]. The main economic activities are mining and agriculture, and the unemployment rate is one of the highest in South Africa at 31.5%, above the national average of 30%. The 2013 National Antenatal Sentinel HIV Prevalence Survey showed that 28.2% of women attending antenatal services in the North West province are HIV positive compared with a national average of 29.7% [50]. ART is provided at 200 nurse-led primary care clinics across the two districts, but resources for mental healthcare are very limited. With the exclusion of a single psychiatric hospital servicing the North West Province in the Dr Kenneth Kaunda district, there are only 1.5 psychiatrists, 8 psychologists and 6 community service psychologists to render services to over 2 million people resident in the two districts. Unlike some provinces of South Africa, there are no dedicated specialised mental health nurses, although general primary care nurses receive some basic training in mental health. There is a regular supply of basic psychotropic medication to primary care, but no depression counselling interventions are routinely available.

### Participants

#### Clinics

**Fig. 2:**
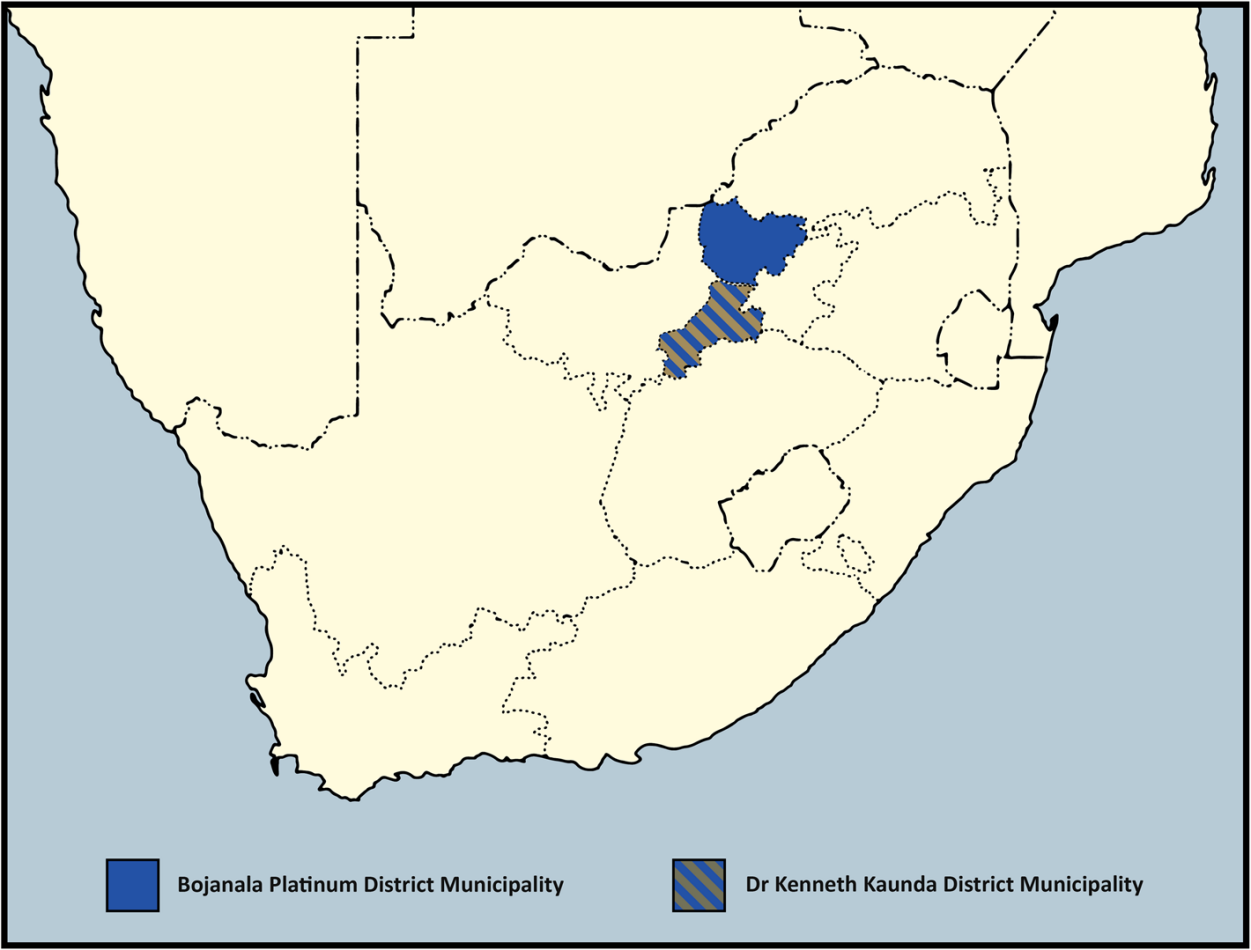
Location of the Dr Kenneth Kaunda and Bojanala districts in relation to South Africa

**Fig. 3:**
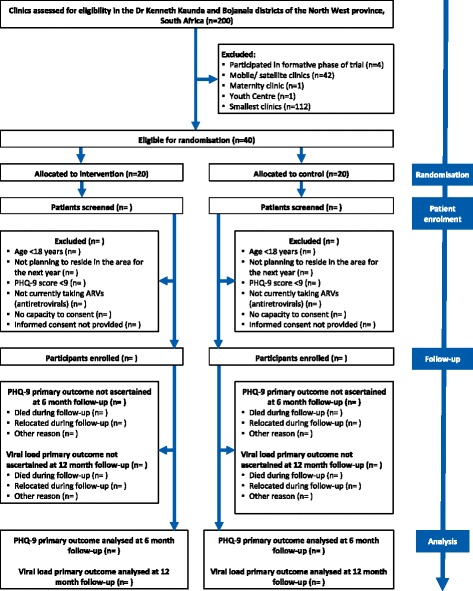
Flow of participants

We considered all public sector primary care clinics providing ART across the two districts for enrolment in the trial (Fig. 2). These clinics serve thousands of adult patients per year (median 13,693, interquartile range 5441 to 26,952), are nurse-led and supported by ward-based outreach teams of community health workers introduced as part of the re-engineering of primary care ahead of the introduction of National Health Insurance (NHI) in South Africa [51]. The Dr Kenneth Kaunda district is one of the 11 official districts participating in the piloting of NHI, although ward-based outreach teams have also been introduced in the Bojanala district. We excluded the four clinics which participated in the formative research to develop the intervention and pilot the data collection materials as part of the PRIME project in the Dr Kenneth Kaunda district. To ensure that we were able to reach individual patient recruitment targets in each clinic, we enrolled the 40 largest eligible clinics, 20 from the Dr Kenneth Kaunda district and 20 from the Bojanala district (Fig. 3).

### Patients

Inclusion criteria are age 18 years or older, receiving ART, depressive symptoms as indicated by a total score of nine or more on the Patient Health Questionnaire-9 (PHQ-9) [52], and valid consent. The PHQ-9 is a 9-item measure corresponding to the criteria upon which a diagnosis of depressive disorders is based in the Diagnostic and Statistical Manual of Mental Disorders (DSM-IV). Items are scored based on frequency of response ranging from 0 (‘not at all’) to 3 (‘nearly every day’) and summed assuming equal weighting to yield a follow-up score of between 0 and 27. A score of between 5 and 9 is associated with mild depression, between 10 and 14 moderate depression, between 15 and 19 moderately severe depression, and between 20 and 27 severe depression. It has been widely used for research and clinical purposes in primary care, including in LMICs [53–55].

As part of the preparatory work for the trial, we validated a Setswana version of the PHQ-9 among 676 chronic care patients in the Dr Kenneth Kaunda District, comparing the performance of a localised version of the PHQ-9 administered by fieldworkers to that of the Structured Clinical Interview for DSM-IV administered by a clinical psychologist. This showed that it is a valid tool for measuring depressive symptoms in the trial population [56]. However, we showed that a threshold of 9, as opposed to the more widely used threshold of 10, was more appropriate for detecting depression in this population and so applied it to the trial eligibility criteria. The PHQ-9 area under the receiver operator curve (AUROC) was 0.85 (95% CI 0.82–0.88). The PHQ-9 AUROC for the sub-samples of patients with HIV or with hypertension were comparable (0.85 and 0.86, respectively). The original and localised versions are shown in Table 2. During the localisation and lay panel testing process the category labels were changed from phrases ‘not at all’ to numbers (0 days) to facilitate response by local Setswana speakers.

**Table 2:** Patient Health Questionnaire-9: general and localised versions

Over the last 2 weeks, how often have you been bothered by any of the following problems?^a^	Not at all	Several days	More than half of the days	Nearly every day
Over the last 2 weeks, how often have you been bothered by any of the following problems?^b^	0 days	1–7 days	8–11 days	12–14 days
Little interest or pleasure in doing things	0	1	2	3
Feeling down, depressed or hopeless	0	1	2	3
Trouble falling or staying asleep, or sleeping too much	0	1	2	3
Feeling tired or having little energy	0	1	2	3
Poor appetite or overeating	0	1	2	3
Feeling bad about yourself, or that you are a failure or have let yourself or your family down	0	1	2	3
Trouble concentrating on things, such as reading the newspaper or watching television	0	1	2	3
Moving or speaking so slowly that other people could have noticed? Or the opposite, being so fidgety or restless that you have been moving around a lot more than usual	0	1	2	3
Thoughts that you would be better off dead or of hurting yourself in some way	0	1	2	3
Please could you confirm your answer for this question: Over the last 2 weeks, how often have you been bothered by thoughts that you would be better off dead or of hurting yourself in some way	0	1	2	3
	Total Score ___ = ___ + ___ + ___			
If you checked off any problems, how difficult have these problems made it for you to do your work, take care of things at home, or get along with other people?^a,b^	Not difficult at all	Somewhat difficult	Very difficult	Extremely difficult

^a^General version

^b^Localised version

In keeping with the pragmatic orientation of this trial [57, 58], we intentionally have few exclusion criteria. We only excluded individuals if they planned to move away from the vicinity of the clinic in the next year and would therefore be unable to participate in follow-up data collection, or were otherwise judged unable to provide informed consent because of acute medical illness or psychosis. We did not exclude participants with active suicidal ideation, but urgently referred them for clinical review in both intervention and control arms.

### Interventions

#### Control arm

The integrated chronic care programme implemented by the Department of Health (described above) forms the control comparison and was implemented in both intervention and control clinics prior to the introduction of the study intervention. At facility level, the main interventions were the reorganisation of chronic care services and training of clinic staff in PC101. Appointment systems and waiting rooms for communicable disease services (HIV, tuberculosis once the intensive phase of treatment has been completed) have been integrated with services catering for NCDs and mental health, resulting in a single chronic care service aimed at reducing fragmentation and promoting integrated care [35]. Nurses in the control facilities are able to refer patients identified as having depression to primary healthcare (PHC) doctors for the initiation of antidepressants (as they are not authorised to prescribe antidepressant medications in South Africa) as well as to mental health specialists, who are limited in number. Limited specialist psychological and psychiatric care is also available at the district hospital.

#### Intervention arm

**Fig. 4:**
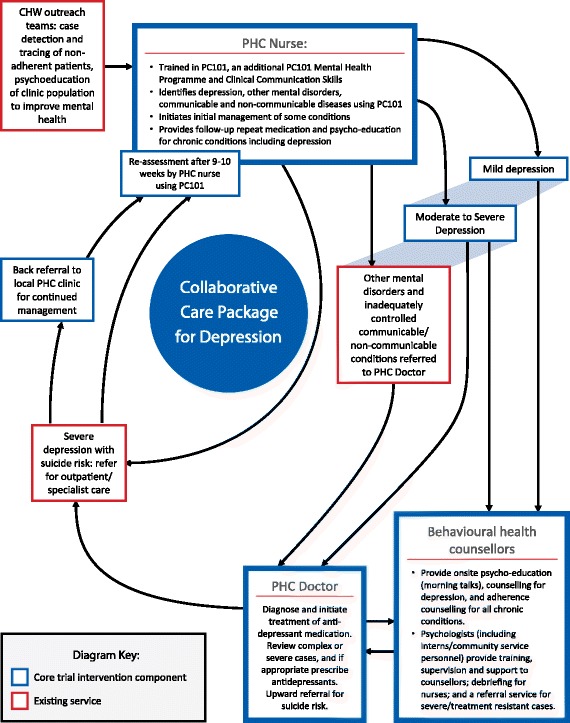
Collaborative care package for depression

In the intervention clinics, we supplemented the training of PHC nurse clinicians in the 12 PC101 sessions with clinical communication skills and four additional mental health sessions to strengthen identification and management of depression (see Additional file 3 of web-based supplementary material for examples of PC101 guides). We trained facility trainers in the intervention facilities to deliver this supplementary training material. We did not expose facility-based trainers in the control facilities to this supplementary training. In addition, we strengthened referral pathways for treatment and counselling (see collaborative care model depicted in Fig. 4). For treatment, we provided supplementary training of PHC doctors in mental healthcare. For counselling, we introduced lay behavioural health counsellors into the intervention clinics with structured supervision from district-based psychologists. The initial and long-term intention was to capacitate and diversify the role of existing facility-based lay HIV counsellors to include limited and structured counselling for service users with mild depressive symptoms, so as to ensure sustainability of the service. However, during the formative phase it became apparent that there were a number of scope of practice issues that needed to be addressed at a policy level before this was feasible [42]. So as not to delay the trial, the behavioural health counsellors were thus sponsored from study funds for the duration of the trial. In parallel, in order to optimize future sustainability of the intervention (should it be shown to be effective), the study investigators are engaged in a number of advocacy efforts with policy-makers to diversify the scope of practice of the existing lay HIV counsellors. A comparison of the training provided in the control and intervention clinics is contained in Table 3.

**Table 3:** Comparison of training provided to control and intervention clinics

Provider	Role	Training	Content of training	Method and timeframe
Control and intervention facilities				
PHC nurses	Identifies, provides brief interventions and refers	Basic onsite PC101 training	Case scenarios used for training in the identification and management of common chronic diseases, including communicable diseases, NCDs (including hypertension), women’s health and mental health. Mental health components draw on the WHO’s mhGAP guidelines [91] and adopt a syndromic approach to mental health symptoms (such as stress, insomnia, suicidal thinking) with diagnostic algorithms and treatment checklists for depression	(1) PC101 Master Trainers train Facility Trainers who train PHC nurses at the facilities(2) Twelve weekly sessions over 12 weeks at facilities (two of which are on mental disorders)(3) Training uses case scenario material of patients with chronic conditions, including comorbid conditions
Intervention facilities				
PHC nurses	Identifies, provides brief interventions and refers	Orientation and clinical communication skills training	(1) Overview of the system changes being made by the DoH in South Africa to accommodate the demands of integrated chronic care; their role as case managers within the collaborative care model for depression(2) Orientation to patient-centred care and clinical communication skills necessary to implement patient-centred care(3) Skills to manage patient emotions within the consultation; self-care including how to cope with their own emotions and burn-out(4) Motivational interviewing skills to promote patient self-management	Four 2-h interactive workshops at PHC facilities/regional training centre
		PC101 supplementary training in mental health	(1) Detection of depression and anxiety, psychoeducation and referral to counsellors and/or doctor for consideration of psychotropic medication in the case of moderate severe depression(2) Detection of risky alcohol use and brief intervention for harmful/hazardous drinking and for detoxification and referral to specialists rehabilitation programmes for dependency as per the mhGAP guidelines [91] (3) Assessment of suicide intent (4) Patient review after 8 weeks to assess response to treatment and onward referral for specialist care as indicated by the mhGAP evidence-based guidelines for LMICs [91], if necessary following a treatment-to-target approach as contained in the collaborative care model (Fig. 4); treatment to target involves tracking a patient’s symptom severity and adjusting or intensifying treatment should patients not show an improvement in symptoms following initial treatment.	(1) PC101 Master Trainers train Facility Trainers (2 day workshop) who train PHC nurses at the facilities(2) Three weekly sessions over 3 weeks at facilities, with an additional follow-up session 1 month later(3) Training uses case scenario material of chronic patients with comorbid mental disorders
PHC doctors	Diagnoses, initiates and monitors response to psychotropic medication	Orientation and training in mhGAP/PC101	(1) Orientation to the importance of treating comorbid depression (2) Training in mhGAP guidelines(3) Follow-up using case studies of patients	Three 1-day workshops spread over 6 months
Behavioural health counsellors	Provides evidence-based counselling	Counselling training	(1) Manualised counselling package comprising 8 sessions (delivered individually or in groups) (2) Session 1: Psycho-education session on depression; the last session is a closure session; Sessions 2–7 draw on problem solving and cognitive behavioural techniques, including behavioural activation to address the common triggers of depression and anxiety which, in this population, include poverty, interpersonal conflict, social isolation and avoidance, grief and loss, and stigma that emerged from qualitative interviews held with service users with during the formative phase of the PRIME project in South Africa in two provinces [92]; a prototype had been field tested in KwaZulu-Natal and positive results demonstrated in an individually randomised pilot trial [43]; adherence session provides information on the chronic condition(s) and chronic medication(s) the patients may have as well as helping patients with adherence difficulties (3) While developed to treat depression, the intervention has been found to promote improvements in global psychological functioning as well [43], thus having the potential for trans-diagnostic effects, in line with evidence that diagnosis-specific cognitive behavioural therapy has beneficial effects on untargeted comorbid emotional disorders [43]	One week of off-site training; one week of peer-to-peer mentoring; in vivo supervision by a psychologist of each session; weekly follow-up group supervisory sessions, augmented where possible by weekly individual supervision sessions
Specialists(Psychologist/psychiatrist)	Training, supervision of counsellors	Orientation to task sharing	Psychologists (including interns and community service psychologists) orientated to their roles	Once-off workshops

*DoH* Department of Health, *LMICs* low- and middle-income countries, *mhGAP* WHO Mental Health Gap Action Programme, *NCDs* non-communicable diseases, *PC101* Primary Care 101, *PHC* primary healthcare

### Patient-level outcomes

The co-primary patient mental and physical outcomes are (1) PHQ-9 response at 6 months, defined as at least a 50% improvement in PHQ-9 score compared with baseline; and (2) viral load suppression 12 months after patient enrolment, defined as a viral load value of < 1000 copies/mL in line with the third ‘90-90-90’ target [1].

There are multiple ways of managing the participant level analysis metric for the PHQ-9, which we are using both to define eligibility in this trial (score of 9 or more) and to assess response to treatments. We have selected as the primary outcome measure the response at 6 months, defined as a 50% improvement from baseline score. Secondary outcome measures related to PHQ-9 include response at 12 months; mean scores at 6 and 12 months; remission, defined as a score of 5 or less at 12 months; and category of severity (mild, moderate, severe) at 6 and 12 months. We record the treatment for depression, including initiation or intensification of antidepressant medication, referral to a counsellor for depression counselling, number of contacts with a counsellor, and referral to a mental health specialist (clinical psychologist, psychiatrist or secondary care services). The Perceived Stress Scale is included to measure the degree to which life situations are considered stressful [59].

Secondary HIV measures are viral suppression at 12 months defined as a viral load of < 400 copies/mL, virologic failure defined as two consecutive viral load values > 1000 copies/mL, and change in viral load values over time. Other ART-linked outcomes include programme retention, appropriate maintenance on enrolment ART regimen and ART regimen switched to second line, and the Internalised AIDS Related Stigma Scale [60], which we include to explore the relationships between stigma, retention in care and viral load suppression.

Viral loads will be measured using venous samples in participants at baseline and at the 12-month follow-up interview, and the samples submitted to the National Health Laboratory Service responsible for routine viral load monitoring, which current ART programme guidelines recommend should occur annually [61]. Thus, the majority of patients are expected to undergo both routine programme and research viral load monitoring during the course of the trial. Funds available for viral load monitoring as part of the trial are limited and therefore only patients with no documented programme results (in the past 6 months) will be asked to submit a baseline research viral load. Research-funded 12-month viral loads will be collected on all patients as this is the primary physical outcome.

We will also aim to collate all programme and research-viral loads into a single longitudinal record for every patient enrolled in the trial, through linkage with the viral load repository at the National Health Laboratory Service and application of algorithms for this linkage previously applied to province-wide ART datasets [27]. In South Africa, detectable viremia is most commonly caused by non-adherence, and only rarely due to resistant virus [62]. Thus, the proportion of participants with a suppressed viral load provides a reasonable estimate of ART adherence. In addition, we have included a Visual Analogue Scale for self-reported adherence in the last month as a secondary outcome measure at 12 months [63–65]. The integrated care nature of the intervention, with a focus on the detection and management of chronic diseases, and the surge in multi-morbidity in the general population, and ART populations in particular [66], are the reasons for monitoring risk factors for cardiovascular diseases (blood pressure, weight, smoking status) and the detection and treatment of other chronic diseases.

We will assess the provision of integrated care from a patient perspective using the Patient Assessment of Care for Chronic Conditions [67] at 12 months. Economic outcomes include healthcare utilisation, productivity and the World Health Organization’s Disability Assessment Scale [68]. We will monitor potential harms, including hospitalisation, death and suicide, the last two through follow-up at clinics, linkage with the national mortality register, and interviews with clinic staff and family members. Table 4 provides a full list of outcomes, time of measurement and source of data. In the section on ‘Adverse events and monitoring of possible harms’ and in Table 5, we describe in more detail outcomes reflecting possible harms and methods used for their detection.

**Table 4:** Schema of patient-level data collection

Outcome						
				Baseline	6 months	12 months
Outcome	Measurement	Source	Metric			
Primary measurements						
Depression symptoms	PHQ-9	Self-reported	50% reduction in PHQ-9 score	●^1^	●●^2^	●
Viral load suppression	Viral load value	Research- and programme-funded viral loads (latter through documentation during interviews and linkage to routine databases)	Viral load <1000 copies/ml	●	●	●●
Secondary mental health outcomes						
Depression symptoms	PHQ-9	Self- reported	50% reduction in PHQ-9 score	●	●	●●
Depression symptoms	PHQ-9	Self- reported	Remission defined as score of <5 on PHQ-9	●	●	●●
Depression symptoms	PHQ-9	Self- reported	Mean PHQ-9 scores at 6 and 12 months	●	●●	●●
Depression symptoms	PHQ-9	Self- reported	Category of depression: proportion with mild, moderate or severe depression	●	●●	●●
Antidepressant treatment		Self- reported	Proportion with antidepressant treatment initiated or intensified	●	●	●●
Counselling		Self- reported	Proportion receiving counselling by clinic-based counsellor	●	●	●●
Referral to specialist mental health worker/ service		Self- reported	Proportion referred	●	●	●●
Stress	Perceived Stress Scale	Self-reported	Difference in means	●		●●
Secondary HIV measurements						
Viral load suppression	Viral load value	Research- and programme-funded viral loads	Viral load <400 copies/ml	●	●	●●
Virologic failure	Viral load values	Research- and programme-funded viral loads	Two consecutive viral load values >1000 copies/ml	●	●	●●
Change in viral load values over time	Viral load values	Research- and programme-funded viral loads	At least half a log difference between viral load values, unless there are two viral loads >2000 copies/ml	●	●	●●
ART treatment	ART regimen	Self-report	Clinically appropriate switches to second line ART therapy	●		●●
ART adherence	Visual Analogue Scale	Self-report	Difference in means	●		●●
Stigma	Internalised AIDS-Related Stigma Scale	Self-report	Difference in means	●		●●
Integrated care outcomes						
Cardiovascular risk factors	blood pressure, weight, body mass index, waist circumference	Interviewer measured	Difference in means	●	●	●●
Diagnosis of other comorbid illnesses		Self-reported	Proportion diagnosed	●		●●
Disability	WHO Disability Assessment Schedule 2.0	Self- reported	Difference in means	●		●●
Quality of chronic illness care received	Patient Assessment of Care for Chronic Conditions (PACIC)	Self-reported	Mean PACIC score	●		●●
Retention in care		Self-reported; clinic records	Proportion in care	●		●●
Health economic outcomes						
Healthcare utilization		Self- reported; linkage with hospitalisation databases	Incidence rate ratio	●		●●
Productivity and economic outcomes		Self- reported		●		●●
Safety measurements						
Hospitalisation		Self- reported; linkage with hospitalisation databases	Proportion hospitalised	●	●	●●
All-cause mortality		Clinic, report, linkage with mortality register	Proportion died	●	●	●●
Suicide		Follow-up of cause of all known deaths with clinic and family interview	Proportion suicides	●	●	●●

^1^● data measured

^2^●● time when endpoint for outcome will be reported

**Table 5:** Defining, monitoring and reporting of harm

Type of harm	Source and method of identification	Action(s) to mitigate harm to specific participants	Reporting frequency and to whom
*Adverse Events*			
Positive response to ninth item of the PHQ-9: “Thoughts that you would be better off dead or of hurting yourself in some way”	Participant interviews (baseline, 6-month follow-up, 12-month follow-up)Flag within electronic questionnaire prompting interviewer to act	Repeat question to reduce telescoping-type reporting errorsIf ≥ 8 days in last 2 weeks, immediate referral to clinic staffIf between 1 and 7 days, then written educational material given	6 monthly report to DSMB6 monthly to IRB (with DSMB letter of recommendation)
PHQ-9 score of ≥ 20 at 12 months suggesting persistent severe depression	Participant interviews (12-month follow-up)Data report (monthly)	Summary forwarded to clinic together with recommendations for further treatment	6 monthly report to DSMB6 monthly to IRB (with DSMB letter of recommendation)
Blood pressure severely raised (≥ 180/110) placing participant at immediate risk of cardiovascular event	Participant interviews (baseline, 6-month follow-up, 12-month follow-up)Flag within electronic questionnaire prompting interviewer to act	Immediate referral to clinic staff for review	6 monthly report to DSMB6 monthly to IRB (with DSMB letter of recommendation)
Raised blood pressure at follow-up representing undiagnosed or uncontrolled hypertension	Participant interviews (baseline, 6-month follow-up, 12-month follow-up)Longitudinal patient record	Summary forwarded to clinic together with recommendations for further treatment	6 monthly report to DSMB6 monthly to IRB (with DSMB letter of recommendation)
Detectable viral load at follow-up representing possible adherence problems or treatment failure	Participant interviews (baseline, 6-month follow-up, 12-month follow-up)Research viral loadsRoutinely collected viral loadsLongitudinal patient record	Summary forwarded to clinic together with recommendations for further treatment	6 monthly report to DSMB6 monthly to IRB (with DSMB letter of recommendation)
*Serious Adverse Events*			
Significantly raised viral load (> 1000 copies/mL) during pregnancy placing fetus at risk of HIV transmission	Participant interviews (baseline, 6-month follow-up, 12-month follow-up)Research viral loadsRoutinely collected viral loadsData report (weekly)	Immediate notification of PI (LF) or delegate (NF) who will personally call clinic and follow-up with them until we can be sure woman is back in care and appropriately treated	Notification of IRB, DSMB and NIMH within 7 days of knowledge of confirmation
Hospitalisation	Participant interviews (baseline, 6-month follow-up, 12-month follow-up)Routinely collected hospitalisation dataData report (monthly)	No immediate action other than 6 monthly review by DSMB	6 monthly report to DSMB6 monthly to IRB (with DSMB letter of recommendation)
Death (excluding suicide)	Participant interviews (loss to follow-up form)National population registerData report (monthly)	No immediate action other than 6 monthly review by DSMB	6 monthly report to DSMB6 monthly to IRB (with DSMB letter of recommendation)
Death by suicide	Participant interviews (loss to follow-up form)National population register (provided we are able to access cause of death)Data report (weekly)	Immediate notification of PI (LF), who will follow-up with fieldwork staff to confirm suicide and establish date of suicide	Notification of IRB, DSMB and NIMH within 7 days of knowledge of confirmed suicide

*DSMB* Data and Safety Monitoring Board, *IRB* Institutional Review Board, *NIMH* National Institute of Mental Health, *PI* principal investigator

### Health system-level outcomes

We shall assess the provision of integrated care from a health system perspective using the Assessment of Chronic Illness Care, which covers six areas of system change to promote optimal chronic care (linkages to community resources, self-management support, decision support, delivery system design, clinical information systems and organisation of the health system) [69]. We will complete the Assessment of Chronic Illness Care on a sample of clinic staff from intervention and control clinics at 12 months. In addition, we shall conduct qualitative process evaluation interviews with one facility manager and one service provider from each of the intervention clinics at 12 months to understand their perspectives on how integrating mental health services for depression has affected overall HIV clinical care.

### Participant recruitment and timelines

We will conduct clinic recruitment in two phases. We deem this phased approach necessary because of the scope of the intervention support and fieldwork operation required for the trial, both of which place considerable demands on the trial co-ordinating centre operating with limited human resources and a limited budget. The trial will start in the Dr Kenneth Kaunda district because the intervention has been piloted in that district and because the comparison intervention, the Integrated Care Services Model, has been implemented in the district before Bojanala because of its inclusion as one of the NHI pilot districts. We will conduct clinic randomisation on two separate occasions to allow for changes in clinic operation (smaller clinics are closed or relocated on a regular basis in South Africa) and the volume of attendances. We will factor in an embedding period of 2 months between the start of the intervention and recruitment of patient participants. In determining the duration of the embedding period, we balanced the time required to ensure the intervention was functioning within the clinics with the likelihood that patient participants had not yet been exposed to the intervention by the time of enrolment. Figure 3 shows the recruitment of clinics and patient participants into the trial as well as follow-up evaluations.

### Sample size

We calculated the sample size for a superiority trial because we hypothesized that the intervention would lead to reduced depressive symptoms for patients on ART with comorbid depression, which would in turn positively influence their adherence to ART and care, and improve their HIV outcomes. We based our estimates of viral load suppression on data from our previous RCT on task-shifting HIV care in which 70% of ART patients had suppressed viral loads at follow-up with an intra-clinic correlation coefficient (ICC) of 0.046 [40]. We assumed that rates of viral load suppression will be slightly lower in a group of patients suffering from depression (65%) as it is a known risk factor for poor adherence [18, 70]. A sample size of 2000 participants from 40 clinics (50 patients per clinic, 1000 per group) provides 80% power to detect a 10% difference in 1-year viral load suppression at the 5% significance level assuming an ICC of 0.04 and a 20% loss to follow-up based on a previous trial in this setting [38]. To determine the appropriate parameters for the PHQ-9 sample size calculation we searched PubMed for all RCTs which had used the PHQ-9 as an outcome. Of the 126 trials screened, we identified nine as relevant to the design of our trial [71–79], including four cluster trials and five individually randomised studies. The trials used a mix of metrics, including difference in means, response and remission rates, measured at a range of time points. We disregarded several of the trials because their baseline scores were far higher than those we had observed in our population of interest during the validation of the Setswana localisation of the PHQ-9. We instead based our sample size estimates on three cluster trials completed in the Netherlands [71], Italy [77] and the UK [79], where PHQ-9 response rates at 6 months were reported and where baseline scores were similar to what we anticipated. Only two of the five cluster trials reported an ICC [71, 79] and, in one of these, it was uninterpretable because of very small cluster sizes [71]. We then completed a series of calculations to determine power to detect a difference of between 10% and 12% in response rates at 6 months, with the number of clusters and cluster size fixed by the viral load calculation at 40 and 50, respectively (Table 6). We allowed for the proportion of those who responded in the control group to vary between 30% and 35%, for the ICC to vary between 0.02 and 0.04 (we assumed lower ICCs were likely given that the PHQ-9 is a health outcome) and for a loss to follow-up rate of 20%. This showed that a sample of 50 patients in 40 clusters (1000 per group) yielded a power of between 73% and 96% to detect a difference of between 10% and 12% in response rates at 6 months.

**Table 6:** Power calculations for primary PHQ-9 outcome (response rate at 6 months defined as 50% improvement from baseline)

No. of clusters per group	Cluster size	Proportion responded in control group	Proportion responded in intervention group	Alpha	ICC	Power (No dropout)	Power (20% dropout)
20	50	0.30	0.40	0.05	0.04	76	73
20	50	0.30	0.40	0.05	0.02	90	87
20	50	0.30	0.42	0.05	0.04	89	87
20	50	0.30	0.42	0.05	0.02	97	96
20	50	0.35	0.45	0.05	0.02	89	87
20	50	0.35	0.47	0.05	0.02	97	95

### Recruitment and data collection

We conducted clinic recruitment in two phases. We obtained an updated list of clinics in each district with recent headcount data. We scrutinized the GPS co-ordinates of the clinics to identify any clinics that might be located next to each other and thus be at risk of contamination. We then forwarded a cleaned-up list of eligible clinics to the statistician, who selected the largest facilities and completed randomisation within sub-district strata. Because clinics in South Africa sometimes close or are amalgamated with others, we repeated the process for the Bojanala clinics a few months before starting work in that district. We split clinic recruitment evenly between the two districts with 20 clinics from each.

Patient recruitment occurred independently of clinical care or participation in the group psychosocial intervention for depression. We adopted a pragmatic approach, enrolling all patients who met the eligibility criteria regardless of exposure to the intervention, recognising that the detection of mental disorders and linkages to care are pressing challenges for integration of mental health services into primary care and that failure to account for this in the design of the trial would limit the generalisability of its findings to real-world primary care settings. Patient participant recruitment started approximately 2 months after the training of nurses and counsellors in the intervention clinics. Trained fieldworkers invited, screened and enrolled patient participants in the trial using a three-stage process as outlined in Additional file 4. First, trained fieldwork recruiters explained the study to patients in the waiting room, inviting those attending for chronic care services to come forward for more information if they were interested. Depression, HIV or ART were not be mentioned during this general information sharing so as not to single out those who wished to participate. The recruiter completed a brief verbal screen of patients who came forward to establish whether or not they were on ART and whether they planned to stay in the study area for the duration of the following year; this was done on a one-to-one basis. Eligible patients were then walked across to a fieldwork interviewer based in a private section of the clinic where the emotional content of the PHQ-9 instrument was explained and opt out consent sought before proceeding with the full screen. We then sought formal consent from patients who screened positive using the PHQ-9. This covered permission to be interviewed at enrolment, at the two subsequent time points, 6 and 12 months later, determination of the participant’s viral load at enrolment and 12 months later, permission to review their clinical folders and hospital records to assess quality of care provided and healthcare utilisation, and to send reminders and confirmation of follow-up interviews by text message and phone calls. We requested participants to supply their national identity numbers to permit linkage with the national population register, which records more than 90% of all deaths in South Africa. We only collected patient identifiers once written informed consent was provided (Additional file 5). The recruitment process for the PRIME and CobALT trials was conducted as a combined process in the Dr Kenneth Kaunda district and patient recruitment continued until the target of 50 patients per clinic was met for both trials. We invited patients who were on both ART and antihypertensive medications, and who qualified for enrolment in the trial, to participate in both studies and complete two sets of consent forms. Fieldwork interviewers conduct interviews in one of the three local languages (Setswana, English, Afrikaans), chosen by the participant (see Additional file 6 for the full questionnaire). Fieldwork interviewers collect data using handheld electronic devices, which we have previously used in similar field work data collection exercises in South Africa [80]. We ask participants to return to the clinic for follow-up interviews. We send text reminders in advance of follow-up visits, and contact participants by telephone to confirm their appointments. At follow-up, participants undergo an interview to determine the severity of their depressive symptoms, the nature of the care they received at the clinic, including care for depression and HIV, and other healthcare utilisation and economic outcomes. Follow-up viral loads will be drawn from all patients at 12 months.

### Randomisation, allocation concealment and blinding

We randomised clinics and their patients to one of two parallel groups, with an allocation ratio of 1:1. Within each district, we considered the distribution of clinics within sub-districts to ensure that we did not introduce potential confounding through geographically determined management of clinics. Within the Dr Kenneth Kaunda district, 12 clinics fall within the Matlosana sub-district in the area, including and surrounding the major town of Klerksdorp, and have been randomised as one stratum. The remaining eight clinics are situated in more rural areas and randomised together. Within the Bojanala district, 10 clinics are in the central district of Rustenburg in and around the town of the same name and are randomised in a 1:1 allocation. The remaining 10 clinics are distributed over a far wider area than in Dr Kenneth Kaunda district and spread across the three sub-districts of Moretele, Kgetleng River and Madibeng. We combined two of the smaller sub-districts (Moretele and Kgetleng River) into one, so that we have two strata each with five clinics available for randomisation. We randomly selected one of these strata to have three intervention clinics to two control clinics, and vice versa in the other stratum, so that we have 10 intervention clinics and 10 control clinics in the Bojanala district. The trial statistician carried out randomisation using n-Query advisor prior to the intervention or screening of participants for recruitment. Blinding of participants and clinicians is not possible because it will be clear which clinics will be implementing what interventions. The trial statistician who carried out randomisation did not know the characteristics of the clinics being randomised, and the primary statistical analysis will also be blinded to allocation status.

### Data management

**Fig. 5:**
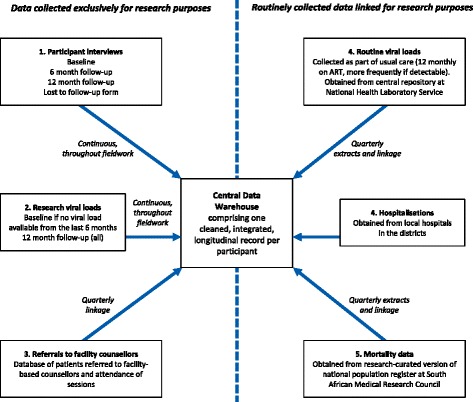
Integration of data sources into a longitudinal patient record

Interview data is uploaded as soon as questionnaires are completed, internet connectivity permitting, and stored in a secure SQL server at the Trial Co-ordinating Centre at the University of Cape Town Lung Institute. We integrate data from interviews with those from other sources to create a longitudinal record for every patient participant (Fig. 5). These other sources include blood results for the viral load samples drawn as part of the research process as well as routinely monitored viral loads which are collated in a central national repository. We search hospitalisation records quarterly and integrate linkages into the research dataset. We maintain a separate database of patients referred to facility-based counsellors and link their attendance of sessions with the trial patient database. The research version of the national population register, curated by the South African Medical Research Council, will be linked once at the end of the study to identify any deaths which had not already been identified through follow-up of patients who did not return for their 6- and 12-month interviews.

Quality control measures include supervision of fieldworkers, electronic alert messages for fieldworkers if unusually high or low values are entered into the electronic questionnaire, automated skips, and monitoring of the data to identify unusual values or trends. The handheld devices used for data collection are password protected and interview data is uploaded at the end of each interview without being stored on the device. Only a small number of data managers have access to personal identifiers. Anonymised data (without names or national identity numbers) will only be provided to selected members of the research team. Members of the research team, including all fieldworkers and fieldwork supervisors, are required to sign a confidentiality agreement to ensure the protection of confidential data.

### Statistical methods

We will estimate the effects of the intervention by comparing the outcomes of intervention and control group patients using multiple mixed effects regression models, with clinics as a random effect to account for the intra-cluster correlation of outcomes. We will include the stratum as a covariate in the regression models, and will use Stata statistical software for the analysis. All clinics and patients will be analysed in the treatment group to which they were randomly assigned. We will ascertain the completeness of follow-up by group to evaluate the integrity of the planned analysis. We will use binomial regression to estimate differences in proportions of patients with suppressed viral loads and who have responded in terms of their depressive symptoms, measured using the PHQ-9. We will analyse secondary outcomes as follows: binomial regression for binary outcomes, linear regression for changes in depression scores, blood pressure, weight, and waist circumference by comparing values at follow-up adjusted for baseline values, and Poisson regression for count outcomes such as clinic visits and inpatient days, accounting for individuals’ duration of follow-up.

Planned secondary analyses include analysis of primary outcomes stratified and adjusted for treatment severity, treatment status and comorbidities at baseline. We will also explore whether there is a differential effect among those patients who reported receiving the counselling intervention.

The economic evaluation will include descriptive statistics describing the health system and societal costs of living with HIV and depression, a cost effectiveness analysis, and modelling of the intervention beyond the timeframe of the study.

### Adverse events and monitoring of possible harms

These are enumerated and defined time frames for monitoring and reporting as specified in Table 5. Most guidance would recognise hospitalisation, or prolongation of hospitalisation, and death as serious adverse events, requiring prompt reporting to regulatory bodies. However, both of these events are expected to occur frequently among adults on ART. Based on follow-up from our previous trial in a similar ART population, we expect a mortality rate as high as 5% during the 12-month follow-up period, and a hospitalisation rate of approximately 10% [40]. We expect much of this mortality and morbidity to be due to the underlying HIV infection, advanced enough to warrant treatment with ART prior to the introduction of universal treatment. Systematic monitoring of hospitalisations and deaths is planned, but will be episodic, occurring at the 6- and 12-month follow-up participant interviews, and with quarterly linkage of hospitalisation and once-off linkage with the national population register data. Given that these events, while serious, are common in this population, are likely to be related to the underlying HIV disease progression, and that monitoring is episodic, we have proposed that the frequency of these events be reviewed 6 monthly by the Data and Safety Monitoring Board (DSMB), and then reported to the relevant Institutional Review Boards (IRBs) (University of Cape Town, University of Kwazulu-Natal and King’s College London), with the DSMB Letter of Recommendation. Because suicide may well be related to a mental health intervention, and is likely to be rare, we report any such events within 7 days of the knowledge of the event in accordance with the recommended Standard Operating Procedures of the University of Cape Town’s IRB. All deaths will be followed up with clinic staff and family members to determine whether or not they were the result of suicide.

A further serious adverse event that is unlikely to be related to the intervention, but which may occur as a result of participation in the data collection component of the trial, is a markedly elevated viral load in the context of pregnancy, placing the foetus at risk of transmission. For this reason, it will be managed as a serious adverse event and reported to the IRB and DSMB within 7 days. We follow-up all viral loads in excess of 1000 copies/mL to see whether or not they appear to be reported during pregnancy and we urgently pursue all missing viral loads in women who report themselves to be pregnant. Once we confirm a high viral load in a pregnant woman, we immediately notify the clinic staff and advise them to recall the women urgently for clinical care and intervention to limit transmission of HIV to the baby.

We report non-serious adverse events to the DSMB 6 monthly. We ask the participants who respond positively to the ninth item of the PHQ-9 “Thoughts that you would be better off dead or of hurting yourself in some way” to confirm their answer and, if they indicate having such thoughts between 1 and 7 days in the last 2 weeks, we provide them with written educational material on suicide prevention. If they confirm having such thoughts for 8 days or more in the past 2 weeks, we immediately refer them to clinic staff for review. This protocol applied to the pre-screening process even for patients who responded positively to the ninth item of the PHQ-9 but were otherwise not eligible for enrolment in the trial. Likewise, we immediately refer participants with a blood pressure of ≥ 180/110 to clinic staff for review of their blood pressure. Once data collection is complete, we will present a summary of adverse events to clinics together with recommendations for further treatment. These adverse events are a PHQ-9 score of ≥ 20, suggestive of severe depression, raised blood pressure at follow-up representing undiagnosed or uncontrolled hypertension, and detectable viral load at follow-up representing possible adherence problems or treatment failure.

### Cost-effectiveness economic evaluation and modelling of long-term costs and effects

We will use the adapted Service Utilisation Questionnaire to systematically collect resource-use data, including any inpatient care, consultations with health providers, use of medications and laboratory tests, and time and travel costs associated with service uptake. We will collect information on the financing sources for each of the service categories to allow for an estimation of the extent of private, out-of-pocket expenditures incurred by recruited participants and their families. The unit costs or prices of these various resource inputs will be taken from previously conducted costing studies in South Africa [81] and from WHO-CHOICE data [82]. We will obtain medication costs from the Primary Care Guideline App published by the National Department of Health in South Africa [83]. Time loss from work may be affected by the interventions and we will measure this at baseline and follow-up and will value it using average wage rates. We will add the above costs to those of the actual intervention, which will be determined using an ‘Active Ingredients’ approach and allocated to trial participants using responses derived from the Service Utilization Questionnaire and linkage to related databases (e.g. counselling records). We will derive these from activity data (session provided per patient) and therapy unit costs (based on salaries, overheads and working hours). We will then make cost comparisons using bootstrapping methods to account for likely skewness in the data distributions.

To examine issues of cost-effectiveness, we will first relate estimated service costs per case in the different arms of the trial to primary study outcomes (viral loads and PHQ-9 scores). Based on earlier studies of stepped care for depression in India [84], a decrease in overall resource use can be expected due to a drop in ineffective but financially burdensome visits to providers unqualified to offer appropriate depression care. Since health outcomes for the stepped care intervention are also expected to improve significantly, the intervention will ‘dominate’ usual care (i.e. better outcomes, less cost); such a hypothesis negates the need for a power calculation. If, however, costs are found to be higher in the intervention group, bootstrapped incremental cost-effectiveness ratios for PHQ-9 depression and viral load scores will be derived. Irrespective of whether point estimates demonstrate dominance, results will be plotted on a cost-effectiveness plane and presented as cost-effectiveness acceptability curves in order to show the probability of the intervention being cost-effective at a range of ‘willingness-to-pay’ threshold levels. We will conduct sensitivity analysis to take account of uncertainty and imprecision in the measurements, including multiple imputation models for missing values.

As a second stage, we will move beyond the time and space constraints of the trial and consider longer-term costs and effects of the trial intervention at the population level. For this, we will construct and populate a quantitative economic model of expected lifetime costs and health gains, based on the earlier Markov modelling studies of Bachmann [85] and Cleary [81], but adapted to better capture other health states and outcomes of interest (depression and other NCDs, in particular cardiovascular disease and diabetes). We will convert health improvements observed in the trial into years of healthy life gained at the population level in the newly developed mental, neurological and substance use module of the inter-UN OneHealth strategic planning and costing tool [86], specifically by multiplying observed effect size for the intervention by the target rate of treated prevalence in the population. We will similarly compute population-level costs in OneHealth by multiplying the resource use and cost per case by the number of cases treated in the population at target coverage levels.

### Process evaluation

Given the complex nature of the intervention, which involves changing clinical flow and diversifying clinical functions, we have planned an intense process evaluation that is informed by the Medical Research Council framework for process evaluation of complex interventions [87]. We will use mixed methods using a combination of quantitative process variables collected across the clusters as well as in-depth qualitative process evaluation interviews.

In order to understand factors that may influence variations in the main health outcome findings between the arms and across the clusters, we will assess both possible contextual factors as well as factors associated with the fidelity and quality of implementation of the intervention. We will gather robust indicators on fidelity and quality of implementation of the intervention across the clusters through the collection of process indicators on the following:

1. Coverage of training sessions for nurse providers in Clinical Communication skills (intervention arm only), basic PC101/Adult Primary Care (intervention and control arm) and enhanced mental health PC101 training sessions (intervention arm only);
2. The number of in vivo training sessions and supervision received by the lay behavioural health counsellors across the clusters;
3. The number of patients identified and referred for depression treatment to different providers within the collaborative care model (lay counsellor, doctor, mental health specialist);
4. Fidelity checks on the implementation of the counselling intervention whereby lay behavioural health counsellors will record at least one of each of the eight sessions. These recorded sessions will be rated using two independent psychological practitioners using a fidelity checklist covering micro-counselling skills, technical skills and how well the counsellor adheres to the steps in the counselling manual developed during the formative phase of the trial;
5. The number of counselling sessions received by each patient referred to the counsellor and who presented themselves for counselling.

To clarify causal mechanisms and identify contextual factors associated with variation in outcomes of the trial in the implementation and control arms as well as across the clusters, we will collect the following data:

1. Facility, district and community level profile data that will record all possible contextual factors that may influence variation in outcomes in the intervention and control clusters such as staff turn-over, drug stock outs, community protest action, clinic closures.
2. We will conduct qualitative process evaluation data through in-depth interviews post hoc on purposefully sampled clusters informed by 6 month depression outcome data in both the intervention and control facilities. This will allow for more in-depth investigation of the variations between arms and clusters that may emerge in the outcome data [88]. In-depth qualitative process evaluation interviews will be held with a convenience sample of service providers within the collaborative care model as well as with service users. For the latter, we will purposefully select participants using a sampling frame based on process indicators (intervention arm) and participant interview responses (control arm) of service provider referred to, as well as dosage of the intervention received. These interviews will be transcribed and translated where necessary with back translation checks applied, and analysed using framework analysis with the aid of NVivo 10 qualitative data analysis software. Framework analysis was originally developed for qualitative data analysis in applied policy analysis research [89], and provides a systematic structure for the analysis process, allowing for the use of a priori and emergent codes to be used in the analysis process and involves a number of steps, namely (1) reading and re-reading the transcripts; (2) the development of a coding framework based on the interview questions; (3) coding of the data, with emergent themes being added to the coding framework during this coding process; (4) summarizing the responses from the respondents across each theme; and (5) interpreting the final themes in light of what they suggested for service planning and interventions.

### Ethics and dissemination

We obtained permission to enrol and randomise the clinics in the trial from the relevant district and provincial health department. We ask patients to provide consent for data collection and not randomisation as the clinic to which they are assigned is determined by where they live. We use a clearly defined and phased recruitment process to enrol patients in clinics (Additional file 4) to minimise the chance of inadvertent identification of patient participants as being HIV positive within clinic waiting rooms, and to manage the risk of discomfort posed by the more sensitive content of the PHQ-9 questionnaire. Fieldworkers will explain the trial information sheets in detail to prospective patients (Additional file 5). We specifically require patients to declare that they understand the consent to be voluntary and that it can be withdrawn at any time, to agree to the use of their personal information for study purposes, to undergo three interviews (baseline, 6 and 12 months), to allow research staff to access their medical records, and to permit future analysis of their de-identified data provided such analyses are reviewed and approved by a research ethics committee. Patient level data will be made available from the corresponding author at lara.fairall@uct.ac.za at the time of publication of the main trial results. Consent for data sharing will not be explicitly specified in the consent process but the presented data will be anonymised and risk of identification is low.

Ethical approval for the trial was obtained from the University of Cape Town Human Research Ethics Committee (reference number 211/2013), King’s College London Research Ethics Office (reference number PNM/12/13-159), the University of Kwazulu-Natal Biomedical Research Ethics Committee (reference 211/2013), and the North West Provincial Department of Health.

We will first submit the results of the trial for peer-review publication within 1 year of the last patient being followed-up. We will acknowledge substantive contributions to the design, conduct, analysis and interpretation of the trial through co-authorship and criteria for contributorship specified by the International Committee of Medical Journal Editors will be applied [90]. We have planned separate publications to document the intervention, and the economic and process evaluations. We will feed back the results to the district and provincial health departments through a series of local workshops at district and provincial level. They will be disseminated and debated with the South African National Department of Health through the Project Steering Committee (for composition see Additional file 1). Policy briefs and a video summary will be used to disseminate results to a wider audience.

## Discussion

The overall purpose of the CobALT trial is to provide evidence on the effectiveness and cost-effectiveness of scalable interventions to reduce the treatment gap posed by the growing burden of depression among adults on lifelong ART. Specifically, the trial is testing the effectiveness of a health systems intervention, based on a localisation of the World Health Organization’s Mental Health Gap Action Programme guidelines, integrated into an existing, tested and scaled-up training programme to strengthen nurse-led primary care. This approach uses a collaborative care model led by nurses, and complemented by referral services to primary healthcare doctors and clinic-based behavioural health counsellors using a manualised depression counselling intervention. The trial design is a large pragmatic trial conducted in 40 nurse-led primary care clinics across two rural districts of South Africa with outcomes measured on 2000 adults on ART who have screened positive for depression. It will provide definitive evidence of the effect of the intervention on mental and physical outcomes and highlight the importance of addressing the mental health needs in securing and sustaining the gains made by the scale-up of ART programmes.

### Trial status

Follow-up interviews are complete in the Dr Kenneth Kaunda district and are in progress in the Bojanala district.

## Additional files

Additional file 1: Administration file. (DOCX 40 kb)

Additional file 2: SPIRIT 2013 Checklist. (DOC 125 kb)

Additional file 3: PC2101 pages. (DOCX 23277 kb)

Additional file 4: Pre-consent processes. (DOCX 41 kb)

Additional file 5: Consent forms. (DOCX 492 kb)

Additional file 6: Baseline questionnaire. (PDF 7265 kb)

## Abbreviations

ART: Antiretroviral treatment
CobALT: Comorbid Affective Disorders, AIDS/HIV, and Long Term Health
DSMB: Data and Safety Monitoring Board
DSM-IV: Diagnostic and Statistical Manual of Mental Disorders
ICC: Intraclass correlation coefficient
ICSM: Integrated Clinical Services Management
IRB: Institutional Review Board
LMICs: Low- and middle-income countries
NCDs: Non-communicable diseases
NHI: National Health Insurance
PC101: Primary Care 101
PHC: Primary healthcare
PHQ-9: Patient Health Questionnaire
PRIME: Programme for Improving Mental Health Care
RCT: Randomised controlled trial
STIs: Sexually transmitted infections
