# Supplementary figures and images for: Collaborative care for the detection and management of depression among adults receiving antiretroviral therapy in South Africa: study protocol for the CobALT randomised controlled trial
Source: Trials. 2018 Mar 22;19:193. doi: 10.1186/s13063-018-2517-7 (PMC5863840; doi:10.1186/s13063-018-2517-7)

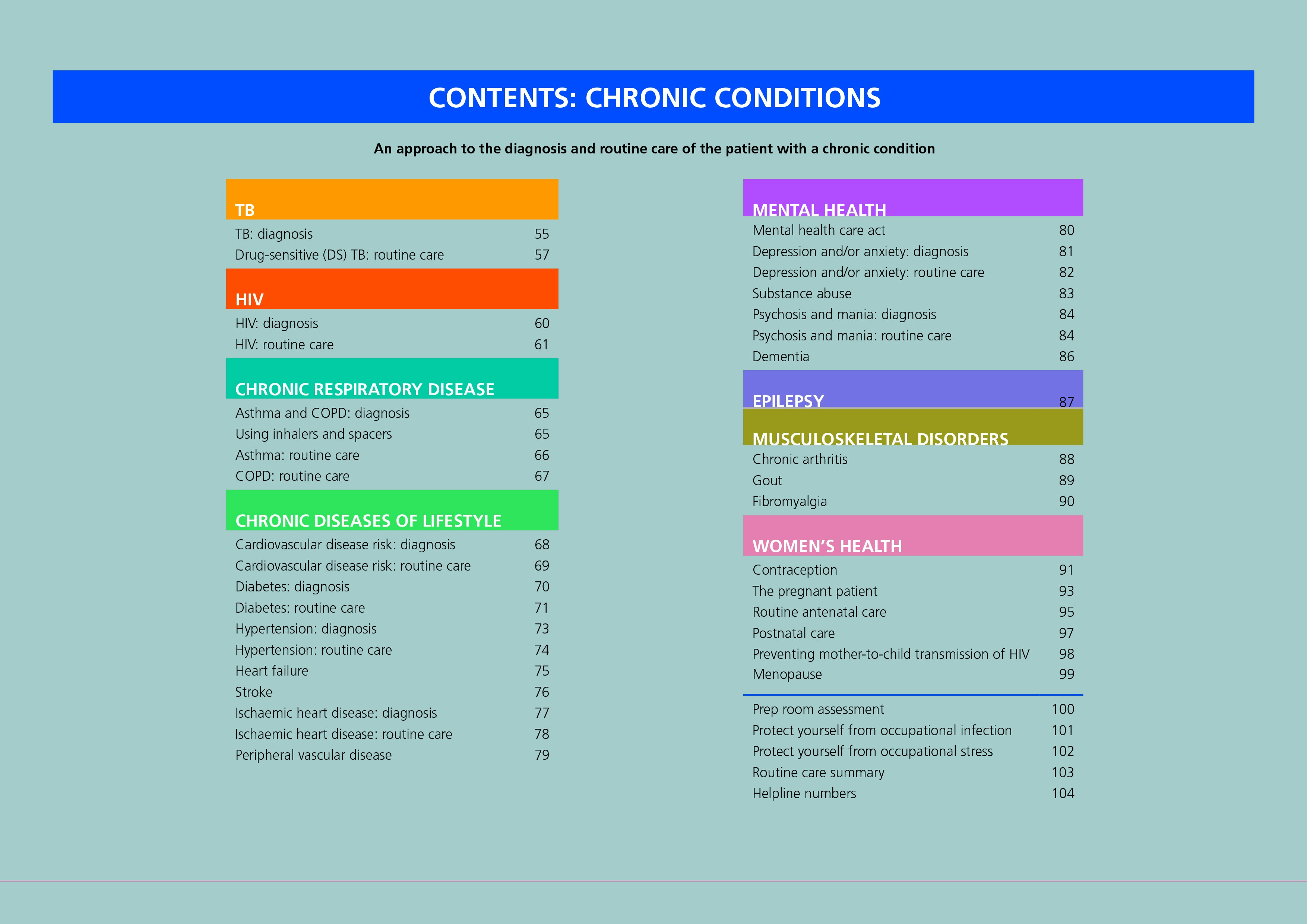


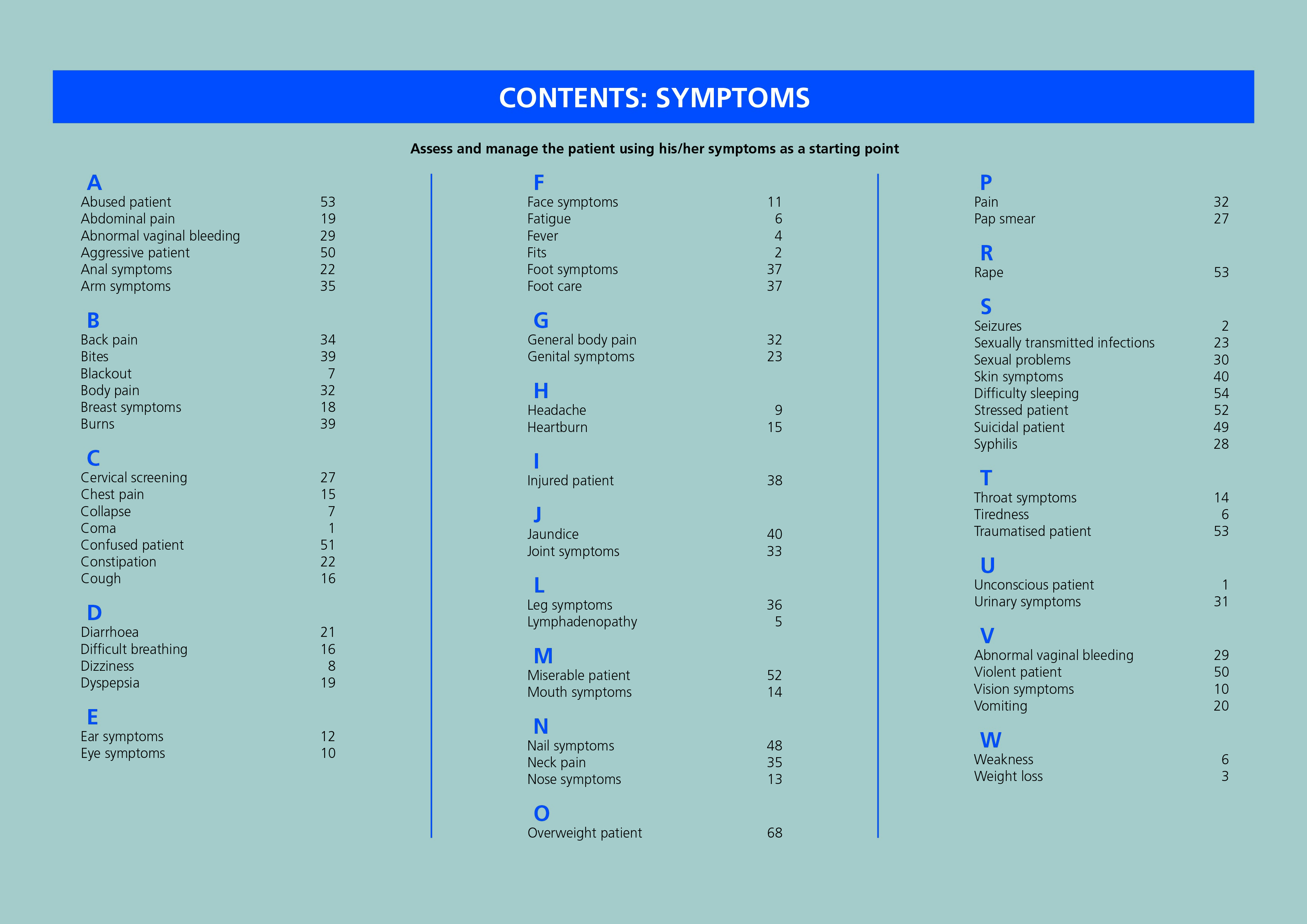


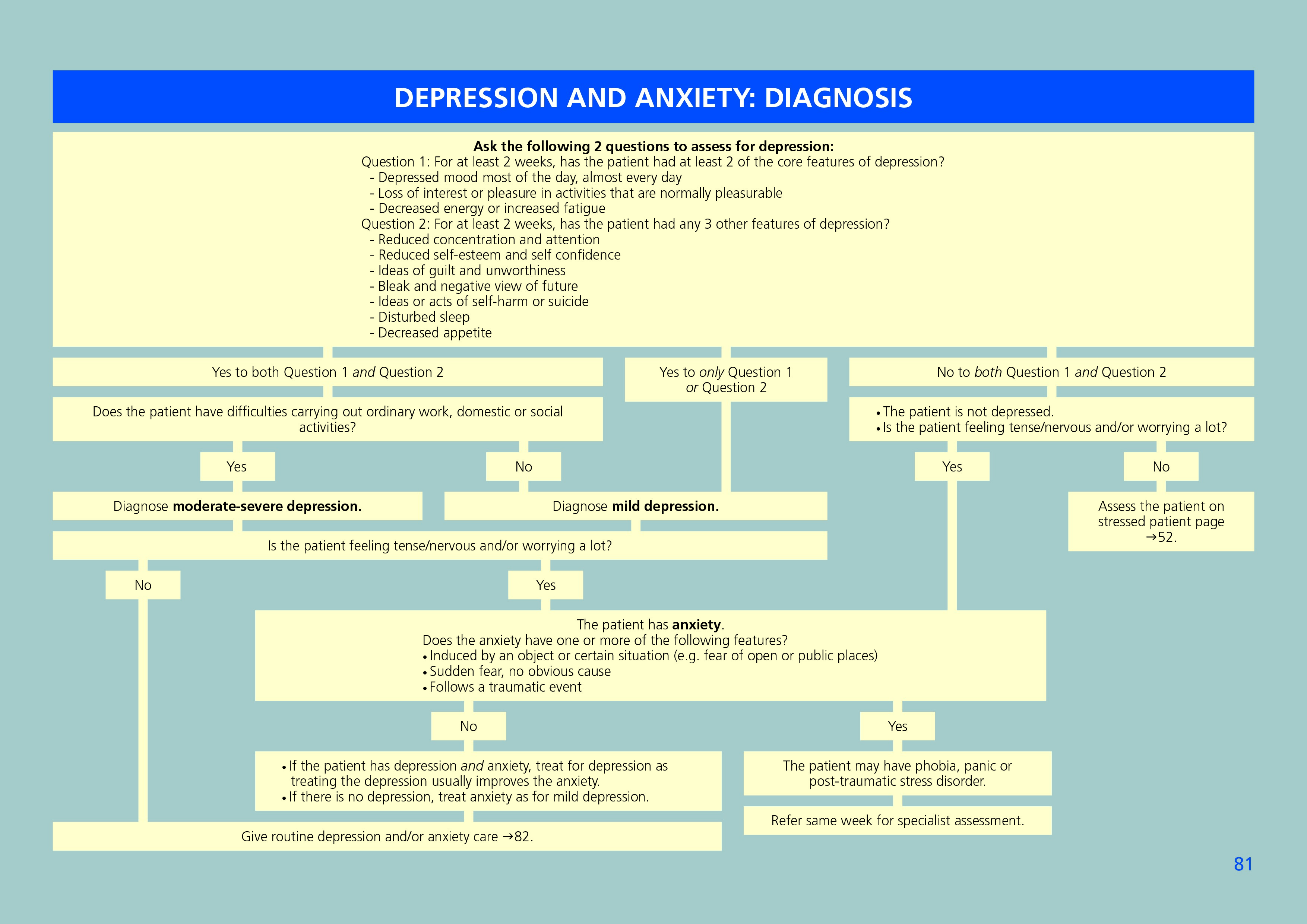


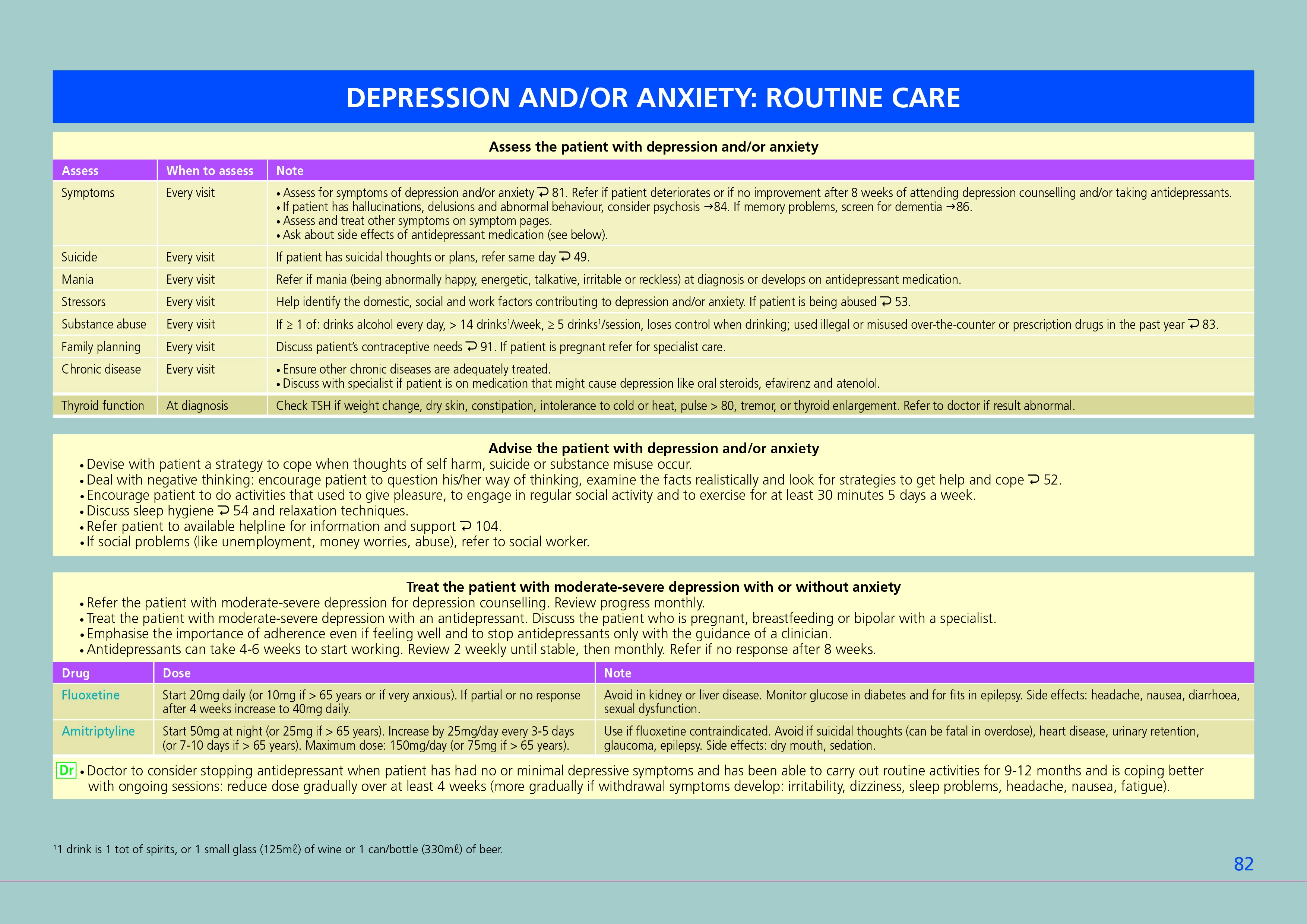


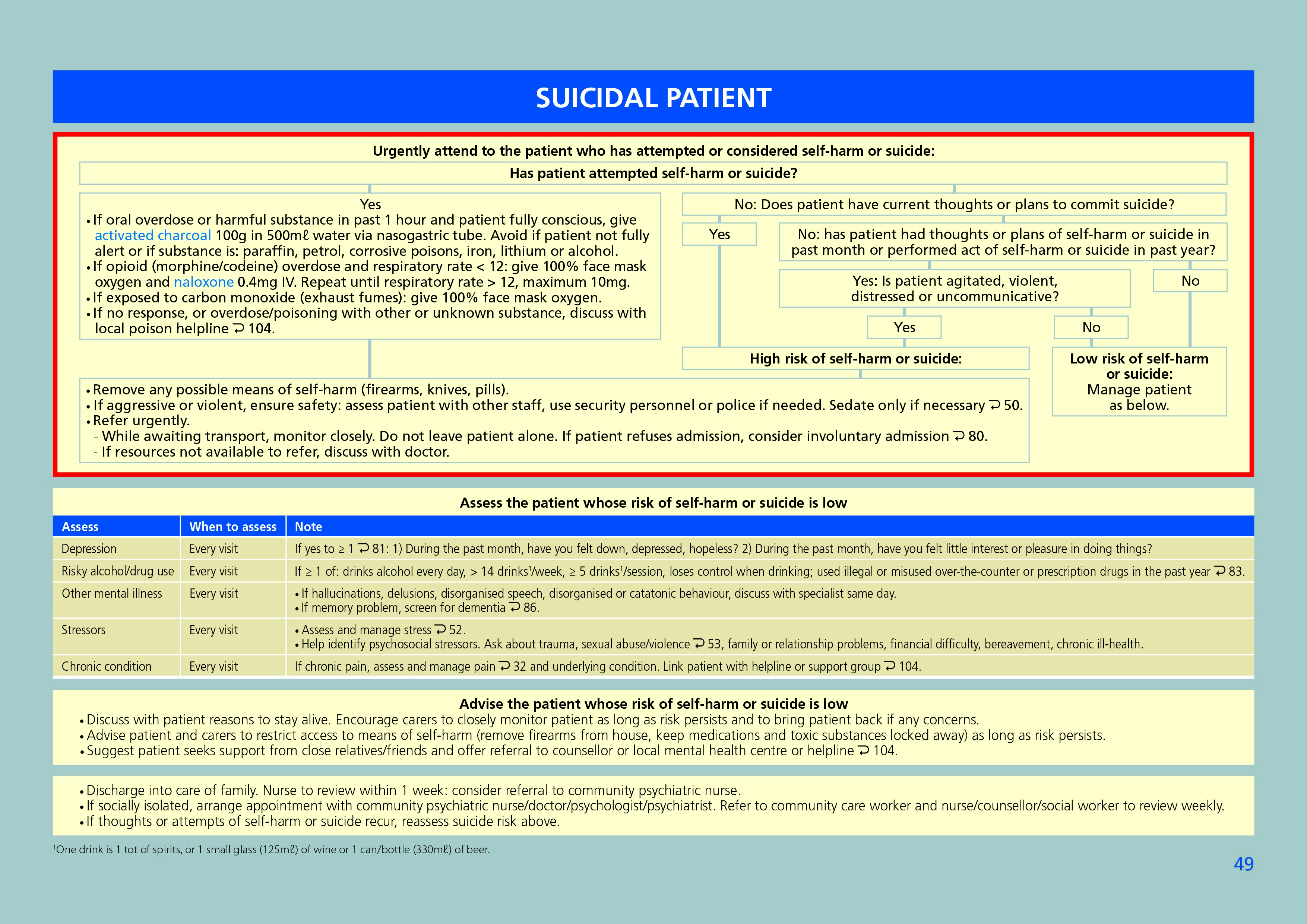


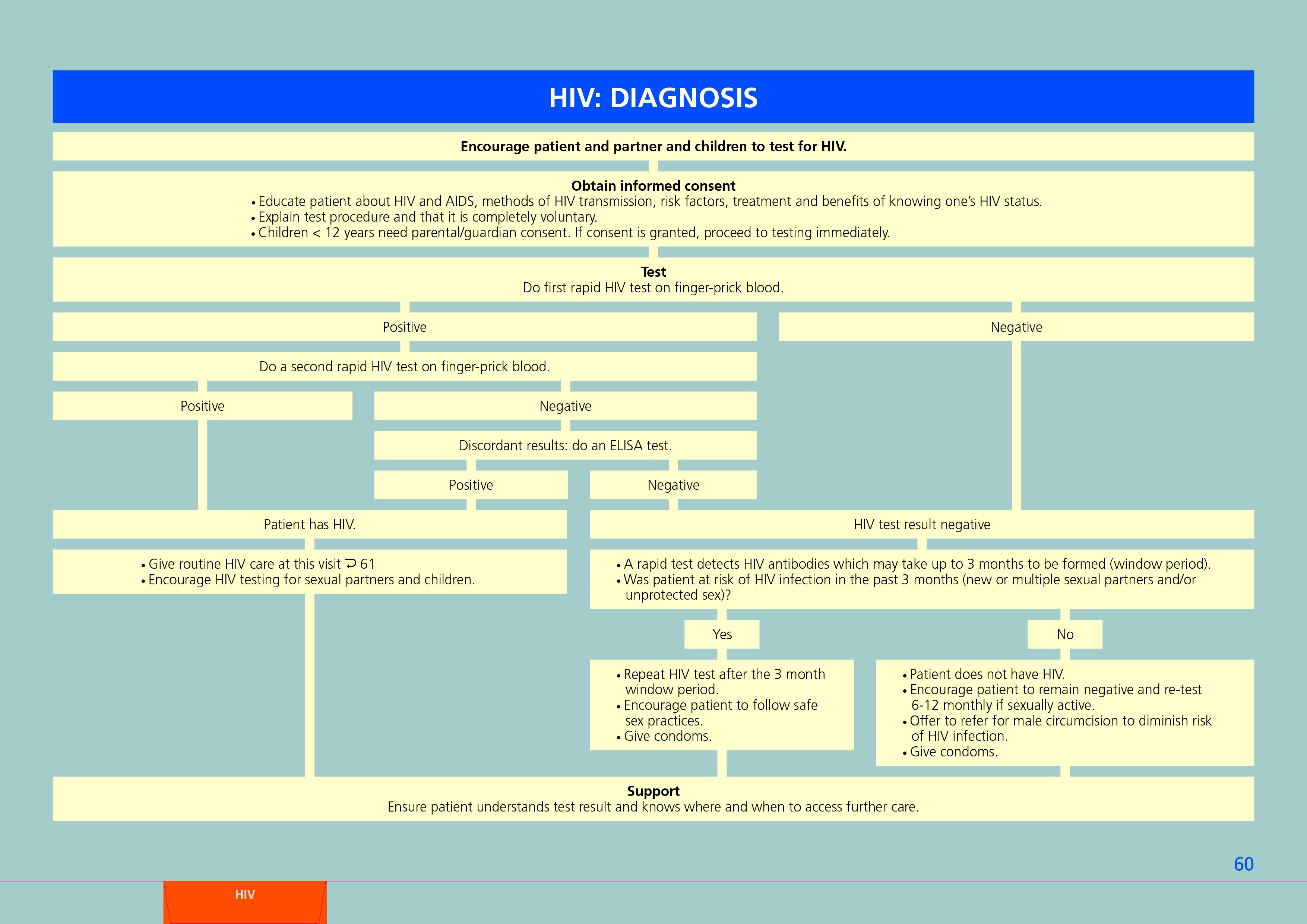


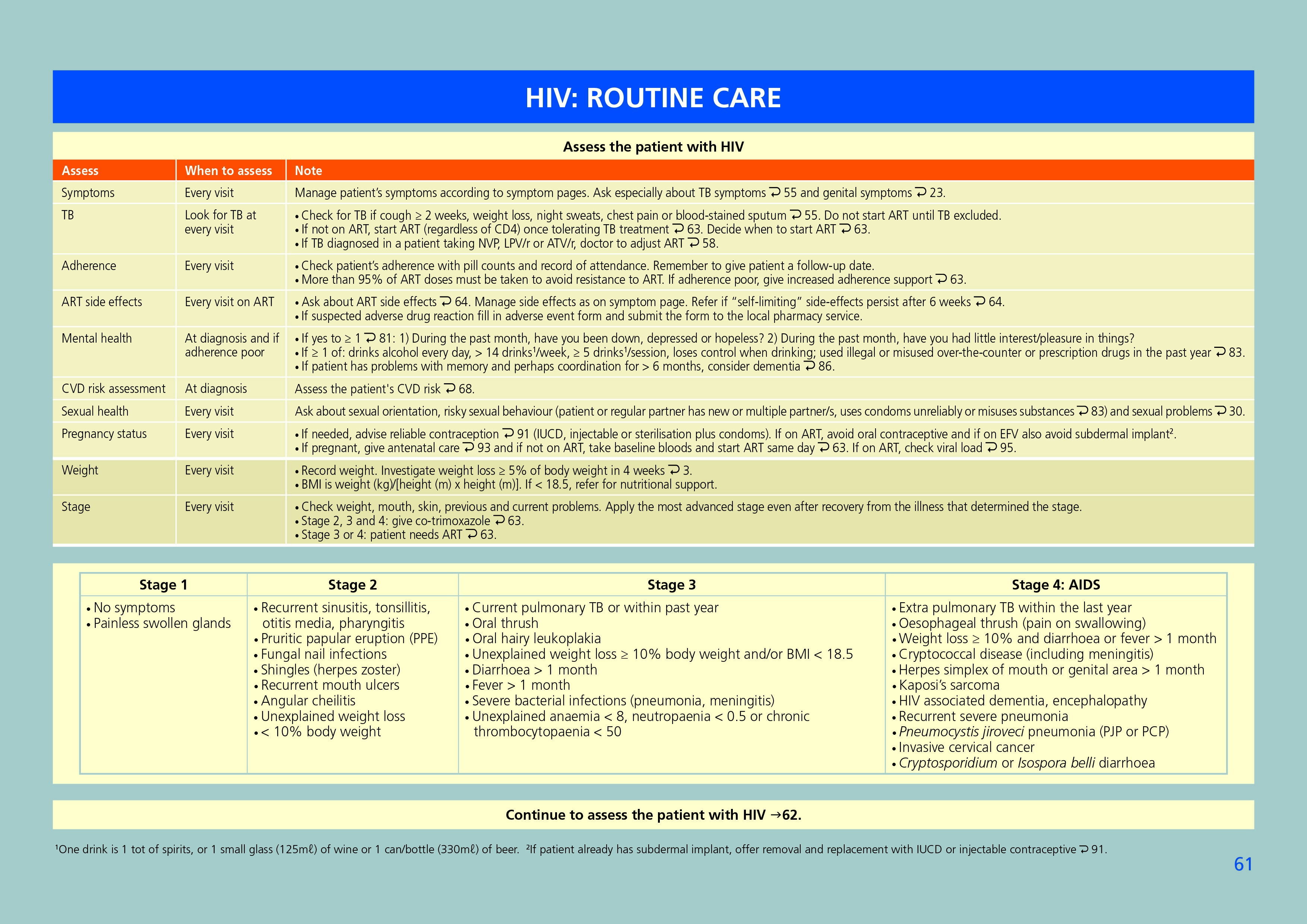


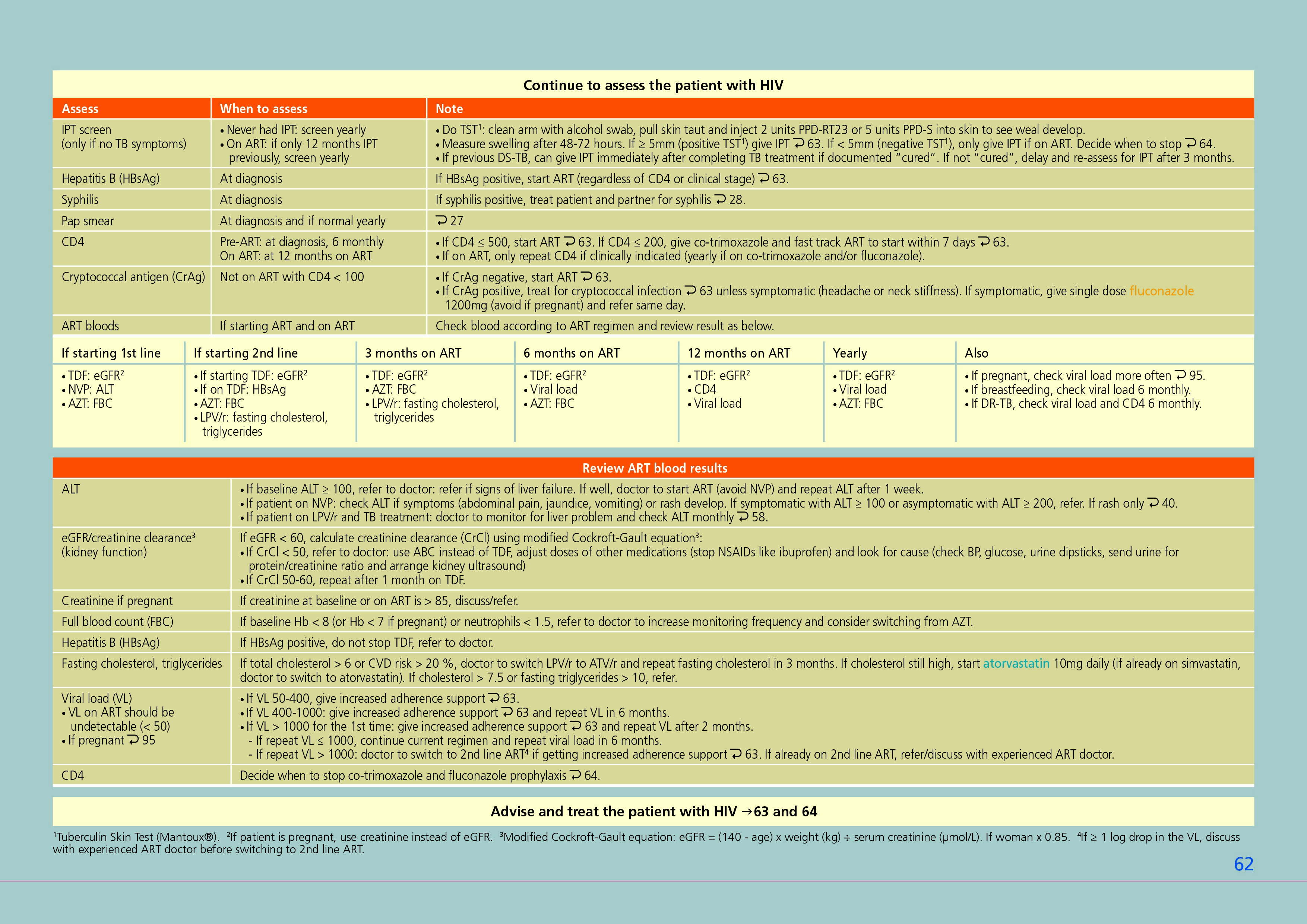


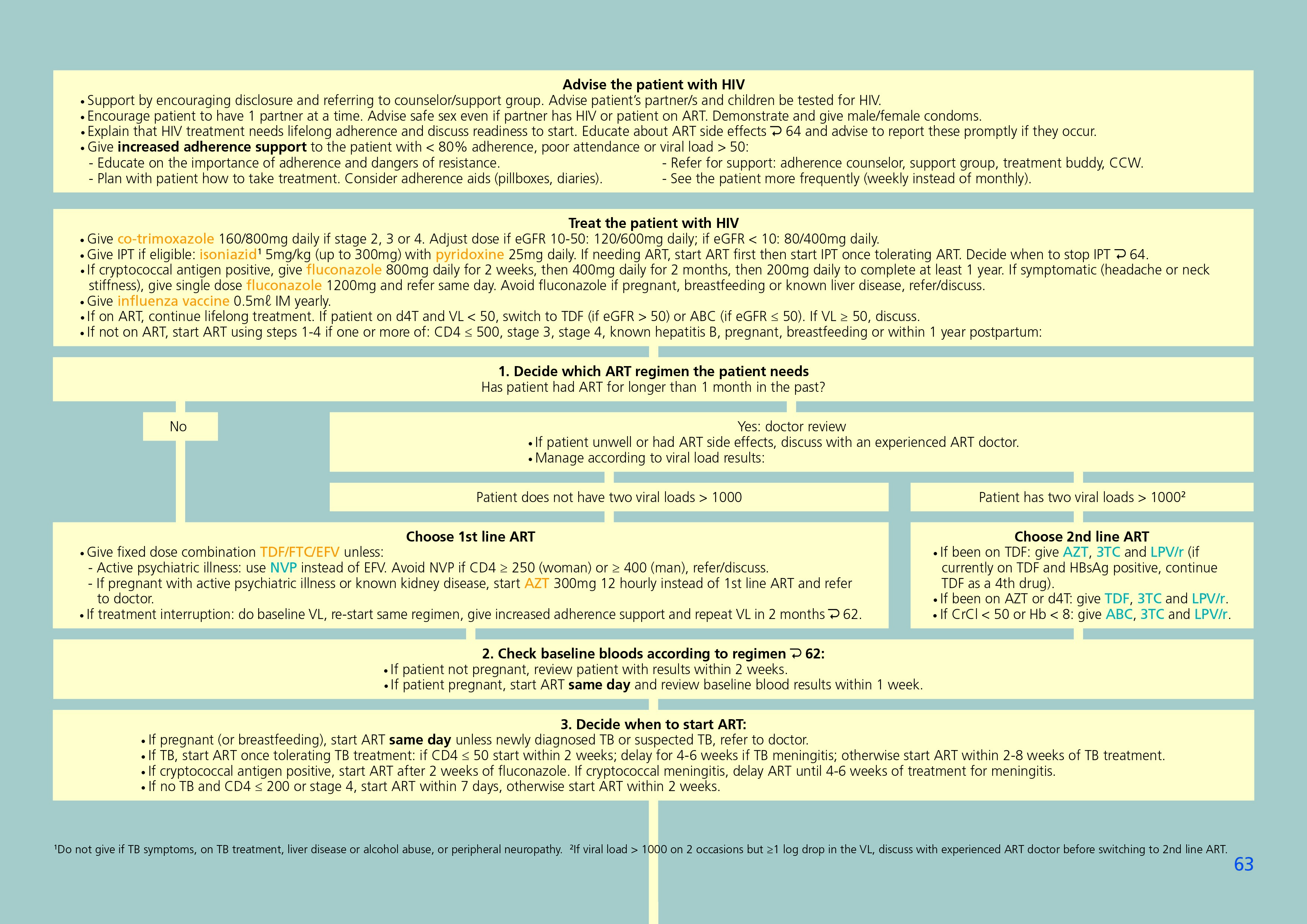


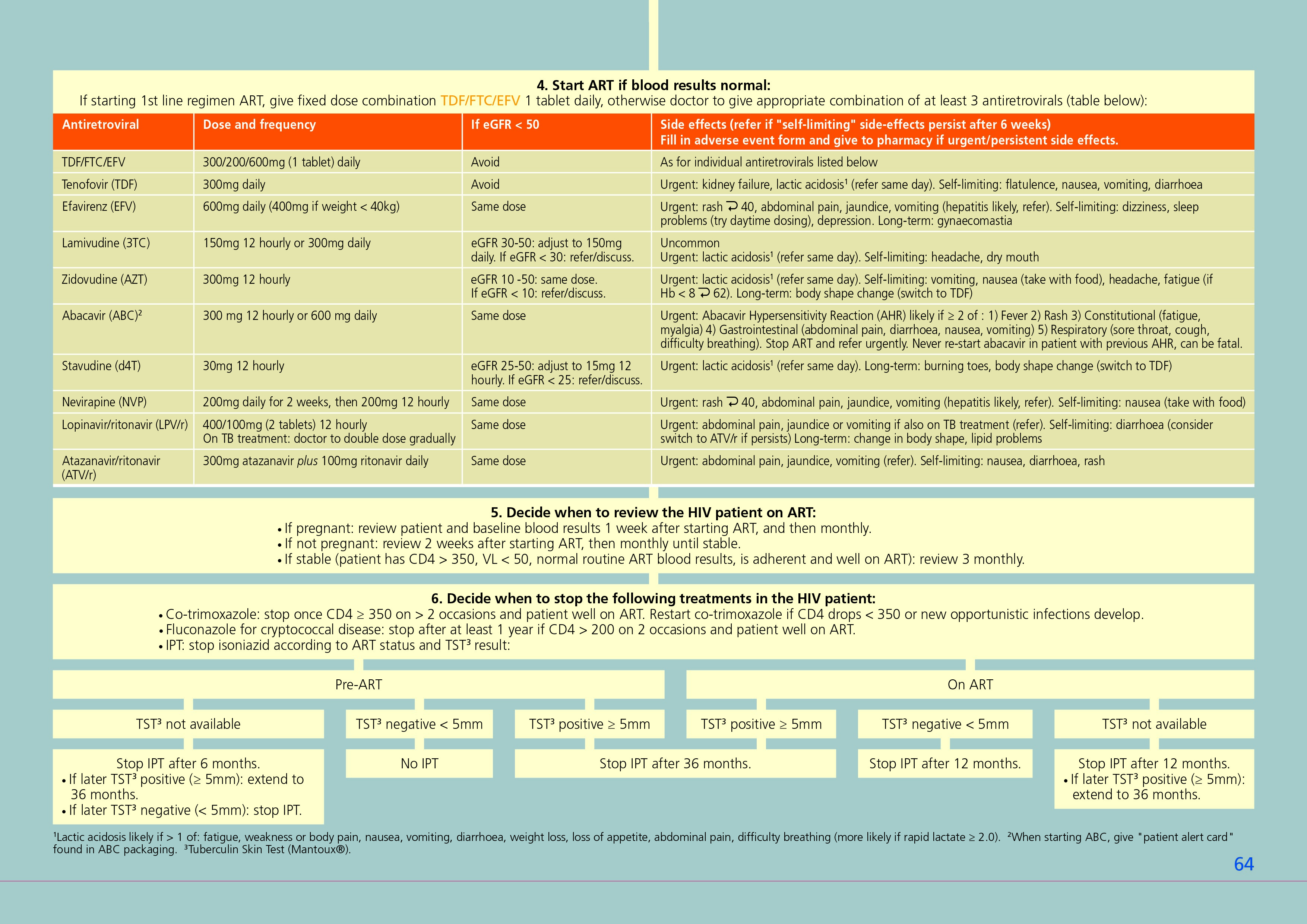


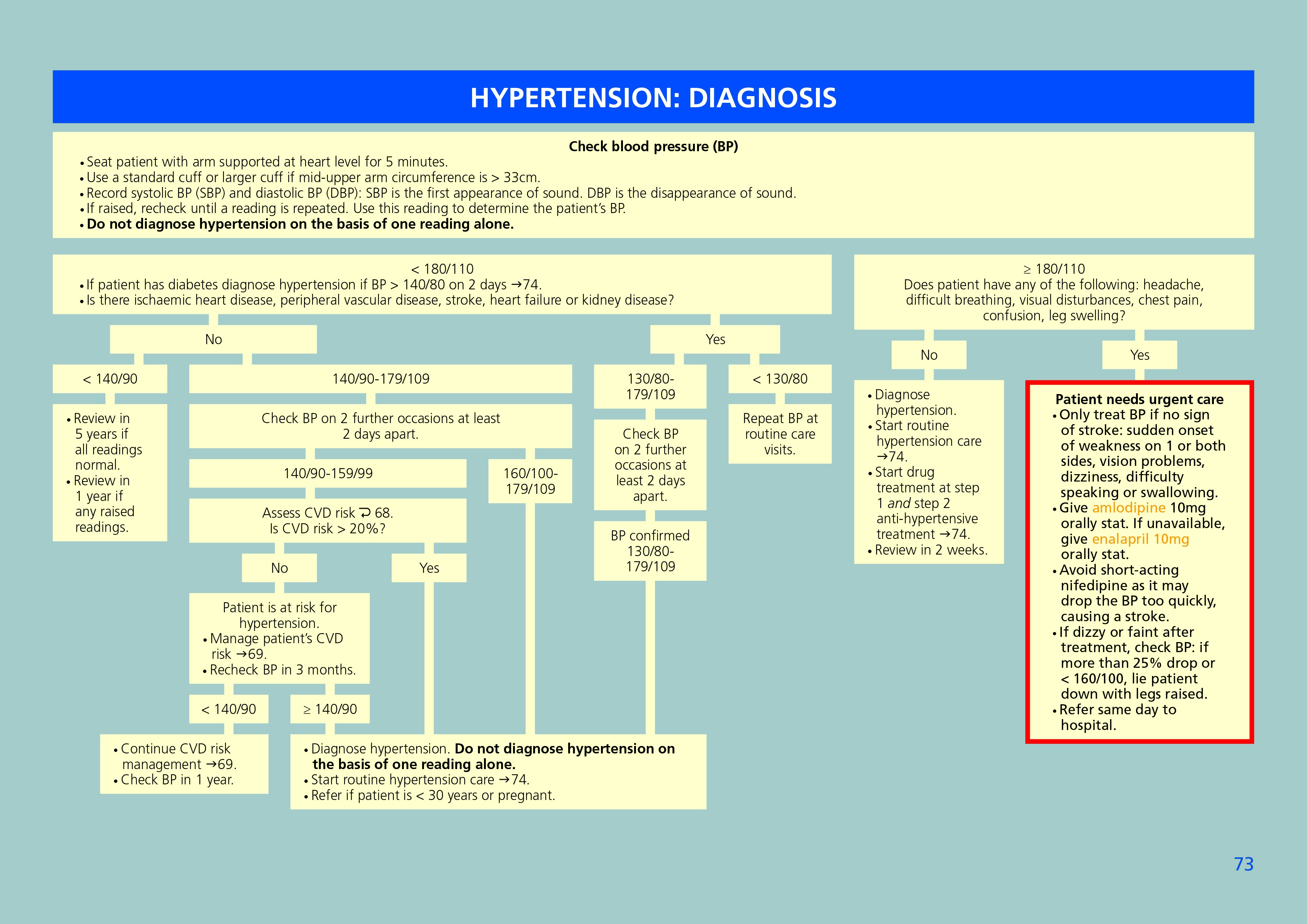


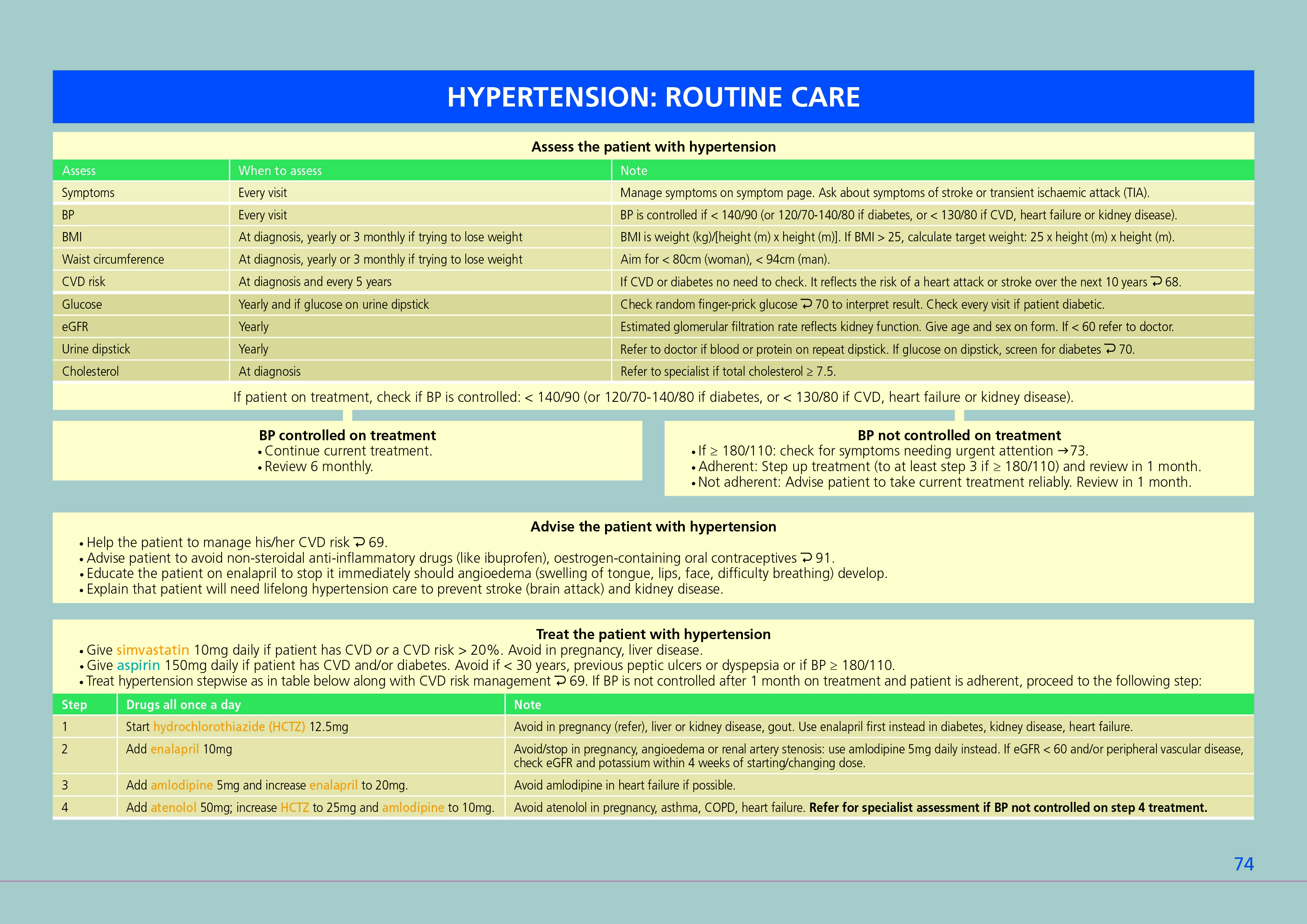


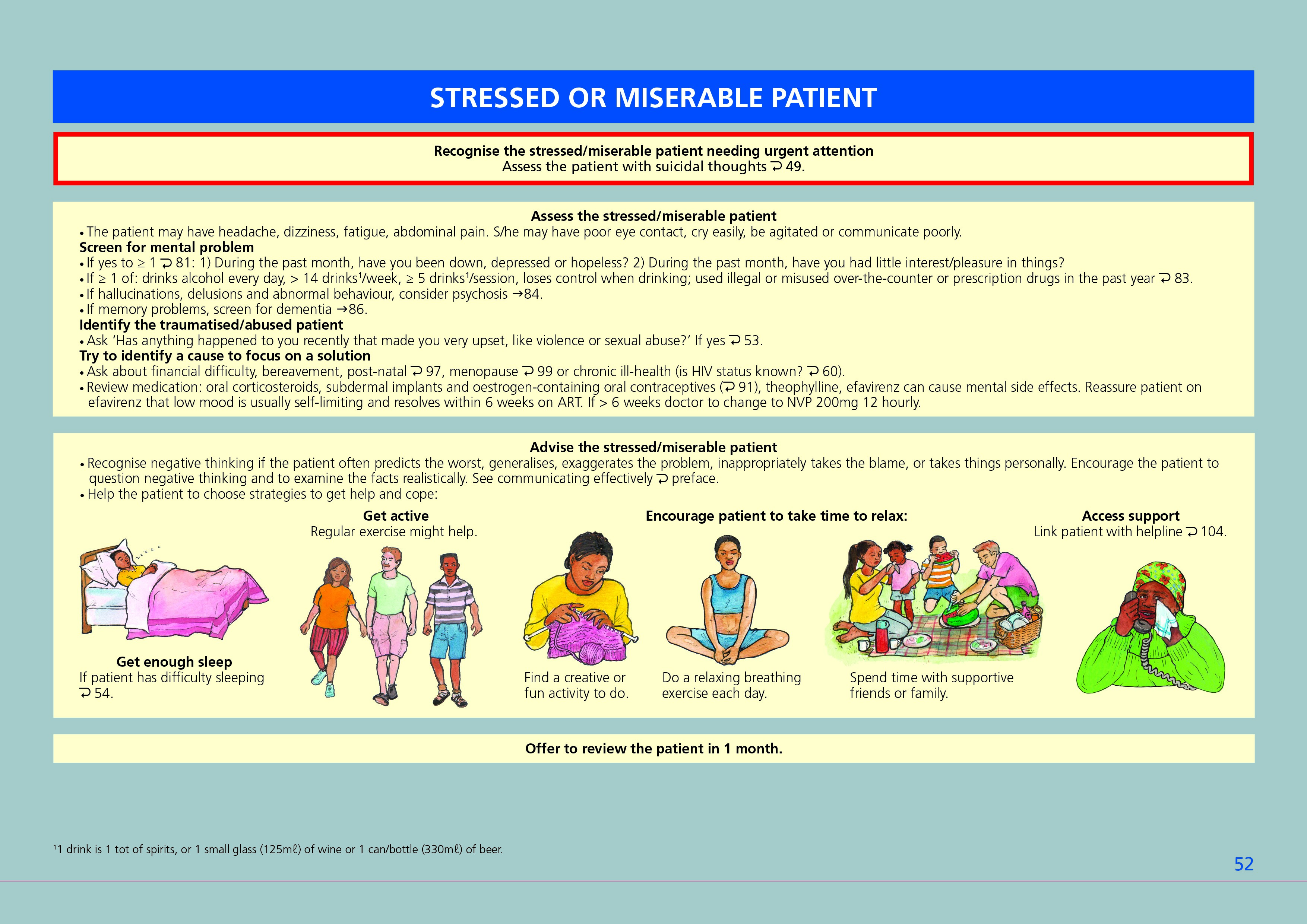

Supplement: Supplementary file 3 — PC2101 pages. (DOCX 23277 kb) [file 13063_2018_2517_MOESM3_ESM.docx]
