# Supplementary material for: Collaborative care for the detection and management of depression among adults receiving antiretroviral therapy in South Africa: study protocol for the CobALT randomised controlled trial
Source: Trials. 2018 Mar 22;19:193. doi: 10.1186/s13063-018-2517-7 (PMC5863840; doi:10.1186/s13063-018-2517-7)
Supplement: Supplementary file 5 — Consent forms. (DOCX 492 kb) [file 13063_2018_2517_MOESM5_ESM.docx]

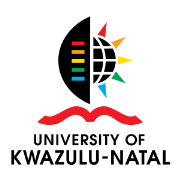
**
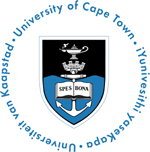

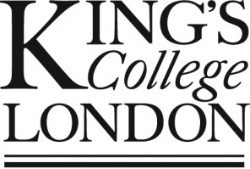
 MENTAL HEALTH STUDY**

**Information Sheet for Participants**

**You will be given a copy of this information sheet**

KCL REC reference number**: PNM/12/13-159. UCT HREC reference number: 211/2013. UKZN BREC reference number: BFC049/15; Date of this version: 16/03/2015**

We would like to invite you to participate in a research study. You should only take part if you want to; choosing not to take part will not disadvantage you in any way. Before you agree to take part, you need to understand what it involves. Please read the following information carefully and feel free to ask us if anything is not clear.

**Purpose of the study**

The purpose of the study is to evaluate a new programme aimed at improving the detection and management of depression and other conditions in patients attending antiretroviral therapy (ART) clinics. The programme comprises a guideline and training for nurses and doctors, and a counseling service for patients who are found to be suffering from depression.

The Study will be taking place in the Dr Kenneth Kuanda and Bojonala districts and we plan to enroll about 2000 people. It will be conducted by researchers from King’s College (London) and the Universities of Cape Town and KwaZulu-Natal, and is supported by the Department of Health. The study has been approved by registered human research ethics committees and is funded by the National Institutes of Health, USA.

We are looking for people to take part in the study who are attending ART clinics, over 18 years of age and planning to stay in the area for the next year. We will use a brief questionnaire to identify whether people are eligible for the study.  This questionnaire has been used in research studies to screen for people who may be at risk of depression.

**What are we trying to learn?**

We want to know whether the new programme results in better detection and management of depression as well as other conditions such as high blood pressure, and whether it improves HIV outcomes such as lower viral loads.

**What are the possible benefits of participating in this study?**

The information we obtain from the study will help us understand ways to improve the diagnosis and management of depression and other common conditions in people attending ART clinics. We intend to publish the findings so that others can also learn from the study.

**What are the possible drawbacks or discomforts in participating in this study?**

We will ask you to be interviewed on three occasions over the course of a year. Each interview should take approximately 20 to 40 minutes.

We may ask you to have a blood sample taken to measure your viral load at your first interview. We will definitely ask you to have another blood sample at your last interview. This will be the only discomfort in this study. Risk of infection will be minimized by using sterile procedures, and all blood samples will be taken by suitably qualified people.

There is the possibility that you may have a mental health problem yourself or that participation in the study may remind you of a time that you had such a problem. If you get distressed by this, we will refer you for clinical assessment.

**Do I have to participate in this study?**

No. It is up to you to decide whether to take part or not. If you decide to take part you are still free to withdraw from the study at any time and without giving a reason. Should you decide not to take part, or if you withdraw from the study, this will in no way affect the care you receive at the clinic. Should you agree to participate, we will ask you to sign the attached consent form.

**What will happen to me if I participate?**

We will ask you some questions using a structured questionnaire. The questions will be varied, but will include asking about smoking, alcohol use and your mental health. We will then measure your height, weight, and the width around your waist using a tape measure with your clothes on. We will also measure your blood pressure.

After the interview, we will ask you to have a blood test from your arm. We will take about 5ml of blood (1 teaspoon). The blood will be used to measure your viral load. The needle may cause you a little discomfort, but it will be taken in the way blood is usually taken from you when you attend the clinic.

We will want to interview you again, at this clinic, 6 and 12 months after your first interview. The interviews will be similar, but we will only ask for the blood test at the first and last interview. You will be provided with a voucher to the value of R50 at each of the three interviews (this first interview, the 6 month interview and the 12 month interview) in lieu of your travel expenses.

We are also asking your permission to review your hospitalisation records should you be hospitalised during the course of the study, your laboratory results and records of care. We will ask you for your South African identity number if available. This will allow linkage with a research copy of the Department of Home Affair’s databases to track your vital status.

**Will the information remain confidential?**

Yes. Should you agree to take part in the study all identifiable records will be seen by the study researchers only. Information and results of the study that are shared with other researchers will not contain any identifiable information such as names or contact details.

**Contact details of the study staff:**

If you have any questions or require more information about this study, please contact one of the following study Principal Investigators:

Prof I. Petersen Dr L. Fairall

School of Psychology Knowledge Translation Unit

University of KwaZulu-Natal University of Cape Town Lung Institute

Tel: 031 260 7970 Tel: 021 406 6979

Email: [PETERSENI@ukzn.ac.za](mailto:PETERSENI@ukzn.ac.za) Email: [lara.fairall@uct.ac.za](mailto:lara.fairall@uct.ac.za)

Prof G. Thornicroft

Institute of Psychiatry, King’s College London

De Crespigny Park, London SE5 8AF, England

Email: [graham.thornicroft@kcl.ac.uk](mailto:graham.thornicroft@kcl.ac.uk)

If you have any complaints regarding this research study you may contact the Human Research Ethics Committee, Faculty of Health Sciences, University of Cape Town at 021 406 6492.


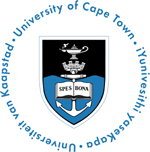

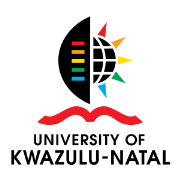


**
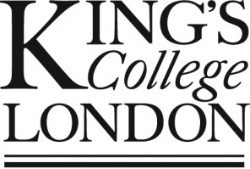
MENTAL HEALTH STUDY**

**Consent Form for Participants: please complete this form after**

**you have been through the information sheet about the study**

KCL REC reference number: **PNM/12/13-159. UCT HREC reference number: 211/2013. UKZN BREC reference number: BFC049/15**

Thank you for considering taking part in this study. If you have any questions arising from the information sheet, please ask before you decide whether to take part. You will be given a copy of the information sheet and consent form.

|  | Please tick or initial |
| --- | --- |
| I understand that if I decide at any time during the study that I no longer want to take part, I can notify the researchers and withdraw without having to give a reason. |  |
| I consent to the processing of my personal information for the purposes explained to me. |  |
| I agree to be interviewed at three time points: now, and 6 and 12 months later. |  |
| I agree that the research team may access my medical records for the purpose of this study. |  |
| I agree that the research team may use my data (information) for future research and understand that any such use would be reviewed and approved by a research ethics committee. In such cases, as with this project, data would not be identifiable in any report. |  |

Participant’s statement:

I,………………………………………………………………………………………………………

(Name of participant in block letters)

have read the information sheet and consent form, or they have been read to me. I understand what the study involves and have been given the opportunity to discuss it and ask questions. I voluntarily agree to take part in this research study.

…………………..……………………………… …………

Signature of Participant Date

Investigator’s statement:

I…………………………………………………………………………………………………….

(Name of investigator in block letters)

confirm that I have carefully explained the nature and purpose of the study to the participant named above.

…………………………………………………… …………

Signature of investigator Date

**Counselling and Chronic Conditions Study [HYPERTENSION]**


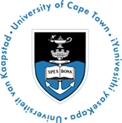

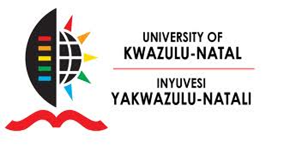


**Information Sheet for Participants**

**You will be given a copy of this information sheet**

We would like to invite you to participate in a research study. You should only take part if you want to; choosing not to take part will not disadvantage you in any way. Before you agree to take part, you need to understand what it involves. Please read the following information carefully and feel free to ask us if anything is not clear.

**Purpose of the study**

The purpose of the study is to evaluate a new programme aimed at improving the detection and management of depression and other conditions in patients attending chronic care clinics. The programme comprises a guideline and training for nurses and doctors, and a counseling service for patients who are found to be suffering from depression.

The Study will be taking place in the Dr Kenneth Kuanda district and we plan to enroll about 1000 people. It will be conducted by researchers from the Universities of Cape Town and KwaZulu-Natal, and is supported by the Department of Health. This study is funded by the National Institutes of Health, USA.

We are looking for people to take part in the study who are attending chronic care clinics, over 18 years of age and planning to stay in the area for the next year. The study will take place from 13 April 2015 to 30 November 2016. We will use a brief questionnaire to identify whether people are eligible for the study.  This questionnaire has been used in research studies to screen for people who may be at risk of depression.

**What are we trying to learn?**

We want to know whether the new programme results in better detection and management of depression as well as other conditions such as high blood pressure.

**What are the possible benefits of participating in this study?**

The information we obtain from the study will help us understand ways to improve the diagnosis and management of depression and other common conditions in people attending chronic care clinics. If you agree to participate you will be provided with a voucher to the value of R50-00 for each interview including this first interview, 6 and 12 month interviews for your time. The clinic that you attend will be allocated to one of two groups. One group of clinics will receive the intervention, and one group of clinics will not receive the intervention. If you agree to participate you may or may not receive the treatment depending on which group your clinic is allocated to.

**What are the possible drawbacks or discomforts in participating in this study?**

We will ask you to be interviewed on three occasions over the course of a year. Each interview should take approximately 20 to 40 minutes.

There is the possibility that you may have a mental health problem yourself or that participation in the study may remind you of a time that you had such a problem. If you get distressed by this, we will refer you for clinical assessment.

**Do I have to participate in this study?**


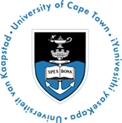

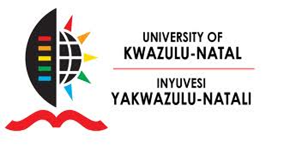


No. It is up to you to decide whether to take part or not. If you decide to take part you are still free to withdraw from the study at any time and without giving a reason. Should you decide not to take part, or if you withdraw from the study, this will in no way affect the care you receive at the clinic. Should you agree to participate, we will ask you to sign the attached consent form.

**What will happen to me if I participate?**

We will ask you some questions using a structured questionnaire. The questions will be varied, but will include asking about smoking, alcohol use and your mental health. We will then measure your height, weight, and the width around your waist using a tape measure with your clothes on. We will also measure your blood pressure. We will not take any blood or other tissue samples. We will record some information from your clinic chart/records.

We will want to interview you again, at this clinic 6 and 12 months after your first interview. The interviews will be similar. We are also asking your permission to review your hospitalisation records should you be hospitalised during the course of the study, your laboratory results and records of care. We will ask you for your South African identity number if available. This will allow linkage with a research copy of the Department of Home Affair’s databases to track your vital status. We will also require your contact details including your address and cellular phone numbers so that we can send you sms reminders or call you to remind you of your follow-up interviews and to arrange a venue for these interviews if you cannot attend the clinic to do so.

**Will my information remain confidential?**

Yes. Should you agree to take part in the study, all your information will be seen by the study researchers only.Information and results of the study that are shared with other researchers will not contain any identifiable (personal) information such as names or contact details. Every effort will be made to keep your information confidential. Although we will try to conduct this interview in a private room, it might happen that, during the course of your interview, another patient or clinic staff member comes into the room. (Should this happen, I will pause the interview when another person has entered the room, unless it is another member of the research team, such as my supervisor). However, if you express thoughts of suicide or intention to harm yourself or others, I will have to refer you to the clinic. In this case I will coordinate with my supervisor to immediately refer you to the clinic or to Dr Von Wiellig, the psychologist at the clinic

The possibility also exists that, despite the absence of identifying data, the clinic could be identified as one of the research sites due to a process of deduction from the public information about the PRIME project. This does not mean that you yourself will be identified but that the aggregate data from the study may be linked back to your clinic.

The information from your interview will be stored on a computer and protected with a password. Your data will be stored under password protection for up to five years on the PRIME-SA computers.


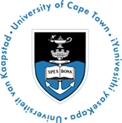

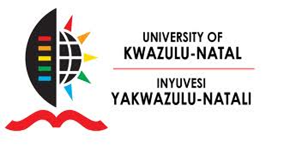


**How will we report this research?**

We intend to publish the findings so that others can also learn from the study .We will report our results and other aspects of the study in scholarly journals, conferences and to the Department of Health via policy briefs and other reporting structures.

This study has been ethically reviewed and approved by the University of KwaZulu-Natal Biomedical research Ethics Committee: **BFC049/15**

**Contact details of the study staff:**

If you have any questions or require more information about this study, please contact the following:

| **For questions related to the study** | **For Your rights as a research participant** |
| --- | --- |
| **The Principal Investigator, Professor Inge Petersen**  **Department of Psychology**  **Howard College**  **Private Bag X 54001**  **Durban**  **4000**  **KwaZulu-Natal, SOUTH AFRICA**  **Tel: 27 31 260 7970**  **Email: Peterseni@ukzn.ac.za** | **BIOMEDICAL RESEARCH ETHICS COMMITTEE (BREC)**  **Research Office, Westville Campus**  **Govan Mbeki Building**  **Private Bag X 54001  Durban  4000**  **KwaZulu-Natal, SOUTH AFRICA**  **Tel: 27 31 2604769 - Fax: 27 31 2604609**  **Email:** [**BREC@ukzn.ac.za**](mailto:ngwenyap@ukzn.ac.za) |

**Please complete this form after you have been through the information sheet and understand what your participation in this study entails.**

Thank you for considering taking part in this study. If you have any questions arising from the information sheet, please ask before you decide whether to take part. You will be given a copy of the information sheet and consent form.

**CONSENT FORM**

I, (write your name here), ________________________ have been informed about the PRIME-SA Study.

I understand the purpose and procedures of the study.


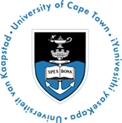

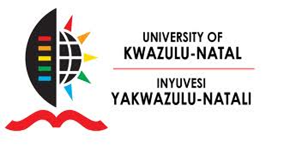

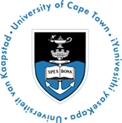

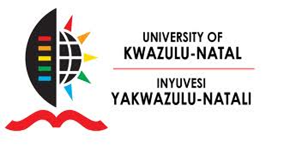


I have been given an opportunity to ask questions about the study and have had answers to my satisfaction.

I declare that my participation in this study is entirely voluntary and that I may withdraw at any time without affecting any treatment or care that I would usually be entitled to.

I understand that the clinic that I attend will be allocated to one of two groups. One group of clinics will receive the intervention, and one group of clinics will not receive the intervention. If I agree to participate I may or may not receive the treatment depending on which group my clinic is allocated to.

I have been informed about any available compensation or medical treatment if injury occurs to me as a result of study-related procedures.

If I have any further questions or concerns or queries related to the study or my rights as a research participant, I understand that I may contact the Principal Investigator Professor Inge Petersen or the Biomedical Research Ethics Committee at UKZN whose details are listed in the information sheet.

|  | Please tick or initial |
| --- | --- |
| I understand that if I decide at any time during the study that I no longer want to take part, I can notify the researchers and withdraw without having to give a reason. |  |
| I consent to the processing of my personal information including my SA identity document for the purposes explained to me. |  |
| I agree to be interviewed at three time points: now, and 6 and 12 months later. |  |
| I agree that the research team may access my medical records for the purpose of this study. |  |
| I agree that the research team may measure my height, weight, and the width around my waist using a tape measure my your clothes on, and measure my blood pressure |  |
| I agree that the research team may use my data (information) for future research and understand that any such use would be reviewed and approved by a research ethics committee. In such cases, as with this project, data would not be identifiable in any report. |  |
| I consent to the research team contacting me via an agreed method such as telephone, home-visit or any other agreed method for follow-up interviews |  |

**Signature of Participant Date: Signature of Translator Date:**

**(If applicable)**

---------------------------- -------------- ---------------------------- -------------
