# Supplementary material for: Collaborative care for the detection and management of depression among adults receiving antiretroviral therapy in South Africa: study protocol for the CobALT randomised controlled trial
Source: Trials. 2018 Mar 22;19:193. doi: 10.1186/s13063-018-2517-7 (PMC5863840; doi:10.1186/s13063-018-2517-7)
Supplement: Supplementary file 6 — Baseline questionnaire. (PDF 7265 kb) [file 13063_2018_2517_MOESM6_ESM.pdf]

Baseline (English)

Last Modified by: Mobenzi Support - 18 **on** 20 Oct 2015 08:46:31 **Revision number:** 2431 **Field Count:** 388

Section 1. Intro

1.1 intro_0

This is the baseline survey. Press NEXT to start.

1.2 CalculatedAgeYears

Numeric

This field is not displayed on the device

1.3 meansbp_0

Numeric

This field is not displayed on the device

1.4 meandbp_0

Numeric

This field is not displayed on the device

1.5 ref_suicA_0

Numeric

This field is not displayed on the device, Value: 0

1.6 ref_suicB_0

Numeric

This field is not displayed on the device, Value: 0

1.7 CalculatedVLReference

Numeric

This field is not displayed on the device

1.8 ref_vl_0

Numeric

This field is not displayed on the device, Value: 0

Prerequisites
Skip when ***meansbp_0 (1.3)*** **Greater Than '180'** AND
Skip when ***meandbp_0 (1.4)*** **Equals '110'**

1.9 ref_bp_0

Numeric

This field is not displayed on the device, Value: 0

1.10 facilitycode_0

Please select your clinic

Expects a single option response (**required**), Default: Test Clinic

- ☐ Test Clinic [tst]

1.11 fw_code_0

[Interviewer Code]:

Expects a single option response (**required**)

- ☐ Alberth Sotyantya [AS]
- ☐ Aubrey Malebo [AM]
- ☐ Babalwa Zani [BZ]
- ☐ Bafedile Modise [BM]
- ☐ Buyisiwe Jaas [BJ]
- ☐ Daniella Georgeu-Pepper [DGP]
- ☐ Deanna Carter [DC]
- ☐ James Matlhabaphiri [JM]
- ☐ Lelethu Mahamba [LM]
- ☐ Keitumtetse Manche [KM]
- ☐ Montsheng Nqoi [MN]
- ☐ Mpho Molatsane [MM]
- ☐ Nomsa Mudzanani [NM]
- ☐ Odiseng (OJ) More [OJ]
- ☐ Petronella Makhale [PM]
- ☐ Primrose Mpaphane [PR]
- ☐ Selinah Sepeng [SS]
- ☐ Stokie Olifant [SO]
- ☐ Venessa Timmerman [VT]
- ☐ Vinolia Ntjikelane [VN]
- ☐ Zakaria Siwata [ZS]

1.12 sid_0

Numeric

This field is not displayed on the device, Value: 0

1.13 devicecode_0

Text

This field is not displayed on the device, Value: 01

1.14 sequence_0

Text

This field is not displayed on the device

1.15 test_0

Numeric

This field is not displayed on the device, Value: 0

1.16 eft_0

Numeric

This field is not displayed on the device

1.17 bmi_00

Decimal

This field is not displayed on the device, Value: 0

1.18 reached_end_0

Numeric

This field is not displayed on the device, Value: 0

Section 2. Embedded SID Html

2.1 SIDHTML

Text

This field is not displayed on the device

Section 3. Embedded Summary Html

3.1 SummaryHTML

Text

This field is not displayed on the device

Section 4. Eligibility

4.1 interview_date_0

Date of interview:

Expects a date response (**required**)

Constraints

*Response must be **Less Than or Equal 'DATENOW'** AND*

*Response must be **Greater Than or Equal 'DATEADD(DATENOW,-7)'***

4.2 preconsent_0

As you have heard from my assistant, we are conducting two studies to evaluate a programme and we are looking for people with certain criteria to take part. I would like to start by asking you a few questions to see whether you qualify to take part in either or both of the studies. Some of these questions are about your psychological well-being and may be sensitive. You are free to stop the interview at any time and this will not affect your usual care at the clinic. Are you willing to continue with the questionnaire?

Expects a single option response (**required**)

☐ Yes [1]

☐ No [0]

Branches

If response **Not Equal 'Yes [1]'** then skip to ***no_preconsent_0 (15.6)***

4.3 birthdate_0

What is your date of birth?

Expects a date response (**required**)

4.4 SetCalculatedAgeYears

Operator

This field is not displayed on the device, Operator: Set(**CalculatedAgeYears (1.2)** , ROUND(DIVISION OF (DAYSBETWEEN(Q513075, DATENOW) ,365) ,2))

4.5 age_confirm_0

Please confirm that **CalculatedAgeYears (1.2)** is your correct age.

Expects a single option response (**required**)

☐ Yes [1]

☐ No [0]

Branches

If response **Equals 'No [0]'** then skip to ***birthdate_0 (4.3)***

Prerequisites

Skip when ***CalculatedAgeYears (1.2)* Greater Than '18.00'** OR

Skip when ***CalculatedAgeYears (1.2)* Equals '18.00'**

4.6 age_under18_0

Your age is less than 18 therefore you do not qualify to participate. Please select PROCEED to continue.

Expects a single option response (**required**)

☐ Proceed [1]

Branches

If response **Equals 'Proceed [1]'** then skip to ***age_less18_0 (15.7)***

4.7 area_0

Are you planning to stay in the area for the next year?

Expects a single option response (**required**)

- ☐ Yes [1]
- ☐ No [0]

Branches

If response **Equals 'No [0]'** then skip to ***no_area_0 (15.8)***

Section 5. PHQ

5.1 phq_intro_0

Over the last 2 weeks, how often have you been bothered by any of the following problems?

5.2 phq1_0

Over the last 2 weeks, how often have you been bothered by: Little interest or pleasure in doing things

Expects a single option response (**required**)

- ☐ 0 days [0]
- ☐ 1 - 7 days [1]
- ☐ 8 - 11 days [2]
- ☐ 12 -14 days [3]

5.3 phq2_0

Over the last 2 weeks, how often have you been bothered by: Feeling down, depressed, or hopeless

Expects a single option response (**required**)

- ☐ 0 days [0]
- ☐ 1 - 7 days [1]
- ☐ 8 - 11 days [2]
- ☐ 12 -14 days [3]

5.4 phq3_0

Over the last 2 weeks, how often have you been bothered by: Trouble falling or staying asleep, or sleeping too much

Expects a single option response (**required**)

- ☐ 0 days [0]
- ☐ 1 - 7 days [1]
- ☐ 8 - 11 days [2]
- ☐ 12 -14 days [3]

5.5 phq4_0

Over the last 2 weeks, how often have you been bothered by: Feeling tired or having little energy

Expects a single option response (**required**)

- ☐ 0 days [0]
- ☐ 1 - 7 days [1]
- ☐ 8 - 11 days [2]
- ☐ 12 -14 days [3]

5.6 phq5_0

Over the last 2 weeks, how often have you been bothered by: Poor appetite or overeating

Expects a single option response (**required**)

- ☐ 0 days [0]
- ☐ 1 - 7 days [1]
- ☐ 8 - 11 days [2]
- ☐ 12 -14 days [3]

5.7 phq6_0

Over the last 2 weeks, how often have you been bothered by: Feeling bad about yourself - or that you are a failure or have let yourself or your family down

Expects a single option response (**required**)

- ☐ 0 days [0]
- ☐ 1 - 7 days [1]
- ☐ 8 - 11 days [2]
- ☐ 12 -14 days [3]

5.8 phq7_0

Over the last 2 weeks, how often have you been bothered by: Trouble concentrating on things, such as reading the newspaper or watching television

Expects a single option response (**required**)

- ☐ 0 days [0]
- ☐ 1 - 7 days [1]
- ☐ 8 - 11 days [2]
- ☐ 12 -14 days [3]

5.9 phq8_0

Over the last 2 weeks, how often have you been bothered by: Moving or speaking so slowly that other people could have noticed? Or the opposite - being so fidgety or restless that you have been moving around a lot more than usual

Expects a single option response (**required**)

- ☐ 0 days [0]
- ☐ 1 - 7 days [1]
- ☐ 8 - 11 days [2]
- ☐ 12 -14 days [3]

5.10 phq9a_0

Over the last 2 weeks, how often have you been bothered by: Thoughts that you would be better off dead or of hurting yourself in some way

Expects a single option response (**required**)

- ☐ 0 days [0]
- ☐ 1 - 7 days [1]
- ☐ 8 - 11 days [2]
- ☐ 12 -14 days [3]

Branches

If response **Equals '0 days [0]'** then skip to ***phqscore_0 (5.15)***

5.11 phq9b_0

I am concerned about your answer to this last question, and would like to ask you again. Thinking back over the past two weeks, how often have you been bothered by thoughts that you would be better off dead or hurting yourself in some way.

Expects a single option response (**required**)

- ☐ 0 days [0]
- ☐ 1 - 7 days [1]
- ☐ 8 - 11 days [2]
- ☐ 12 -14 days [3]

Branches

If response **Equals '0 days [0]'** then skip to ***phqscore_0 (5.15)***

Prerequisites
Skip when *phq9b_0 (5.11)* Equals '0 days [0]' OR
Skip when *phq9b_0 (5.11)* Equals '8 - 11 days [2]' OR
Skip when *phq9b_0 (5.11)* Equals '12 -14 days [3]'

5.12 SetRefSuicideLeaflet_0

Operator

This field is not displayed on the device, Operator: Set(**ref_suicA_0 (1.5)** ,1)

Prerequisites
Skip when *phq9b_0 (5.11)* Equals '0 days [0]' OR
Skip when *phq9b_0 (5.11)* Equals '1 - 7 days [1]'

5.13 SetRefSuicideLeafletB_0

Operator

This field is not displayed on the device, Operator: Set(**ref_suicB_0 (1.6)** ,1)

Prerequisites
Skip when *phq9b_0 (5.11)* Equals '1 - 7 days [1]'

5.14 SetRefSuicideNurse_0

Operator

This field is not displayed on the device, Operator: Set(**ref_suicA_0 (1.5)** ,1)

5.15 phqscore_0

Numeric

This field is not displayed on the device, Value: 0

5.16 phqscore_set_0

Operator

This field is not displayed on the device, Operator: __{SET(513101,__{SUM(**phq1_0 (5.2)** , **phq2_0 (5.3)** , **phq3_0 (5.4)** , **phq4_0 (5.5)** , **phq5_0 (5.6)** , **phq6_0 (5.7)** , **phq7_0 (5.8)** , **phq8_0 (5.9)** , **phq9a_0 (5.10)**)}__)}__

Prerequisites
Skip when *phq9a_0 (5.10)* Equals '0 days [0]'

5.17 phqscore_setb_0

Operator

This field is not displayed on the device, Operator: __{SET(513101,__{SUM(**phq1_0 (5.2)** , **phq2_0 (5.3)** , **phq3_0 (5.4)** , **phq4_0 (5.5)** , **phq5_0 (5.6)** , **phq6_0 (5.7)** , **phq7_0 (5.8)** , **phq8_0 (5.9)** , **phq9b_0 (5.11)**)}__)}__

Prerequisites
Skip when *phq1_0 (5.2)* Equals '0 days [0]' AND
Skip when *phq2_0 (5.3)* Equals '0 days [0]' AND
Skip when *phq3_0 (5.4)* Equals '0 days [0]' AND
Skip when *phq4_0 (5.5)* Equals '0 days [0]' AND
Skip when *phq5_0 (5.6)* Equals '0 days [0]' AND
Skip when *phq7_0 (5.8)* Equals '0 days [0]' AND
Skip when *phq8_0 (5.9)* Equals '0 days [0]' AND
Skip when *phq9a_0 (5.10)* Equals '0 days [0]'

5.18 phq10_0

If you checked off any problems, how difficult have these problems made it for you to do your work, take care of things at home, or get along with other people?

Expects a single option response (**required**)

☐ not difficult at all [0]

☐ somewhat difficult [1]

☐ very difficult [2]

☐ extremely difficult [3]

5.19 **rx_hpt_0**

Are you currently taking medicine for high blood pressure (hypertension or high blood)?

Expects a single option response (**required**)

☐ Yes [1]

☐ No [0]

5.20 **rx_hiv_0**

Are you currently taking ARVs (antiretrovirals)?

Expects a single option response (**required**)

☐ Yes [1]

☐ No [0]

Prerequisites
Skip when ***rx_hpt_0 (5.19)*** **Equals 'No [0]'** OR
Skip when ***rx_hiv_0 (5.20)*** **Equals 'No [0]'** OR
Skip when ***phqscore_0 (5.15)*** **Less Than '9'**

5.21 **seteft_both_0**

Operator

This field is not displayed on the device, Operator: Set(**eft_0 (1.16)** ,3)

Prerequisites
Skip when ***rx_hpt_0 (5.19)*** **Equals 'No [0]'** OR
Skip when ***rx_hiv_0 (5.20)*** **Equals 'No [0]'** OR
Skip when ***phqscore_0 (5.15)*** **Less Than '9'**

5.22 **cobaltandprime_elig_0**

You qualify for both studies (COBALT and PRIME) and we would like you to take part. I will go through the written information with you and if you would like to take part then we will ask you to sign the form saying you are willing to participate. I will then go through the questionnaire with you, check your measurements (blood pressure, weight, height and waist circumference), and we may want to take a blood sample. [Select PROCEED to continue.]

Expects a single option response (**required**)

☐ Proceed [1]

Branches

If response **Equals 'Proceed [1]'** then skip to ***consent_instr_0 (6.1)***

Prerequisites
Skip when ***rx_hpt_0 (5.19)*** **Equals 'Yes [1]'** OR
Skip when ***phqscore_0 (5.15)*** **Less Than '9'**

5.23 **seteft_cobalt_0**

Operator

This field is not displayed on the device, Operator: Set(**eft_0 (1.16)** ,2)

Prerequisites
Skip when ***rx_hiv_0 (5.20)*** **Equals 'Yes [1]'** OR
Skip when ***phqscore_0 (5.15)*** **Less Than '9'**

5.24 **seteft_prime_0**

Operator

This field is not displayed on the device, Operator: Set(**eft_0 (1.16)** ,1)

Prerequisites
Skip when *phqscore_0 (5.15)* **Less Than '9'** OR
Skip when *rx_hpt_0 (5.19)* **Equals 'Yes [1]'** OR
Skip when *rx_hiv_0 (5.20)* **Equals 'No [0]'**

5.25 cobaltonly_elig_0

You qualify for one of our studies (COBALT) and we would like you to take part. I will go through the written information with you and if you would like to take part then we will ask you to sign the form saying you are willing to participate. I will then go through the questionnaire with you, check your measurements (blood pressure, weight, height and waist circumference), and we may want to take a blood sample. [Select PROCEED to continue.]

Expects a single option response (**required**)

☐ Proceed [1]

Branches

If response **Equals 'Proceed [1]'** then skip to ***consent_instr_0 (6.1)***

Prerequisites
Skip when *phqscore_0 (5.15)* **Less Than '9'** OR
Skip when *rx_hiv_0 (5.20)* **Equals 'Yes [1]'** OR
Skip when *rx_hpt_0 (5.19)* **Equals 'No [0]'**

5.26 primeonly_elig_0

You qualify for one of our studies (PRIME) and we would like you to take part. I will go through the written information with you and if you would like to take part then we will ask you to sign the form saying you are willing to participate. I will then go through the questionnaire with you, check your measurements (blood pressure, weight, height and waist circumference). [Select PROCEED to continue.]

Expects a single option response (**required**)

☐ Proceed [1]

Branches

If response **Equals 'Proceed [1]'** then skip to ***consent_instr_0 (6.1)***

Prerequisites
Skip when *phqscore_0 (5.15)* **Less Than '9'** OR
Skip when *rx_hpt_0 (5.19)* **Equals 'Yes [1]'** OR
Skip when *rx_hiv_0 (5.20)* **Equals 'Yes [1]'**

5.27 seteft_neither_0

Operator

This field is not displayed on the device, Operator: Set(**eft_0 (1.16)** ,0)

5.28 not_elig_0

We are looking for people with certain criteria or illnesses to take part in the study. You do not have the criteria we are looking for so we will not be able to include you in the study. Your usual care will not be affected by not taking part in our study. We would like to thank you very much for answering the questions and for your time today and we wish you well. [Select PROCEED to end the survey.]

Expects a single option response (**required**)

☐ Proceed [1]

Branches

If response **Equals 'Proceed [1]'** then skip to ***end_0 (15.10)***

Section 6. Patient consent

6.1 consent_instr_0

[Now go through patient information sheet and consent form.]

6.2 consent_read_0

[Please confirm that info has been read: must be confirmed in order to continue with the questionnaire]

Expects a single option response (**required**)

- ☐ not confirmed [0]
- ☐ confirmed [1]

Branches

If response **Equals 'not confirmed [0]'** then skip to ***end_0 (15.10)***

6.3 consent_understood_0

[Please confirm that info and consent has been understood and that the participant has the capacity to consent: must be confirmed in order to continue with the questionnaire.]

Expects a single option response (**required**)

- ☐ not confirmed [0]
- ☐ confirmed [1]

Branches

If response **Equals 'not confirmed [0]'** then skip to ***end_0 (15.10)***

6.4 consent_willing_0

Are you still willing to take part in the study/studies?

Expects a single option response (**required**)

- ☐ Yes [1]
- ☐ No [0]

Prerequisites

Skip when ***consent_willing_0 (6.4)*** **Equals 'Yes [1]'**

6.5 consent_not_willing_0

[Please read the following:] We would like to thank you for your time today and we wish you well. [Select PROCEED to continue to the end of the survey.]

Expects a single option response (**required**)

- ☐ Proceed [1]

Branches

If response **Equals 'Proceed [1]'** then skip to ***end_00 (15.11)***

6.6 consent_signed_0

[Please confirm that 2 copies of consent form have been signed: must be confirmed in order to continue with the questionnaire.]

Expects a single option response (**required**)

- ☐ not confirmed [0]
- ☐ confirmed [1]

Branches

If response **Equals 'not confirmed [0]'** then skip to ***end_0 (15.10)***

Section 7. Questionnaire

7.1 name_first_0

I am now going to go through the questionnaire with you. Before we begin, please tell me what is your full English/Afrikaans first name(s)? E.g. John Peter

Expects a single line text response (optional)

7.2 name_surset_0

Please tell me what is your Setswana/Sotho/Zulu first name you might use? E.g. Mpumelelo

Expects a single line text response (optional)

7.3 name_sureng_0

Please tell me what is your surname?

Expects a single line text response (**required**)

7.4 name_surmai_0

Please tell me what is your maiden name?

Expects a single line text response (optional)

7.5 name_init_0

What are your initials? E.g. Joe John Soap = JJ

Expects a single line text response (**required**)

7.6 phone_cell_0

Please could you provide your cell phone number in case we need to contact you?

Expects a phone number (optional)

Constraints

*Response must be **Match '^[0-9]{10}$'***

7.7 sex_0

[Enter sex]

Expects a single option response (**required**)

☐ female [1]

☐ male [0]

7.8 study_id_0

[Please generate and write down Study ID on all relevant documents] MENUACTION(CUSTOMWEB,GENERATE SID)

7.9 bp_instr_0

We will need to take 3 blood pressure readings during the course of the interview, at least 2 minutes apart.

7.10 sbp1_0

[Systolic blood pressure first reading]

Expects a numeric response (**required**)

Prerequisites
Skip when *sbp1_0 (7.10)* **Less Than '201'**

7.11 sbp1_high_0

[You have entered an unusually high number. Please confirm or correct it.]

Expects a single option response (**required**)

- ☐ Confirmed [0]
- ☐ Correct it [1]

Branches

If response **Equals 'Correct it [1]'** then skip to *sbp1_0 (7.10)*

Prerequisites
Skip when *sbp1_0 (7.10)* **Greater Than '89'**

7.12 sbp1_low_confirm

[You have entered an unusually low number: please confirm or correct it.]

Expects a single option response (**required**)

- ☐ Confirmed [0]
- ☐ Correct it [1]

Branches

If response **Equals 'Correct it [1]'** then skip to *sbp1_0 (7.10)*

7.13 dbp1_0

[Diastolic blood pressure first reading]

Expects a numeric response (**required**)

Branches

If response **Less Than 'sbp1_0 (7.10)'** then skip to *his_instr_0 (7.15)*

7.14 dbp1moresbp1_0

The systolic blood pressure must be larger than the diastolic blood pressure. [Please correct the blood pressure readings.]

Expects a single option response (optional)

- ☐ Correct it [0]

Branches

If response **Equals 'Correct it [0]'** then skip to *sbp1_0 (7.10)*

7.15 his_instr_0

I would like to start by asking you a few questions about your past illnesses: has a doctor or nurse ever told you that you have or have had any of the following:

7.16 his_hpt_0

Has a doctor or nurse ever told you that you have or have had high blood pressure (hypertension):

Expects a single option response (**required**)

- ☐ No [0]
- ☐ Yes [1]

7.17 his_mi_0

Has a doctor or nurse ever told you that you have or have had a heart attack:

Expects a single option response (**required**)

☐ No [0]

☐ Yes [1]

7.18 his_cva_0

Has a doctor or nurse ever told you that you have or have had a stroke:

Expects a single option response (**required**)

☐ No [0]

☐ Yes [1]

7.19 his_ang_0

Has a doctor or nurse ever told you that you have or have had angina (chest pains with exertion/activity):

Expects a single option response (**required**)

☐ No [0]

☐ Yes [1]

7.20 his_dep_0

Has a doctor or nurse ever told you that you have or have had depression:

Expects a single option response (**required**)

☐ No [0]

☐ Yes [1]

7.21 his_tb_0

Has a doctor or nurse ever told you that you have or have had TB:

Expects a single option response (**required**)

☐ No [0]

☐ Yes [1]

7.22 his_dm_0

Has a doctor or nurse ever told you that you have or have had diabetes (sugar):

Expects a single option response (**required**)

☐ No [0]

☐ Yes [1]

Prerequisites
Skip when *sex_0 (7.7)* **Equals 'male [0]'**

7.23 his_preg_0

Are you pregnant at the moment?

Expects a single option response (**required**)

☐ No [0]

☐ Yes [1]

Prerequisites
Skip when *sex_0 (7.7)* **Equals 'male [0]'**

7.24 his_baby_0

Have you had a baby in the last year?

Expects a single option response (**required**)

☐ No [0]

☐ Yes [1]

7.25 rx_dep_instr_0

We would like to ask some questions about treatment you receive: Are you currently taking any of the following medications for depression:

7.26 rx_dep_ami_0

Amitriptyline (Tryptanol, Trepiline)

Expects a single option response (**required**)

☐ No [0]

☐ Yes [1]

☐ Don't know [2]

Branches

If response **Equals 'No [0]'** then skip to *rx_dep_flu_0 (7.31)*

If response **Equals 'Don't know [2]'** then skip to *rx_dep_flu_0 (7.31)*

7.27 rx_dep_ami_d_0

Amitriptyline (Tryptanol, Trepiline): dose

Expects a single option response (optional)

☐ 10mg [10]

☐ 25mg [25]

☐ 50mg [50]

☐ 75mg [75]

☐ 100mg [100]

☐ 125mg [125]

☐ 150mg [150]

☐ 175mg [175]

☐ 200mg [200]

☐ 225mg [225]

☐ 250mg [250]

☐ 275mg [275]

☐ 300mg [300]

☐ Other, please specify [0]

Prerequisites
Skip when *rx_dep_ami_d_0 (7.27)* **Not Equal 'Other, please specify [0]'**

7.28 rx_dep_ami_d_other_0

Amitriptyline (Tryptanol, Trepiline): dose other (please specify):

Expects a single line text response (optional)

7.29 **rx_dep_ami_f_0**

Amitriptyline (Tryptanol, Trepiline): number of times per day

Expects a single option response (optional)

- ☐ 1 [1]
- ☐ 2 [2]
- ☐ 3 [3]
- ☐ 4 [4]
- ☐ other [0]

Prerequisites
Skip when *rx_dep_ami_f_0 (7.29)* **Not Equal 'other [0]'**

7.30 **rx_dep_ami_f_other_0**

Amitriptyline (Tryptanol, Trepiline): number of times per day, other (please specify):

Expects a numeric response (optional)

7.31 **rx_dep_flu_0**

Fluoxetine (Prozac, Nuzak, Lorien)

Expects a single option response (**required**)

- ☐ No [0]
- ☐ Yes [1]
- ☐ Don't know [2]

Branches

If response **Equals 'No [0]'** then skip to *rx_dep_cit_0 (7.36)*

If response **Equals 'Don't know [2]'** then skip to *rx_dep_cit_0 (7.36)*

7.32 **rx_dep_flu_d_0**

Fluoxetine (Prozac, Nuzak, Lorien): dose

Expects a single option response (optional)

- ☐ 20mg [20]
- ☐ 40mg [40]
- ☐ 60mg [60]
- ☐ other [0]

Prerequisites
Skip when *rx_dep_flu_d_0 (7.32)* **Not Equal 'other [0]'**

7.33 **rx_dep_flu_d_other_0**

Fluoxetine (Prozac, Nuzak, Lorien): dose other (please specify):

Expects a single line text response (optional)

7.34 **rx_dep_flu_f_0**

Fluoxetine (Prozac, Nuzak, Lorien): number of times per day

Expects a single option response (optional)

- ☐ 1 [1]
- ☐ 2 [2]
- ☐ 3 [3]
- ☐ 4 [4]
- ☐ other [0]

Prerequisites
Skip when *rx_dep_flu_f_0 (7.34)* **Not Equal 'other [0]'**

7.35 rx_dep_flu_f_other_0

Fluoxetine (Prozac, Nuzak, Lorien): number of times per day, other (please specify):

Expects a numeric response (optional)

7.36 rx_dep_cit_0

Citalopram (Cipramil, Cilift)

Expects a single option response (**required**)

- ☐ No [0]
- ☐ Yes [1]
- ☐ Don't know [2]

Branches
If response **Equals 'No [0]'** then skip to *rx_dep_imi_0 (7.41)*
If response **Equals 'Don't know [2]'** then skip to *rx_dep_imi_0 (7.41)*

7.37 rx_dep_cit_d_0

Citalopram (Cipramil, Cilift): dose

Expects a single option response (optional)

- ☐ 20mg [20]
- ☐ 40mg [40]
- ☐ other [0]

Prerequisites
Skip when *rx_dep_cit_d_0 (7.37)* **Not Equal 'other [0]'**

7.38 rx_dep_cit_d_other_0

Citalopram (Cipramil, Cilift): dose, other (please specify):

Expects a single line text response (optional)

7.39 rx_dep_cit_f_0

Citalopram (Cipramil, Cilift): number of times per day

Expects a single option response (optional)

- ☐ 1 [1]
- ☐ 2 [2]
- ☐ 3 [3]
- ☐ 4 [4]
- ☐ other [0]

Prerequisites
Skip when *rx_dep_cit_f_0 (7.39)* **Not Equal 'other [0]'**

7.40 rx_dep_cit_f_other_0

Citalopram (Cipramil, Cilift): number of times per day, other (please specify):

Expects a numeric response (optional)

7.41 rx_dep_imi_0

Imipramine (Tofranil)

Expects a single option response (**required**)

- ☐ No [0]
- ☐ Yes [1]
- ☐ Don't know [2]

Branches

If response **Equals 'No [0]'** then skip to *rx_dep_clo_0 (7.46)*

If response **Equals 'Don't know [2]'** then skip to *rx_dep_clo_0 (7.46)*

7.42 rx_dep_imi_d_0

Imipramine (Tofranil): dose

Expects a single option response (**required**)

- ☐ 10mg [10]
- ☐ 25mg [25]
- ☐ 50mg [50]
- ☐ 75mg [75]
- ☐ 100mg [100]
- ☐ 125mg [125]
- ☐ 150mg [150]
- ☐ 175mg [175]
- ☐ 200mg [200]
- ☐ 225mg [225]
- ☐ 250mg [250]
- ☐ 275mg [275]
- ☐ 300mg [300]
- ☐ other [0]

Prerequisites
Skip when *rx_dep_imi_d_0 (7.42)* **Not Equal 'other [0]'**

7.43 rx_dep_imi_d_other_0

Imipramine (Tofranil): dose, other (please specify):

Expects a single line text response (optional)

7.44 rx_dep_imi_f_0

Imipramine (Tofranil): number of times per day

Expects a single option response (**required**)

- ☐ 1 [1]
- ☐ 2 [2]
- ☐ 3 [3]
- ☐ 4 [4]
- ☐ other [0]

Prerequisites
Skip when *rx_dep_imi_f_0 (7.44)* **Not Equal 'other [0]'**

7.45 rx_dep_imi_f_other_0

Imipramine (Tofranil): number of times per day, other (please specify):

Expects a numeric response (optional)

7.46 rx_dep_clo_0

Clomipramine (Anafranil)

Expects a single option response (**required**)

- ☐ No [0]
- ☐ Yes [1]
- ☐ Don't know [2]

Branches

If response **Equals 'No [0]'** then skip to ***rx_dep_dot_0 (7.51)***

If response **Equals 'Don't know [2]'** then skip to ***rx_dep_dot_0 (7.51)***

7.47 rx_dep_clo_d_0

Clomipramine (Anafranil): dose

Expects a single option response (**required**)

- ☐ 10mg [10]
- ☐ 25mg [25]
- ☐ 50mg [50]
- ☐ 75mg [75]
- ☐ 100mg [100]
- ☐ 125mg [125]
- ☐ 150mg [150]
- ☐ other [0]

Prerequisites

Skip when ***rx_dep_clo_d_0 (7.47)*** **Not Equal 'other [0]'**

7.48 rx_dep_clo_d_other_0

Clomipramine (Anafranil): dose, other (please specify):

Expects a single line text response (optional)

7.49 rx_dep_clo_f_0

Clomipramine (Anafranil): number of times per day

Expects a single option response (**required**)

- ☐ 1 [1]
- ☐ 2 [2]
- ☐ 3 [3]
- ☐ 4 [4]
- ☐ other [0]

Prerequisites

Skip when ***rx_dep_clo_f_0 (7.49)*** **Not Equal 'other [0]'**

7.50 rx_dep_clo_f_other_0

Clomipramine (Anafranil): number of times per day, other (please specify):

Expects a numeric response (optional)

7.51 rx_dep_dot_0

Dothiepin (Prothiaden, Thaden)

Expects a single option response (**required**)

☐ No [0]

☐ Yes [1]

☐ Don't know [2]

Branches

If response **Equals 'No [0]'** then skip to ***rx_dep_mia_0 (7.56)***

If response **Equals 'Don't know [2]'** then skip to ***rx_dep_mia_0 (7.56)***

7.52 rx_dep_dot_d_0

Dothiepin (Prothiaden, Thaden): dose

Expects a single option response (**required**)

☐ 10mg [10]

☐ 25mg [25]

☐ 50mg [50]

☐ 75mg [75]

☐ 100mg [100]

☐ 125mg [125]

☐ 150mg [150]

☐ other [0]

Prerequisites
Skip when ***rx_dep_dot_d_0 (7.52)*** **Not Equal 'other [0]'**

7.53 rx_dep_dot_d_other_0

Dothiepin (Prothiaden, Thaden): dose, other (please specify):

Expects a single line text response (optional)

7.54 rx_dep_dot_f_0

Dothiepin (Prothiaden, Thaden): number of times per day

Expects a single option response (**required**)

☐ 1 [1]

☐ 2 [2]

☐ 3 [3]

☐ 4 [4]

☐ other [0]

Prerequisites
Skip when ***rx_dep_dot_f_0 (7.54)*** **Not Equal 'other [0]'**

7.55 rx_dep_dot_f_other_0

Dothiepin (Prothiaden, Thaden): number of times per day, other (please specify):

Expects a numeric response (optional)

7.56 rx_dep_mia_0

Mianserin (Lantanon)

Expects a single option response (**required**)

- ☐ No [0]
- ☐ Yes [1]
- ☐ Don't know [2]

Branches

If response **Equals 'No [0]'** then skip to ***rx_dep_ven_0 (7.61)***

If response **Equals 'Don't know [2]'** then skip to ***rx_dep_ven_0 (7.61)***

7.57 rx_dep_mia_d_0

Mianserin (Lantanon): dose

Expects a single option response (**required**)

- ☐ 10mg [10]
- ☐ 30mg [30]
- ☐ 60mg [60]
- ☐ other [0]

Prerequisites
Skip when ***rx_dep_mia_d_0 (7.57)*** **Not Equal 'other [0]'**

7.58 rx_dep_mia_d_other_0

Mianserin (Lantanon): dose other (please specify):

Expects a single line text response (optional)

7.59 rx_dep_mia_f_0

Mianserin (Lantanon): number of times per day

Expects a single option response (**required**)

- ☐ 1 [1]
- ☐ 2 [2]
- ☐ 3 [3]
- ☐ 4 [4]
- ☐ other [0]

Prerequisites
Skip when ***rx_dep_mia_f_0 (7.59)*** **Not Equal 'other [0]'**

7.60 rx_dep_mia_f_other_0

Mianserin (Lantanon): number of times per day, other (please specify):

Expects a numeric response (optional)

7.61 rx_dep_ven_0

Venlafaxine (Efexor, Venlor)

Expects a single option response (**required**)

- ☐ No [0]
- ☐ Yes [1]
- ☐ Don't know [2]

Branches

If response **Equals 'No [0]'** then skip to ***rx_dep_oth_0 (7.66)***

If response **Equals 'Don't know [2]'** then skip to ***rx_dep_oth_0 (7.66)***

7.62 rx_dep_ven_d_0

Venlafaxine (Efexor, Venlor): dose

Expects a single option response (**required**)

- ☐ 37.5mg [37.5]
- ☐ 75mg [75]
- ☐ 150mg [150]
- ☐ 225mg [225]
- ☐ 300mg [300]
- ☐ 375mg [375]
- ☐ other [0]

Prerequisites
Skip when ***rx_dep_ven_d_0 (7.62)*** **Not Equal 'other [0]'**

7.63 rx_dep_ven_d_other_0

Venlafaxine (Efexor, Venlor): dose, other (please specify):

Expects a single line text response (**required**)

7.64 rx_dep_ven_f_0

Venlafaxine (Efexor, Venlor): number of times per day

Expects a single option response (**required**)

- ☐ 1 [1]
- ☐ 2 [2]
- ☐ 3 [3]
- ☐ 4 [4]
- ☐ other [0]

Prerequisites
Skip when ***rx_dep_ven_f_0 (7.64)*** **Not Equal 'other [0]'**

7.65 rx_dep_ven_f_other_0

Venlafaxine (Efexor, Venlor): number of times per day, other (please specify):

Expects a numeric response (optional)

7.66 rx_dep_oth_0

Other medication for depression: please specify name

Expects a single line text response (optional)

7.67 rx_dep_oth_d_0

Other medication for depression: please specify dose

Expects a single line text response (optional)

7.68 rx_dep_oth_f_0

Other medication for depression: please specify number of times per day

Expects a numeric response (optional)

Section 8. Counselling

8.1 rx_dep_cou_instr_0

We would like to know whether you have received counselling in the last 3 months specifically for your depression. Counselling is not just receiving advice on how to take medication. It means talking with someone in a way that helps you to find solutions to your problems, or receive emotional support.

8.2 rx_dep_cou_qread_0

[Please confirm that the counselling question has been read in full]

Expects a single option response (**required**)

☐ No [0]

☐ Yes [1]

Branches

If response **Equals 'No [0]'** then skip to ***rx_dep_cou_instr_0 (8.1)***

8.3 rx_dep_cou_qund_0

Do you understand what we mean by counselling?

Expects a single option response (**required**)

☐ No [0]

☐ Yes [1]

Branches

If response **Equals 'No [0]'** then skip to ***rx_dep_cou_instr_0 (8.1)***

8.4 rx_dep_cou_instr2_0

Have you received counselling from any of the following people in the last 3 months specifically for your depression:

8.5 rx_dep_cou_n_0

Have you received counselling from any of the following people in the last 3 months specifically for your depression: Nurse

Expects a single option response (**required**)

☐ No [0]

☐ Yes [1]

8.6 rx_dep_cou_cc_0

Have you received counselling from any of the following people in the last 3 months specifically for your depression: Clinic counsellor (whether on a one-to-one basis or in a group)

Expects a single option response (**required**)

☐ No [0]

☐ Yes [1]

8.7 rx_dep_cou_s_0

Have you received counselling from any of the following people in the last 3 months specifically for your depression: Social worker

Expects a single option response (**required**)

☐ No [0]

☐ Yes [1]

8.8 rx_dep_cou_po_0

Have you received counselling from any of the following people in the last 3 months specifically for your depression: Psychologist

Expects a single option response (**required**)

☐ No [0]

☐ Yes [1]

8.9 rx_dep_cou_pi_0

Have you received counselling from any of the following people in the last 3 months specifically for your depression: Psychiatrist (a doctor specialising in mental health)

Expects a single option response (**required**)

☐ No [0]

☐ Yes [1]

8.10 rx_dep_cou_d_0

Have you received counselling from any of the following people in the last 3 months specifically for your depression: Doctor (other than a psychiatrist)

Expects a single option response (**required**)

☐ No [0]

☐ Yes [1]

8.11 rx_dep_cou_t_0

Have you received counselling from any of the following people in the last 3 months specifically for your depression: Traditional healer

Expects a single option response (**required**)

☐ No [0]

☐ Yes [1]

8.12 rx_dep_cou_r_0

Have you received counselling from any of the following people in the last 3 months specifically for your depression: Religious or spiritual advisor

Expects a single option response (**required**)

☐ No [0]

☐ Yes [1]

8.13 rx_dep_cou_ccw_0

Have you received counselling from any of the following people in the last 3 months specifically for your depression: Community health worker (CCW)

Expects a single option response (**required**)

☐ No [0]

☐ Yes [1]

8.14 rx_dep_cou_o_0

Have you received counselling from any of the following people in the last 3 months specifically for your depression: Other

Expects a single option response (**required**)

☐ No [0]

☐ Yes [1]

Prerequisites
Skip when *rx_dep_cou_o_0 (8.14)* **Equals 'No [0]'**

8.15 rx_dep_cou_ospec_0

Please specify

Expects a single line text response (**required**)

Prerequisites
Skip when *rx_hiv_0 (5.20)* **Equals 'No [0]'**

8.16 rx_hiv_instr_0

You mentioned earlier you are on ARVs. Please specify which ARVs you are currently taking:

Prerequisites
Skip when *rx_hiv_0 (5.20)* **Equals 'No [0]'**

8.17 rx_hiv_fdc_0

Please specify which ARVs you are currently taking: FDC (one pill once a day)

Expects a single option response (**required**)

☐ No [0]

☐ Yes [1]

Branches

If response **Equals 'Yes [1]'** then skip to *rx_hiv_startdate_0 (8.25)*

Prerequisites
Skip when *rx_hiv_0 (5.20)* **Equals 'No [0]'**

8.18 rx_hiv_ten_0

Please specify which ARVs you are currently taking: Tenofovir

Expects a single option response (**required**)

☐ No [0]

☐ Yes [1]

Prerequisites
Skip when *rx_hiv_0 (5.20)* **Equals 'No [0]'**

8.19 rx_hiv_lam_0

Please specify which ARVs you are currently taking: Lamivudine

Expects a single option response (**required**)

☐ No [0]

☐ Yes [1]

Prerequisites
Skip when *rx_hiv_0 (5.20)* **Equals 'No [0]'**

8.20 rx_hiv_stav_0

Please specify which ARVs you are currently taking: Stavudine

Expects a single option response (**required**)

☐ No [0]

☐ Yes [1]

Prerequisites
Skip when *rx_hiv_0 (5.20)* **Equals 'No [0]'**

8.21 rx_hiv_efa_0

Please specify which ARVs you are currently taking: Efavirenz

Expects a single option response (**required**)

☐ No [0]

☐ Yes [1]

Prerequisites
Skip when *rx_hiv_0 (5.20)* **Equals 'No [0]'**

8.22 rx_hiv_zid_0

Please specify which ARVs you are currently taking: Zidovudine

Expects a single option response (**required**)

☐ No [0]

☐ Yes [1]

Prerequisites
Skip when *rx_hiv_0 (5.20)* **Equals 'No [0]'**

8.23 rx_hiv_nev_0

Please specify which ARVs you are currently taking: Nevirapine

Expects a single option response (**required**)

☐ No [0]

☐ Yes [1]

Prerequisites
Skip when *rx_hiv_0 (5.20)* **Equals 'No [0]'**

8.24 rx_hiv_oth_0

Please specify which ARVs you are currently taking: Other: please specify

Expects a single line text response (optional)

Prerequisites
Skip when *rx_hiv_0 (5.20)* **Equals 'No [0]'**

8.25 rx_hiv_startdate_0

When did you first start taking ARVs?

Expects a single option response (**required**)

☐ Exact date [0]

☐ Do not know [1]

☐ Month and year known [2]

Branches
If response **Equals 'Do not know [1]'** then skip to ***adh_vas_0 (8.29)***

Prerequisites
Skip when *rx_hiv_0 (5.20)* **Equals 'No [0]'** OR
Skip when *rx_hiv_startdate_0 (8.25)* **Not Equal 'Exact date [0]'**

8.26 rx_hiv_exactstartdate_0

Enter date:

Expects a date response (**required**)

Prerequisites
Skip when *rx_hiv_0 (5.20)* **Equals 'No [0]'** OR
Skip when *rx_hiv_startdate_0 (8.25)* **Not Equal 'Month and year known [2]'**

8.27 rx_hiv_startdateyear_0

Enter year:

Expects a numeric response (**required**)

Prerequisites
Skip when *rx_hiv_0 (5.20)* **Equals 'No [0]'** OR
Skip when *rx_hiv_startdate_0 (8.25)* **Not Equal 'Month and year known [2]'**

8.28 rx_hiv_startdatemonth_0

Enter month:

Expects a numeric response (**required**)

Constraints

Response must be ***Less Than or Equal '12'***

Prerequisites
Skip when *rx_hiv_0 (5.20)* **Equals 'No [0]'**

8.29 adh_vas_0

We would be surprised if most people take 100% of their medications. 0% means you have taken no ARVs this past month, 50% means you have taken half of your ARVs this past month, and 100% means you have taken every single dose this past month. What percentage of your ARVs did you take?

Expects a numeric response (**required**)

Constraints

Response must be ***Greater Than or Equal '0'*** AND

Response must be ***Less Than or Equal '100'***

8.30 clinicfolder_available_0

[Is the patient's clinic folder available?]

Expects a single option response (**required**)

☐ No [0]

☐ Yes [1]

Prerequisites
Skip when *rx_hiv_0 (5.20)* **Equals 'No [0]'** OR
Skip when *clinicfolder_available_0 (8.30)* **Equals 'No [0]'**

8.31 vl_date_0

[Please look through the patient's clinic folder now and document the last viral load blood test (date of test and value)]

Expects a single option response (**required**)

☐ Available [0]

☐ Unknown [1]

Prerequisites
Skip when *rx_hiv_0 (5.20)* **Equals 'No [0]'** OR
Skip when *vl_date_0 (8.31)* **Not Equal 'Available [0]'** OR
Skip when *clinicfolder_available_0 (8.30)* **Equals 'No [0]'**

8.32 vl_date_start_0

Enter date:

Expects a date response (optional)

Constraints

Response must be ***Less Than or Equal 'DATENOW'***

Prerequisites
Skip when *rx_hiv_0 (5.20)* **Equals 'No [0]'** OR
Skip when *vl_date_0 (8.31)* **Equals 'Unknown [1]'** OR
Skip when *clinicfolder_available_0 (8.30)* **Equals 'No [0]'**

8.33 CalcVLReference

Numeric

This field is not displayed on the device, Value: Set(**CalculatedVLReference (1.7)** , ROUND(DIVISION OF (DAYSBETWEEN(Q513605, DATENOW) ,30) ,2))

Prerequisites
Skip when *rx_hiv_0 (5.20)* **Equals 'No [0]'** OR
Skip when *vl_date_0 (8.31)* **Equals 'Unknown [1]'** OR
Skip when *clinicfolder_available_0 (8.30)* **Equals 'No [0]'**

8.34 DisplayVLReference

Number of months since the last viral load test: **CalculatedVLReference (1.7)**

Prerequisites
Skip when *rx_hiv_0 (5.20)* **Equals 'No [0]'** OR
Skip when *clinicfolder_available_0 (8.30)* **Equals 'No [0]'**

8.35 vl_value_avail_0

Is the viral load DATE and VALUE available?

Expects a single option response (**required**)

☐ No [0]

☐ Yes [1]

Prerequisites
Skip when *rx_hiv_0 (5.20)* **Equals 'No [0]'** OR
Skip when *clinicfolder_available_0 (8.30)* **Equals 'No [0]'** OR
Skip when *vl_value_avail_0 (8.35)* **Equals 'No [0]'**

8.36 vl_value_0

[Please look through the patient's clinic folder now and document the VALUE of the last last viral load blood test.]

Expects a single line text response (optional)

Prerequisites
Skip when *rx_hiv_0 (5.20)* **Equals 'No [0]'** OR
Skip when *clinicfolder_available_0 (8.30)* **Equals 'No [0]'** OR
Skip when *CalculatedVLReference (1.7)* **Less Than '6'**

8.37 set_vl_flag_0

Operator

This field is not displayed on the device, Operator: Set(**ref_vl_0 (1.8)** ,1)

Prerequisites
Skip when *rx_hiv_0 (5.20)* **Equals 'No [0]'** OR
Skip when *clinicfolder_available_0 (8.30)* **Equals 'No [0]'** OR
Skip when *vl_value_avail_0 (8.35)* **Equals 'Yes [1]'**

8.38 set_vl_flag_no_value_0

Operator

This field is not displayed on the device, Operator: Set(**ref_vl_0 (1.8)** ,1)

Prerequisites
Skip when *rx_hiv_0 (5.20)* **Equals 'No [0]'** OR
Skip when *clinicfolder_available_0 (8.30)* **Equals 'Yes [1]'**

8.39 set_vl_ref_no_folder_0

Operator

This field is not displayed on the device, Operator: Set(**ref_vl_0 (1.8)** ,1)

Prerequisites
Skip when *rx_hiv_0 (5.20)* Equals 'No [0]' OR
Skip when *clinicfolder_available_0 (8.30)* Equals 'No [0]'

8.40 vl_date_none_0

[Please confirm that you have looked through the patient's clinic folder.]

Expects multiple selected options (**required**)

☐ Clinic folder checked [1]

☐ No viral load result found [0]

Prerequisites
Skip when *clinicfolder_available_0 (8.30)* Equals 'No [0]'

8.41 ret_date_start_0

[Please look through the patient's clinic folder now and document the most recent date of care (clinic note, prescription date, blood result or other)]

Expects a date response (optional)

Constraints

Response must be *Less Than or Equal 'DATENOW'*

Prerequisites
Skip when *clinicfolder_available_0 (8.30)* Equals 'No [0]'

8.42 ret_reason_0

[Please enter the reason for most recent care.]

Expects multiple selected options (**required**)

☐ Nurse or doctor clinic note [Nurse or doctor clinic note]

☐ Prescription date [Prescription date]

☐ Blood result [Blood result]

☐ Other [Other]

Prerequisites
Skip when *ret_reason_0 (8.42)* Excludes 'Other [Other]' OR
Skip when *clinicfolder_available_0 (8.30)* Equals 'No [0]'

8.43 ret_other_0

If other selected, please specify:

Expects a long text response (optional)

8.44 cig_0

We would like to ask you some questions about smoking: Are you currently smoking cigarettes daily?

Expects a single option response (**required**)

☐ No [0]

☐ Yes [1]

Branches

If response **Equals 'No [0]'** then skip to *bp2_inst_0 (8.53)*

8.45 cig_num_0

How many cigarettes do you smoke each day (on average)?

Expects a numeric response (**required**)

[ ]

Constraints

Response must be ***Greater Than or Equal '0'***

Prerequisites
Skip when ***cig_num_0 (8.45)*** **Less Than '61'**

8.46 cig_no_high_0

[You have entered an unusually high number. Please confirm or correct it.]

Expects a single option response (**required**)

- ☐ Confirmed [0]
- ☐ Correct it [1]

Branches

If response **Equals 'Correct it [1]'** then skip to ***cig_num_0 (8.45)***

Prerequisites
Skip when ***cig_num_0 (8.45)*** **Not Equal '0'**

8.47 cig_num_zero_0

[Please confirm that the number of cigarettes smoked each day = 0 or revise the amount smoked in the previous question.]

Expects a single option response (**required**)

- ☐ Confirmed [0]
- ☐ Correct it [1]

Branches

If response **Equals 'Correct it [1]'** then skip to ***cig_num_0 (8.45)***

8.48 cig_start_0

How old were you when you first started smoking cigarettes?

Expects a numeric response (**required**)

[ ]

Prerequisites
Skip when ***cig_start_0 (8.48)*** **Greater Than '11'**

8.49 cig_start_confirm

[You have entered an unusually low number: please confirm or correct it.]

Expects a single option response (**required**)

- ☐ Confirmed [0]
- ☐ Correct it [1]

Branches

If response **Equals 'Correct it [1]'** then skip to ***cig_start_0 (8.48)***

8.50 cig_quit_0

In the last year, how many times have you quit smoking for at least 24 hours?

Expects a numeric response (**required**)

[ ]

Prerequisites
Skip when *cig_quit_0 (8.50)* Less Than '21'

8.51 cig_quit_confirm

[You have entered an unusually high number: please confirm or correct it.]

Expects a single option response (**required**)

- ☐ Confirmed [0]
- ☐ Correct it [1]

Branches

If response **Equals 'Correct it [1]'** then skip to ***cig_quit_0 (8.50)***

8.52 cig_think_0

Are you seriously thinking of quitting smoking. Choose one of the following options:

Expects a single option response (**required**)

- ☐ Yes, within the next 30 days [0]
- ☐ Yes, within the next 6 months [1]
- ☐ No, not thinking of quitting [2]

8.53 bp2_inst_0

I am now going to take your second blood pressure reading.

8.54 sbp2_0

[Systolic blood pressure, second reading]

Expects a numeric response (**required**)

Prerequisites
Skip when *sbp2_0 (8.54)* Less Than '201'

8.55 sbp2_high_0

[You have entered an unusually high number. Please confirm or correct it.]

Expects a single option response (**required**)

- ☐ Confirmed [0]
- ☐ Correct it [1]

Branches

If response **Equals 'Correct it [1]'** then skip to ***sbp2_0 (8.54)***

Prerequisites
Skip when *sbp2_0 (8.54)* Greater Than '89'

8.56 sbp2_low_confirm

[You have entered an unusually low number: please confirm or correct it.]

Expects a single option response (**required**)

- ☐ Confirmed [0]
- ☐ Correct it [1]

Branches

If response **Equals 'Correct it [1]'** then skip to ***sbp2_0 (8.54)***

8.57 dbp2_0

[Diastolic blood pressure, second reading]

Expects a numeric response (**required**)

Constraints

*Response must be **Less Than or Equal** 'sbp2_0 (8.54)'*

Branches

If response **Less Than** 'sbp2_0 (8.54)' then skip to ***anx_instr_0 (8.59)***

8.58 dbp2moresbp2_0

The systolic blood pressure must be larger than the diastolic blood pressure. [Please correct the blood pressure readings.]

Expects a single option response (**required**)

☐ Correct it [0]

Branches

If response **Equals 'Correct it [0]'** then skip to ***sbp2_0 (8.54)***

8.59 anx_instr_0

The following questions ask you about your feelings and thoughts during the last month. In each case, you will be asked to indicate how often you felt or thought a certain way.

8.60 anx1_0

In the last month, how often have you been upset because of something that happened unexpectedly?

Expects a single option response (**required**)

☐ Never [0]

☐ Almost Never [1]

☐ Sometimes [2]

☐ Fairly Often [3]

☐ Very Often [4]

8.61 anx2_0

In the last month, how often have you felt that you were unable to control the important things in your life?

Expects a single option response (**required**)

☐ Never [0]

☐ Almost Never [1]

☐ Sometimes [2]

☐ Fairly Often [3]

☐ Very Often [4]

8.62 anx3_0

In the last month, how often have you felt nervous and stressed?

Expects a single option response (**required**)

☐ Never [0]

☐ Almost Never [1]

☐ Sometimes [2]

☐ Fairly Often [3]

☐ Very Often [4]

8.63 anx4_0

In the last month, how often have you felt confident about your ability to handle your personal problems?

Expects a single option response (**required**)

- ☐ Never [0]
- ☐ Almost Never [1]
- ☐ Sometimes [2]
- ☐ Fairly Often [3]
- ☐ Very Often [4]

8.64 anx5_0

In the last month, how often have you felt that things were going your way?

Expects a single option response (**required**)

- ☐ Never [0]
- ☐ Almost Never [1]
- ☐ Sometimes [2]
- ☐ Fairly Often [3]
- ☐ Very Often [4]

8.65 anx6_0

In the last month, how often have you found that you could not cope with all the things that you had to do?

Expects a single option response (**required**)

- ☐ Never [0]
- ☐ Almost Never [1]
- ☐ Sometimes [2]
- ☐ Fairly Often [3]
- ☐ Very Often [4]

8.66 anx7_0

In the last month, how often have you been able to control irritations in your life?

Expects a single option response (**required**)

- ☐ Never [0]
- ☐ Almost Never [1]
- ☐ Sometimes [2]
- ☐ Fairly Often [3]
- ☐ Very Often [4]

8.67 anx8_0

In the last month, how often have you felt that you were on top of things?

Expects a single option response (**required**)

- ☐ Never [0]
- ☐ Almost Never [1]
- ☐ Sometimes [2]
- ☐ Fairly Often [3]
- ☐ Very Often [4]

8.68 anx9_0

In the last month, how often have you been angered because of things that were outside of your control?

Expects a single option response (**required**)

- ☐ Never [0]
- ☐ Almost Never [1]
- ☐ Sometimes [2]
- ☐ Fairly Often [3]
- ☐ Very Often [4]

8.69 anx10_0

In the last month, how often have you felt difficulties were piling up so high that you could not overcome them?

Expects a single option response (**required**)

- ☐ Never [0]
- ☐ Almost Never [1]
- ☐ Sometimes [2]
- ☐ Fairly Often [3]
- ☐ Very Often [4]

8.70 whodas_instr_0

The following questions ask about difficulties you may have had in the past month.

8.71 whodas1_0

In the past 30 days, how much difficulty did you have in: Standing for long periods such as 30 minutes?

Expects a single option response (**required**)

- ☐ None [1]
- ☐ Mild [2]
- ☐ Moderate [3]
- ☐ Severe [4]
- ☐ Extreme or cannot do [5]

8.72 whodas2_0

In the past 30 days, how much difficulty did you have in: Taking care of your household responsibilities?

Expects a single option response (**required**)

- ☐ None [1]
- ☐ Mild [2]
- ☐ Moderate [3]
- ☐ Severe [4]
- ☐ Extreme or cannot do [5]

8.73 whodas3_0

In the past 30 days, how much difficulty did you have in: Learning a new task, for example, learning how to get to a new place?

Expects a single option response (**required**)

- ☐ None [1]
- ☐ Mild [2]
- ☐ Moderate [3]
- ☐ Severe [4]
- ☐ Extreme or cannot do [5]

8.74 whodas4_0

In the past 30 days, how much difficulty did you have in: Joining in community activities (for example, festivities, religious or other activities) in the same way as anyone else can?

Expects a single option response (**required**)

- ☐ None [1]
- ☐ Mild [2]
- ☐ Moderate [3]
- ☐ Severe [4]
- ☐ Extreme or cannot do [5]

8.75 whodas5_0

In the past 30 days, how much have you been emotionally affected by your health problems?

Expects a single option response (**required**)

- ☐ None [1]
- ☐ Mild [2]
- ☐ Moderate [3]
- ☐ Severe [4]
- ☐ Extreme or cannot do [5]

8.76 whodas6_0

In the past 30 days, how much difficulty did you have in: Concentrating on doing something for ten minutes?

Expects a single option response (**required**)

- ☐ None [1]
- ☐ Mild [2]
- ☐ Moderate [3]
- ☐ Severe [4]
- ☐ Extreme or cannot do [5]

8.77 whodas7_0

In the past 30 days, how much difficulty did you have in: Walking a long distance such as a kilometre (or equivalent)?

Expects a single option response (**required**)

- ☐ None [1]
- ☐ Mild [2]
- ☐ Moderate [3]
- ☐ Severe [4]
- ☐ Extreme or cannot do [5]

8.78 whodas8_0

In the past 30 days, how much difficulty did you have in: Washing your whole body?

Expects a single option response (**required**)

- ☐ None [1]
- ☐ Mild [2]
- ☐ Moderate [3]
- ☐ Severe [4]
- ☐ Extreme or cannot do [5]

8.79 whodas9_0

In the past 30 days, how much difficulty did you have in: Getting dressed?

Expects a single option response (**required**)

- ☐ None [1]
- ☐ Mild [2]
- ☐ Moderate [3]
- ☐ Severe [4]
- ☐ Extreme or cannot do [5]

8.80 whodas10_0

In the past 30 days, how much difficulty did you have in: Dealing with people you do not know?

Expects a single option response (**required**)

- ☐ None [1]
- ☐ Mild [2]
- ☐ Moderate [3]
- ☐ Severe [4]
- ☐ Extreme or cannot do [5]

8.81 whodas11_0

In the past 30 days, how much difficulty did you have in: Maintaining a friendship?

Expects a single option response (**required**)

- ☐ None [1]
- ☐ Mild [2]
- ☐ Moderate [3]
- ☐ Severe [4]
- ☐ Extreme or cannot do [5]

8.82 whodas12_0

In the past 30 days, how much difficulty did you have in: Your day-to-day work/school?

Expects a single option response (**required**)

- ☐ None [1]
- ☐ Mild [2]
- ☐ Moderate [3]
- ☐ Severe [4]
- ☐ Extreme or cannot do [5]

8.83 whodas13_0

Overall, in the past 30 days, how many days were these difficulties present?

Expects a numeric response (**required**)

Constraints

Response must be ***Greater Than or Equal '0'*** *AND*

Response must be ***Less Than or Equal '30'***

8.84 whodas14_0

In the past 30 days, for how many days were you totally unable to carry out your usual activities or work because of any health condition?

Expects a numeric response (**required**)

Constraints

Response must be ***Greater Than or Equal '0'*** *AND*

Response must be ***Less Than or Equal '30'***

8.85 whodas15_0

In the past 30 days, not counting the days that you were totally unable, for how many days did you cut back or reduce your usual activities or work because of any health condition?

Expects a numeric response (**required**)

Constraints

Response must be ***Greater Than or Equal '0'*** *AND*

Response must be ***Less Than or Equal '30'***

8.86 bp3_instr_0

I am now going to take your third blood pressure reading, height, weight and waist measurements.

8.87 sbp3_0

[Systolic blood pressure, third reading]

Expects a numeric response (**required**)

Prerequisites

Skip when ***sbp3_0 (8.87)*** **Less Than '201'**

8.88 sbp3_high_0

[You have entered an unusually high number. Please confirm or correct it.]

Expects a single option response (**required**)

☐ Confirmed [0]

☐ Correct it [1]

Branches

If response **Equals 'Correct it [1]'** then skip to ***sbp3_0 (8.87)***

Prerequisites
Skip when *sbp3_0 (8.87)* **Greater Than '89'**

8.89 sbp3_low_confirm

[You have entered an unusually low number: please confirm or correct it.]

Expects a single option response (**required**)

- [ ] Confirmed [0]
- [ ] Correct it [1]

Branches

If response **Equals 'Correct it [1]'** then skip to ***sbp3_0 (8.87)***

8.90 dbp3_0

[Diastolic blood pressure, third reading]

Expects a numeric response (**required**)

Branches

If response **Less Than** '**sbp3_0 (8.87)**' then skip to ***mean_sbp_0 (8.92)***

8.91 dbp3moresbp3_0

The systolic blood pressure must be larger than the diastolic blood pressure. [Please correct the blood pressure readings.]

Expects a single option response (**required**)

- [ ] Correct it [0]

Branches

If response **Equals 'Correct it [0]'** then skip to ***sbp3_0 (8.87)***

8.92 mean_sbp_0

Numeric

This field is not displayed on the device, Value: 0

8.93 set_meandbp_0

Numeric

This field is not displayed on the device, Value: 0

8.94 set_mean_sbp_0

Operator

This field is not displayed on the device, Operator: __{SET(513995,__{AVG(**sbp2_0 (8.54)** , **sbp3_0 (8.87)**)}__)}__

8.95 set_mean_dbp_0

Operator

This field is not displayed on the device, Operator: __{SET(513996,__{AVG(**dbp2_0 (8.57)** , **dbp3_0 (8.90)**)}__)}__

Prerequisites
Skip when ***meansbp_0 (1.3)*** **Less Than '180'** AND
Skip when ***meandbp_0 (1.4)*** **Less Than '110'**

8.96 set_bp_flag_0

Operator

This field is not displayed on the device, Operator: Set(**ref_bp_0 (1.9)** ,1)

8.97 height_0

[Enter height in cm to one decimal place]

Expects a decimal response (**required**)

Constraints

*Response must be **Greater Than '0'***

Prerequisites
Skip when ***height_0 (8.97)*** **Less Than '186'**

8.98 height_high_0

[You have entered an unusually high number. Please confirm or correct it.]

Expects a single option response (**required**)

- ☐ Confirmed [0]
- ☐ Correct it [1]

Branches

If response **Equals 'Correct it [1]'** then skip to ***height_0 (8.97)***

Prerequisites
Skip when ***height_0 (8.97)*** **Greater Than '144'**

8.99 height_low_confirm

[You have entered an unusually low number: please confirm or correct it.]

Expects a single option response (**required**)

- ☐ Confirmed [0]
- ☐ Correct it [1]

Branches

If response **Equals 'Correct it [1]'** then skip to ***height_0 (8.97)***

8.100 weight_0

[Enter weight in kg to one decimal place]

Expects a decimal response (**required**)

Constraints

*Response must be **Greater Than '0'***

Prerequisites
Skip when ***weight_0 (8.100)*** **Less Than '136'**

8.101 weight_high_0

[You have entered an unusually high number. Please confirm or correct it.]

Expects a single option response (**required**)

- ☐ Confirmed [0]
- ☐ Correct it [1]

Branches

If response **Equals 'Correct it [1]'** then skip to ***weight_0 (8.100)***

Prerequisites
Skip when *weight_0 (8.100)* **Greater Than '49'**

8.102 weight_low_confirm

[You have entered an unusually low number: please confirm or correct it.]

Expects a single option response (**required**)

- ☐ Confirmed [0]
- ☐ Correct it [1]

Branches

If response **Equals 'Correct it [1]'** then skip to ***weight_0 (8.100)***

8.103 height_m_0

Numeric

This field is not displayed on the device

8.104 set_height_m_0

Operator

This field is not displayed on the device, Operator: __{SET(538870,__{DIV(**height_0 (8.97)** ,100)}__)}__

8.105 height_2_0

Numeric

This field is not displayed on the device

8.106 set_height_2_0

Operator

This field is not displayed on the device, Operator: __{SET(538874,__{MUL(**height_m_0 (8.103)** , **height_m_0 (8.103)**)}__)}__

8.107 bmi_ur_0

Numeric

This field is not displayed on the device

8.108 set_bmi_ur_0

Operator

This field is not displayed on the device, Operator: __{SET(538878,__{DIV(**weight_0 (8.100)** , **height_2_0 (8.105)**)}__)}__

8.109 bmi_set_0

Operator

This field is not displayed on the device, Operator: __{SET(528069,__{ROUND(**bmi_ur_0 (8.107)** ,2)}__)}__

8.110 waist_0

[Enter waist in cm (no decimal place)]

Expects a numeric response (**required**)

Prerequisites
Skip when *waist_0 (8.110)* Less Than '136'

8.111 waist_high_0

[You have entered an unusually high number. Please confirm or correct it.]

Expects a single option response (**required**)

☐ Confirmed [0]

☐ Correct it [1]

Branches

If response **Equals 'Correct it [1]'** then skip to ***waist_0 (8.110)***

Prerequisites
Skip when *waist_0 (8.110)* Greater Than '64'

8.112 waist_low_confirm_0

[You have entered an unusually low number: please confirm or correct it.]

Expects a single option response (**required**)

☐ Confirmed [0]

☐ Correct it [1]

Branches

If response **Equals 'Correct it [1]'** then skip to ***waist_0 (8.110)***

Prerequisites
Skip when *rx_hiv_0 (5.20)* Equals 'No [0]'

8.113 stigma_instr_0

We would like to understand how having HIV makes you feel. Please answer whether you agree or disagree with the following statements.

Prerequisites
Skip when *rx_hiv_0 (5.20)* Equals 'No [0]'

8.114 stigma1_0

It is difficult to tell people about my HIV infection

Expects a single option response (**required**)

☐ Agree [1]

☐ Disagree [0]

Prerequisites
Skip when *rx_hiv_0 (5.20)* Equals 'No [0]'

8.115 stigma2_0

Being HIV positive makes me feel dirty

Expects a single option response (**required**)

☐ Agree [1]

☐ Disagree [0]

Prerequisites
Skip when *rx_hiv_0 (5.20)* Equals 'No [0]'

8.116 stigma3_0

I feel guilty that I am HIV positive

Expects a single option response (**required**)

☐ Agree [1]

☐ Disagree [0]

Prerequisites
Skip when *rx_hiv_0 (5.20)* **Equals 'No [0]'**

8.117 stigma4_0

I am ashamed that I am HIV positive

Expects a single option response (**required**)

- ☐ Agree [1]
- ☐ Disagree [0]

Prerequisites
Skip when *rx_hiv_0 (5.20)* **Equals 'No [0]'**

8.118 stigma5_0

I sometimes feel worthless because I am HIV positive

Expects a single option response (**required**)

- ☐ Agree [1]
- ☐ Disagree [0]

Prerequisites
Skip when *rx_hiv_0 (5.20)* **Equals 'No [0]'**

8.119 stigma6_0

I hide my HIV status from others

Expects a single option response (**required**)

- ☐ Agree [1]
- ☐ Disagree [0]

8.120 gen_instr_0

We would now like to ask some general questions

8.121 race_0

How would you describe your ethnicity?

Expects a single option response (**required**)

- ☐ Black [0]
- ☐ White [1]
- ☐ Coloured [2]
- ☐ Indian [3]
- ☐ Other [4]
- ☐ Prefer not to say [6]

8.122 mstatus_0

What is your marital status?

Expects a single option response (**required**)

- ☐ Single [0]
- ☐ Married or living with a partner [1]
- ☐ Divorced [2]
- ☐ Separated [3]
- ☐ Widowed [4]

8.123 edu_0

What is the highest level of education you have completed?

Expects a single option response (**required**)

☐ Never went to school [0]

☐ Grade 1-7 (primary school) [1]

☐ Grade 8-12 (high school) [2]

☐ Tertiary/diploma [3]

8.124 clinic_instr_0

We would like to know how much money your health costs you. In order to do this we need to ask some questions about your use of health care services, your employment status and income. We would like to remind you that all the information you give us is confidential.

8.125 clinic_trans_0

How do you usually travel to this clinic (choose one of the following options)?

Expects a single option response (**required**)

☐ Walk [0]

☐ Taxi [1]

☐ Private motor vehicle (such as car) [2]

☐ Bus [3]

☐ Patient transport/ambulance [4]

☐ Bicycle [5]

☐ Other [6]

Prerequisites
Skip when ***clinic_trans_0 (8.125)*** **Not Equal 'Other [6]'**

8.126 clinic_trans_other_0

Please specify:

Expects a long text response (**required**)

Prerequisites
Skip when ***clinic_trans_0 (8.125)*** **Equals 'Walk [0]'** OR
Skip when ***clinic_trans_0 (8.125)*** **Equals 'Taxi [1]'** OR
Skip when ***clinic_trans_0 (8.125)*** **Equals 'Bus [3]'** OR
Skip when ***clinic_trans_0 (8.125)*** **Equals 'Patient transport/ambulance [4]'** OR
Skip when ***clinic_trans_0 (8.125)*** **Equals 'Bicycle [5]'**

8.127 clinic_dist_0

What was the distance travelled to the clinic (in km one way)?

Expects a numeric response (**required**)

Prerequisites
Skip when *clinic_dist_0 (8.127)* **Less Than '51'** OR
Skip when *clinic_trans_0 (8.125)* **Equals 'Walk [0]'** OR
Skip when *clinic_trans_0 (8.125)* **Equals 'Taxi [1]'** OR
Skip when *clinic_trans_0 (8.125)* **Equals 'Bus [3]'** OR
Skip when *clinic_trans_0 (8.125)* **Equals 'Patient transport/ambulance [4]'** OR
Skip when *clinic_trans_0 (8.125)* **Equals 'Bicycle [5]'**

8.128 clinic_dist_high_0

[You have entered an unusually high number. Please confirm or correct it.]

Expects a single option response (**required**)

☐ Confirmed [0]

☐ Correct it [1]

Branches

If response **Equals 'Correct it [1]'** then skip to *clinic_dist_0 (8.127)*

Prerequisites
Skip when *clinic_trans_0 (8.125)* **Equals 'Walk [0]'** OR
Skip when *clinic_trans_0 (8.125)* **Equals 'Private motor vehicle (such as car) [2]'** OR
Skip when *clinic_trans_0 (8.125)* **Equals 'Patient transport/ambulance [4]'** OR
Skip when *clinic_trans_0 (8.125)* **Equals 'Bicycle [5]'**

8.129 clinic_fare_0

Do you usually pay a transport fare?

Expects a single option response (**required**)

☐ No [0]

☐ Yes [1]

Prerequisites
Skip when *clinic_fare_0 (8.129)* **Equals 'No [0]'** OR
Skip when *clinic_trans_0 (8.125)* **Equals 'Walk [0]'** OR
Skip when *clinic_trans_0 (8.125)* **Equals 'Private motor vehicle (such as car) [2]'** OR
Skip when *clinic_trans_0 (8.125)* **Equals 'Patient transport/ambulance [4]'** OR
Skip when *clinic_trans_0 (8.125)* **Equals 'Bicycle [5]'**

8.130 clinic_fare_amount_0

How much do you pay for a return fare?

Expects a decimal response (**required**)

Prerequisites
Skip when *clinic_fare_0 (8.129)* **Equals 'No [0]'** OR
Skip when *clinic_trans_0 (8.125)* **Equals 'Walk [0]'** OR
Skip when *clinic_trans_0 (8.125)* **Equals 'Private motor vehicle (such as car) [2]'** OR
Skip when *clinic_trans_0 (8.125)* **Equals 'Patient transport/ambulance [4]'** OR
Skip when *clinic_trans_0 (8.125)* **Equals 'Bicycle [5]'** OR
Skip when *clinic_fare_amount_0 (8.130)* **Less Than '51'**

8.131 clinic_fare_high_0

[You have entered an unusually high number. Please confirm or correct it.]

Expects a single option response (**required**)

☐ Confirmed [0]

☐ Correct it [1]

Branches

If response **Equals 'Correct it [1]'** then skip to *clinic_fare_amount_0 (8.130)*

8.132 clinic_visits_0

Aside from today, have you visited this clinic in the last 3 months for your own health care?

Expects a single option response (**required**)

☐ No [0]

☐ Yes [1]

Prerequisites
Skip when *clinic_visits_0 (8.132)* **Equals 'No [0]'**

8.133 clinic_visits_num_0

How many times have you visited this clinic in the last 3 months (excluding today) for your own health care?

Expects a numeric response (**required**)

Prerequisites
Skip when *clinic_visits_0 (8.132)* **Equals 'No [0]'** OR
Skip when *clinic_visits_num_0 (8.133)* **Less Than '13'**

8.134 clinic_visits_num_high_0

[You have entered an unusually high number. Please confirm or correct it.]

Expects a single option response (**required**)

- ☐ Confirmed [0]
- ☐ Correct it [1]

Branches
If response **Equals 'Correct it [1]'** then skip to *clinic_visits_num_0 (8.133)*

8.135 hcp_0

Have you visited any other health care provider in the last 3 months for your own health care?

Expects a single option response (**required**)

- ☐ No [0]
- ☐ Yes [1]

Branches
If response **Equals 'No [0]'** then skip to *hosp_3mnt_0 (11.1)*

8.136 hcp_list_0

Please indicate which of the following health care providers you have visited in the last 3 months for your own health care. I will also ask you some more details about those visits.

Expects multiple selected options (**required**)

- ☐ Another clinic [0]
- ☐ Hospital (outpatient visits only) [1]
- ☐ General Practitioner [2]
- ☐ Private Pharmacy [3]
- ☐ Traditional healer/ herbalist [4]
- ☐ Other [5]

Prerequisites
Skip when *hcp_list_0 (8.136)* **Excludes 'Other [5]'**

8.137 hcp_other_0

Please state which other health care provider you visited in the last 3 months for your own health care

Expects a long text response (**required**)

Section 9. Health Care Setup

9.1 hcp_count_0

Numeric

This field is not displayed on the device

9.2 hcp_count_setter_0

Operator

This field is not displayed on the device, Operator: __{SET(514041, Count(**hcp_list_0 (8.136)**))}__

9.3 hcp_count_reprocess_0

Operator

This field is not displayed on the device, Operator: Reprocess(**hcp_count_0 (9.1)**)

Prerequisites
Skip when ***hcp_count_0 (9.1)*** **Greater Than '-1'**

9.4 hcp_list_loop_0

This is a helper question for the repeat and is never shown

Expects a single option response (optional)

9.5 hcp_list_loop_clear_0

Operator

This field is not displayed on the device, Operator: REMOVEOPTION(,524021)

Prerequisites
Skip when ***hcp_list_0 (8.136)*** **Excludes 'Another clinic [0]'**

9.6 hcp_list_loop_addclinic_0

Operator

This field is not displayed on the device, Operator: ADDOPTION(ANOTHER CLINIC,ANOTHER CLINIC,524021)

Prerequisites
Skip when ***hcp_list_0 (8.136)*** **Excludes 'Hospital (outpatient visits only) [1]'**

9.7 hcp_list_loop_addhospital_0

Operator

This field is not displayed on the device, Operator: ADDOPTION(HOSPITAL,HOSPITAL,524021)

Prerequisites
Skip when ***hcp_list_0 (8.136)*** **Excludes 'General Practitioner [2]'**

9.8 hcp_list_loop_addgp_0

Operator

This field is not displayed on the device, Operator: ADDOPTION(GENERAL PRACTITIONER,GENERAL PRACTITIONER,524021)

Prerequisites
Skip when ***hcp_list_0 (8.136)*** **Excludes 'Private Pharmacy [3]'**

9.9 hcp_list_loop_addpharmacy_0

Operator

This field is not displayed on the device, Operator: ADDOPTION(PRIVATE PHARMACY,PRIVATE PHARMACY,524021)

Prerequisites
Skip when ***hcp_list_0 (8.136)*** **Excludes 'Traditional healer/ herbalist [4]'**

9.10 hcp_list_loop_addhealer_0

Operator

This field is not displayed on the device, Operator: ADDOPTION(TRADITIONAL HEALER,TRADITIONAL HEALER,524021)

Prerequisites
Skip when ***hcp_list_0 (8.136)*** **Excludes 'Other [5]'**

9.11 hcp_list_loop_addother_0

Operator

This field is not displayed on the device, Operator: __{ADDOPTION(**hcp_other_0 (8.137)** , **hcp_other_0 (8.137)** ,524021)}__

Repeat this section for value of *hcp_count_0 (9.1)*

Section 10. Health Care Provider

10.1 hcp_name_0

Text

This field is not displayed on the device, Value: Unknown

10.2 hcp_name_set_0

Operator

This field is not displayed on the device, Operator: __{SET(524916, OptionValue(**hcp_list_loop_0 (9.4)** ,REPEAT IDX))}__

10.3 hcp_instruction_0

The following questions are about your visits to **hcp_name_0 (10.1)**

10.4 hcp_num_0

Number of visits in the last 3 months

Expects a numeric response (**required**)

Prerequisites
Skip when *hcp_num_0 (10.4)* **Less Than '13'**

10.5 hcp_num_high_0

[You have entered an unusually high number. Please confirm or correct it.]

Expects a single option response (**required**)

☐ Confirmed [0]

☐ Correct it [1]

Branches

If response **Equals 'Correct it [1]'** then skip to *hcp_num_0 (10.4)*

10.6 hcp_fee_0

Did you pay a fee on your last visit?

Expects a single option response (**required**)

☐ No [0]

☐ Yes [1]

Prerequisites
Skip when *hcp_fee_0 (10.6)* **Equals 'No [0]'**

10.7 hcp_feeamt_0

What was the fee in rands?

Expects a decimal response (**required**)

Prerequisites
Skip when *hcp_feeamt_0 (10.7)* **Less Than '151'** OR
Skip when *hcp_fee_0 (10.6)* **Equals 'No [0]'**

10.8 hcp_feeamt_high_0

[You have entered an unusually high number. Please confirm or correct it.]

Expects a single option response (**required**)

- ☐ Confirmed [0]
- ☐ Correct it [1]

Branches

If response **Equals 'Correct it [1]'** then skip to *hcp_feeamt_0 (10.7)*

10.9 hcp_tra_0

What transport did you use to travel to your last visit to this provider?

Expects a single option response (**required**)

- ☐ Walk [0]
- ☐ Taxi [1]
- ☐ Private motor vehicle (such as car) [2]
- ☐ Bus [3]
- ☐ Patient transport/ambulance [4]
- ☐ Other [6]

Prerequisites
Skip when *hcp_tra_0 (10.9)* **Not Equal 'Other [6]'**

10.10 hcp_oth_tra_spec_0

Please specify

Expects a long text response (**required**)

Prerequisites
Skip when *hcp_tra_0 (10.9)* **Equals 'Walk [0]'** OR
Skip when *hcp_tra_0 (10.9)* **Equals 'Taxi [1]'** OR
Skip when *hcp_tra_0 (10.9)* **Equals 'Bus [3]'** OR
Skip when *hcp_tra_0 (10.9)* **Equals 'Patient transport/ambulance [4]'**

10.11 hcp_dis_0

What was the distance travelled to the clinic (in km one way)?

Expects a numeric response (**required**)

Prerequisites
Skip when *hcp_dis_0 (10.11)* **Less Than '51'** OR
Skip when *hcp_tra_0 (10.9)* **Equals 'Walk [0]'** OR
Skip when *hcp_tra_0 (10.9)* **Equals 'Taxi [1]'** OR
Skip when *hcp_tra_0 (10.9)* **Equals 'Bus [3]'** OR
Skip when *hcp_tra_0 (10.9)* **Equals 'Patient transport/ambulance [4]'**

10.12 hcp_travamt_high_0

[You have entered an unusually high number. Please confirm or correct it.]

Expects a single option response (**required**)

- ☐ Confirmed [0]
- ☐ Correct it [1]

Branches

If response **Equals 'Correct it [1]'** then skip to *hcp_dis_0 (10.11)*

Prerequisites
Skip when *hcp_tra_0 (10.9)* Equals 'Walk [0]' OR
Skip when *hcp_tra_0 (10.9)* Equals 'Private motor vehicle (such as car) [2]' OR
Skip when *hcp_tra_0 (10.9)* Equals 'Patient transport/ambulance [4]'

10.13 hcp_fare_0

Did you pay a transport fare on your last visit?

Expects a single option response (**required**)

☐ No [0]

☐ Yes [1]

Prerequisites
Skip when *hcp_tra_0 (10.9)* Equals 'Walk [0]' OR
Skip when *hcp_tra_0 (10.9)* Equals 'Private motor vehicle (such as car) [2]' OR
Skip when *hcp_tra_0 (10.9)* Equals 'Patient transport/ambulance [4]' OR
Skip when *hcp_fare_0 (10.13)* Equals 'No [0]'

10.14 hcp__fareamt_0

What was the return fare in rands?

Expects a decimal response (**required**)

Prerequisites
Skip when *hcp__fareamt_0 (10.14)* Less Than '51' OR
Skip when *hcp_tra_0 (10.9)* Equals 'Walk [0]' OR
Skip when *hcp_tra_0 (10.9)* Equals 'Private motor vehicle (such as car) [2]' OR
Skip when *hcp_tra_0 (10.9)* Equals 'Patient transport/ambulance [4]'

10.15 hcp_feeamt_high2_0

[You have entered an unusually high number. Please confirm or correct it.]

Expects a single option response (**required**)

☐ Confirmed [0]

☐ Correct it [1]

Branches

If response **Equals 'Correct it [1]'** then skip to *hcp__fareamt_0 (10.14)*

Section 11. Hospital

11.1 hosp_3mnt_0

Have you been admitted to hospital in the last 3 months?

Expects a single option response (**required**)

☐ No [0]

☐ Yes [1]

Branches

If response **Equals 'No [0]'** then skip to ***job_0 (13.1)***

Prerequisites
Skip when ***hosp_3mnt_0 (11.1)*** **Equals 'No [0]'**

11.2 hosp_num_0

How many times have you been admitted to hospital in the last 3 months?

Expects a numeric response (**required**)

Prerequisites
Skip when ***hosp_num_0 (11.2)*** **Less Than '13'**

11.3 hosp_num_high_0

[You have entered an unusually high number. Please confirm or correct it.]

Expects a single option response (**required**)

☐ Confirmed [0]

☐ Correct it [1]

Branches

If response **Equals 'Correct it [1]'** then skip to ***hosp_num_0 (11.2)***

Prerequisites
Skip when ***hosp_num_0 (11.2)*** **Equals '0'**

11.4 admission_instr_0

I would like to ask you a few questions about each of your admissions in the last 3 months:

Repeat this section for value of *hosp_num_0 (11.2)*

Section 12. Admission

12.1 hosp_date_0

Admission REPEAT IDX: when were you admitted?

Expects a date response (optional)

Constraints

Response must be ***Less Than 'DATENOW'***

12.2 set_hosp_date_check_0

Operator

This field is not displayed on the device, Operator: __{SET(525620,__{DAYSBETWEEN(**hosp_date_0 (12.1)** , DATENOW)}__)}__

12.3 hosp_date_check_0

Numeric

This field is not displayed on the device, Value: 0

Prerequisites
Skip when *hosp_date_check_0 (12.3)* **Less Than '93'**

12.4 hosp_date_check_fb_0

[The date entered is more than 3 months ago. Please correct it.]

Expects a single option response (**required**)

☐ Correct it [0]

Branches

If response **Equals 'Correct it [0]'** then skip to *hosp_date_0 (12.1)*

12.5 hosp_nights_0

Admission REPEAT IDX: how many nights did you spend in hospital?

Expects a numeric response (optional)

Prerequisites
Skip when *hosp_nights_0 (12.5)* **Less Than '31'**

12.6 hosp_nights_high_0

[You have entered an unusually high number. Please confirm or correct it.]

Expects a single option response (**required**)

☐ Confirmed [0]

☐ Correct it [1]

Branches

If response **Equals 'Correct it [1]'** then skip to *hosp_nights_0 (12.5)*

12.7 hosp_name_0

Admission REPEAT IDX: What was the name of the hospital?

Expects a single option response (optional)

☐ Klerksdorp (Tshepong A) [Klerksdorp (Tshepong A)]

☐ Potchefstroom [Potchefstroom]

☐ Nic Bodenstein [Nic Bodenstein]

☐ Ventersdorp [Ventersdorp]

☐ Witrand [Witrand]

☐ Other (please specify) [Other (please specify)]

Prerequisites
Skip when *hosp_name_0 (12.7)* Not Equal 'Other (please specify) [Other (please specify)]'

12.8 hosp_name_other_0

Admission REPEAT IDX: please specify the hospital name

Expects a single line text response (**required**)

12.9 hosp_exp_0

Admission REPEAT IDX: What was the total amount you had to pay for your admission (your out-of-pocket expenses)?

Expects a decimal response (**required**)

Prerequisites
Skip when *hosp_exp_0 (12.9)* Less Than '151'

12.10 hosp_exp_high_0

[You have entered an unusually high number. Please confirm or correct it.]

Expects a single option response (**required**)

☐ Confirmed [0]

☐ Correct it [1]

Branches

If response **Equals 'Correct it [1]'** then skip to *hosp_exp_0 (12.9)*

12.11 hosp_trans_0

Admission REPEAT IDX: How did you travel to the hospital (choose one of the following options):

Expects a single option response (**required**)

☐ Walk [0]

☐ Taxi [1]

☐ Private motor vehicle (such as car) [2]

☐ Bus [3]

☐ Patient transport/ambulance [4]

☐ Bicycle [5]

☐ Other [6]

Prerequisites
Skip when *hosp_trans_0 (12.11)* **Not Equal 'Other [6]'**

12.12 hosp_trans_other_0

Admission REPEAT IDX: please specify

Expects a long text response (**required**)

Prerequisites
Skip when *hosp_trans_0 (12.11)* **Equals 'Walk [0]'** OR
Skip when *hosp_trans_0 (12.11)* **Equals 'Taxi [1]'** OR
Skip when *hosp_trans_0 (12.11)* **Equals 'Bus [3]'** OR
Skip when *hosp_trans_0 (12.11)* **Equals 'Patient transport/ambulance [4]'** OR
Skip when *hosp_trans_0 (12.11)* **Equals 'Bicycle [5]'**

12.13 hosp_dist_0

Admission REPEAT IDX: what was the distance travelled to the hospital (in km one way) ?

Expects a numeric response (**required**)

Prerequisites
Skip when *hosp_dist_0 (12.13)* **Less Than '51'** OR
Skip when *hosp_trans_0 (12.11)* **Not Equal 'Private motor vehicle (such as car) [2]'** OR
Skip when *hosp_trans_0 (12.11)* **Not Equal 'Other [6]'**

12.14 hosp_dist_high_0

[You have entered an unusually high number. Please confirm or correct it.]

Expects a single option response (**required**)

☐ Confirmed [0]

☐ Correct it [1]

Branches
If response **Equals 'Correct it [1]'** then skip to *hosp_dist_0 (12.13)*

12.15 hosp_fare_0

Admission REPEAT IDX: Did you pay a transport fare to the hospital?

Expects a single option response (**required**)

☐ No [0]

☐ Yes [1]

Prerequisites
Skip when *hosp_fare_0 (12.15)* **Equals 'No [0]'**

12.16 hosp_fare_amount_0

How much did you pay for a return fare?

Expects a decimal response (**required**)

Prerequisites
Skip when ***hosp_fare_amount_0 (12.16)*** **Less Than '51'** OR
Skip when ***hosp_fare_0 (12.15)*** **Equals 'No [0]'**

12.17 hosp_fare_amt_high_0

[You have entered an unusually high number. Please confirm or correct it.]

Expects a single option response (**required**)

- ☐ Confirmed [0]
- ☐ Correct it [1]

Branches

If response **Equals 'Correct it [1]'** then skip to ***hosp_fare_amount_0 (12.16)***

Section 13. Income

13.1 job_0

We would now like to ask about your employment and income. Please remember that all information you provide is confidential: Which of the following best describes your employment status? Choose one:

Expects a single option response (**required**)

- ☐ Employed [0]
- ☐ Self-employed [1]
- ☐ Student/learner [2]
- ☐ Unemployed and looking for work [3]
- ☐ Unemployed and not looking for work [4]

Prerequisites
Skip when ***job_0 (13.1)*** **Equals 'Employed [0]'** OR
Skip when ***job_0 (13.1)*** **Equals 'Self-employed [1]'** OR
Skip when ***job_0 (13.1)*** **Equals 'Student/learner [2]'** OR
Skip when ***job_0 (13.1)*** **Equals 'Unemployed and looking for work [3]'**

13.2 job_notlooking_0

Are you not looking for work but able to work, unable to work, or retired?

Expects a single option response (**required**)

- ☐ Not looking for work but able to work [0]
- ☐ Not looking for work and unable to work [1]
- ☐ Retired [2]

Prerequisites
Skip when ***job_0 (13.1)*** **Equals 'Student/learner [2]'** OR
Skip when ***job_0 (13.1)*** **Equals 'Unemployed and looking for work [3]'** OR
Skip when ***job_0 (13.1)*** **Equals 'Unemployed and not looking for work [4]'**

13.3 job_income_0

How much did you earn last month (excluding grant income), before tax and any other deductions?

Expects a decimal response (**required**)

Prerequisites
Skip when ***job_0 (13.1)*** **Equals 'Student/learner [2]'** OR
Skip when ***job_0 (13.1)*** **Equals 'Unemployed and looking for work [3]'** OR
Skip when ***job_0 (13.1)*** **Equals 'Unemployed and not looking for work [4]'**

13.4 job_days_lost_0

How many days were you unable to work because of illness in the last 3 months (including health care visits)?

Expects a numeric response (**required**)

Constraints

*Response must be **Less Than or Equal '93'***

Prerequisites
Skip when ***job_0 (13.1)*** **Not Equal 'Student/learner [2]'**

13.5 job_stud_days_lost_0

How many days have you been unable to attend school/college because of illness in the last 3 months (including health care visits)?

Expects a numeric response (**required**)

Constraints

*Response must be **Less Than or Equal '93'***

13.6 grant_0

Are you getting a pension or grant?

Expects a single option response (**required**)

- ☐ No [0]
- ☐ Yes [1]

Prerequisites
Skip when *grant_0 (13.6)* Equals 'No [0]'

13.7 grant_list_0

Please indicate which grants you currently get (tick all that apply)

Expects multiple selected options (**required**)

- ☐ Older person's grant (if over 60 years) [0]
- ☐ Disability grant (if unfit to work) [1]
- ☐ War veteran's grant [2]
- ☐ Grant-in-aid (if in need of full time care [3]
- ☐ Child support grant (if child younger than 18 years) [4]
- ☐ Foster care grant [5]
- ☐ Care dependence grant (if child has a disability) [6]
- ☐ Social relief of distress award [7]
- ☐ Other [8]

Prerequisites
Skip when *grant_list_0 (13.7)* Excludes 'Other [8]'

13.8 grant_other_0

Please specify which other grant you are receiving.

Expects a long text response (**required**)

Prerequisites
Skip when *grant_0 (13.6)* Equals 'No [0]'

13.9 grant_income_0

What was your total grant income in the last month?

Expects a decimal response (**required**)

13.10 job_lost_0

Have you lost your job or resigned because of illness during the past year?

Expects a single option response (**required**)

- ☐ No [0]
- ☐ Yes [1]

Branches

If response **Equals 'No [0]'** then skip to *ass_instr_0 (13.13)*

13.11 job_lost_income_0

Before you lost your job or resigned, how much did you earn in the last month you worked (excluding grant income), before tax and any other deductions?

Expects a decimal response (**required**)

13.12 job_lost_new_0

Since losing your job or resigning, have you got another job?

Expects a single option response (**required**)

- ☐ No [0]
- ☐ Yes [1]

13.13 ass_instr_0

We would now like to ask about the characteristics of your home:

13.14 ass_rad_0

Does your household have a television or radio?

Expects a single option response (**required**)

- ☐ No [0]
- ☐ Yes [1]

13.15 ass_hot_0

Does your household have a 2-plate hotplate?

Expects a single option response (**required**)

- ☐ No [0]
- ☐ Yes [1]

13.16 asset_fri_0

Does your household have a refrigerator?

Expects a single option response (**required**)

- ☐ No [0]
- ☐ Yes [1]

13.17 ass_mic_0

Does your household have a microwave?

Expects a single option response (**required**)

- ☐ No [0]
- ☐ Yes [1]

13.18 ass_sto_0

Does your household have a stove (hotplate with oven)?

Expects a single option response (**required**)

- ☐ No [0]
- ☐ Yes [1]

13.19 ass_dst_0

Does your household have DSTV?

Expects a single option response (**required**)

- ☐ No [0]
- ☐ Yes [1]

13.20 ass_fan_0

Does your household have a fan?

Expects a single option response (**required**)

- ☐ No [0]
- ☐ Yes [1]

13.21 ass_was_0

Does your household have a washing machine?

Expects a single option response (**required**)

- ☐ No [0]
- ☐ Yes [1]

13.22 ass_hea_0

Does your household have a heater?

Expects a single option response (**required**)

- ☐ No [0]
- ☐ Yes [1]

13.23 ass_wat_0

What is the main source of drinking water for members of your household? Please select one option:

Expects a single option response (**required**)

- ☐ Outside shared (communal) tap [0]
- ☐ Tap inside home [1]
- ☐ Outside home, own tap [2]
- ☐ Outside other source of water [3]

13.24 ass_toi_0

What kind of toilet facility do members of your household usually use?

Expects a single option response (**required**)

- ☐ Inside own flush toilet [0]
- ☐ Outside shared flush toilet [1]
- ☐ Outside own flush toilet [2]
- ☐ Outside other toilet (not flush) [3]

13.25 ass_roo_0

How many rooms does your household have?

Expects a numeric response (**required**)

13.26 ass_typ_0

What type of home do you live in?

Expects a single option response (**required**)

- ☐ Shack [0]
- ☐ Brick/cement block home [1]
- ☐ Flat [2]
- ☐ Outbuilding [3]

13.27 occupants_0

How many people are living with you in your house (include yourself)?

Expects a numeric response (**required**)

Prerequisites
Skip when *occupants_0 (13.27)* Less Than '13'

13.28 occupants_high_0

[You have entered an unusually high number. Please confirm or correct it.]

Expects a single option response (**required**)

- ☐ Confirmed [0]
- ☐ Correct it [1]

Branches

If response **Equals 'Correct it [1]'** then skip to *occupants_0 (13.27)*

13.29 pacic_instr_0

Staying healthy can be difficult when you have a chronic condition. We would like to learn about the type of help you get from health workers.

13.30 pacic1_0

Over the past 3 months, when you received care at the clinic, how often were you: Satisfied that your care was well organized, for example, that there was good coordination between different providers involved with your care?

Expects a single option response (**required**)

- ☐ None of the time [1]
- ☐ A little of the time [2]
- ☐ Some of the time [3]
- ☐ Most of the time [4]
- ☐ Always [5]

13.31 pacic2_0

Over the past 3 months, when you received care at the clinic, how often were you encouraged by providers to take more care of your condition, for example, shown how any actions that you have taken to take care of yourself has improved your condition?

Expects a single option response (**required**)

- ☐ None of the time [1]
- ☐ A little of the time [2]
- ☐ Some of the time [3]
- ☐ Most of the time [4]
- ☐ Always [5]

13.32 pacic3_0

Over the past 3 months, when you received care at the clinic, how often were you: Helped to set specific goals to improve your condition, e.g., getting active, acting on a problem, joining a support group?

Expects a single option response (**required**)

- ☐ None of the time [1]
- ☐ A little of the time [2]
- ☐ Some of the time [3]
- ☐ Most of the time [4]
- ☐ Always [5]

13.33 pacic4_0

Over the past 3 months, when you received care at the clinic, how often were you: Asked about your understanding of your problem and whether the treatment/interventions recommended were in alignment with your values, beliefs and traditions?

Expects a single option response (**required**)

- ☐ None of the time [1]
- ☐ A little of the time [2]
- ☐ Some of the time [3]
- ☐ Most of the time [4]
- ☐ Always [5]

13.34 pacic5_0

Over the past 3 months, when you received care at the clinic, how often did you: Obtain skills to help you to deal with your condition better even when under great stress or hard times?

Expects a single option response (**required**)

- ☐ None of the time [1]
- ☐ A little of the time [2]
- ☐ Some of the time [3]
- ☐ Most of the time [4]
- ☐ Always [5]

13.35 pacic6_0

Over the past 3 months, when you received care at the clinic, how often were you: Encouraged to attend programs in the community that could help you?

Expects a single option response (**required**)

- ☐ None of the time [1]
- ☐ A little of the time [2]
- ☐ Some of the time [3]
- ☐ Most of the time [4]
- ☐ Always [5]

13.36 **pacic7_0**

Over the past 3 months, when you received care at the clinic, how often were you: Referred to a counsellor or equivalent who could help you with your psychosocial problems?

Expects a single option response (**required**)

- ☐ None of the time [1]
- ☐ A little of the time [2]
- ☐ Some of the time [3]
- ☐ Most of the time [4]
- ☐ Always [5]

13.37 **pacic8_0**

Over the past 3 months, when you received care at the clinic, how often were you: Asked how your visits with other providers were going?

Expects a single option response (**required**)

- ☐ None of the time [1]
- ☐ A little of the time [2]
- ☐ Some of the time [3]
- ☐ Most of the time [4]
- ☐ Always [5]

13.38 **pacic9_0**

Over the past 3 months, when you received care at the clinic, how often were you given: Information and education about your condition

Expects a single option response (**required**)

- ☐ None of the time [1]
- ☐ A little of the time [2]
- ☐ Some of the time [3]
- ☐ Most of the time [4]
- ☐ Always [5]

13.39 **pacic10_0**

Over the past 3 months, when you received care at the clinic, how often did you receive: Follow up appointments for your condition

Expects a single option response (**required**)

- ☐ None of the time [1]
- ☐ A little of the time [2]
- ☐ Some of the time [3]
- ☐ Most of the time [4]
- ☐ Always [5]

13.40 **contact_instr_0**

It is important for our study that we interview you in 6 months time, and again in 12 months time. I will schedule those appointments and we will send you SMS reminders 2 weeks before the appointment. I will provide you with a contact number. Please let us know if your cell phone number changes or if you need to reschedule the appointment. In order to make it as easy as possible to contact you if necessary, I would be grateful if you could provide as many of the following contact details as possible:

13.41 address_home_0

Home address

Expects a long text response (optional)

13.42 phone_home_0

Telephone number at home

Expects a phone number (optional)

Constraints

Response must be ***Match '^[0-9]{10}$'***

13.43 phone_cell_confirm_set_0

Operator

This field is not displayed on the device, Operator: __{SET(514161, **phone_cell_0 (7.6)**)}__

13.44 phone_cell_confirm_0

Please confirm the following cell phone number as correct:

Expects a phone number (optional)

Constraints

Response must be ***Match '^[0-9]{10}$'***

13.45 phone_work_0

Work telephone number

Expects a phone number (optional)

Constraints

Response must be ***Match '^[0-9]{10}$'***

13.46 phone_other_0

Alternative number (friend, relative, neighbour)

Expects a phone number (optional)

Constraints

Response must be ***Match '^[0-9]{10}$'***

13.47 folder_0

Clinic folder number

Expects a single line text response (**required**)

13.48 is_sa_citizin_0

What type of identity document do you have?

Expects a single option response (**required**)

☐ South African [0]

☐ Other [1]

Prerequisites
Skip when *is_sa_citizin_0 (13.48)* **Equals 'Other [1]'**

13.49 sa_id_1_0

South African ID number

Expects a numeric response (optional)

Constraints

Response must be ***Match '__{VALID_RSA_ID}__'***

Prerequisites
Skip when *is_sa_citizin_0 (13.48)* **Equals 'Other [1]'**

13.50 sa_id_2_0

Re-enter ID number

Expects a numeric response (optional)

Constraints

Response must be ***Equals 'sa_id_1_0 (13.49)'***

Prerequisites
Skip when *is_sa_citizin_0 (13.48)* **Equals 'South African [0]'**

13.51 other_id_0

Enter the identity/passport number.

Expects a single line text response (optional)

13.52 goodbye_instr_0

We would like to thank you for your time today. We look forward to seeing you in 6 months.

Section 14. Schedule

14.1 followup_scheduled_dyna_0

Operator

This field is not displayed on the device, Operator: Set(**followup_scheduled_0 (14.2)** , DATEADD(DATENOW ,182))

14.2 followup_scheduled_0

We have reached the end of the interview and would like to schedule your 6m assessment. Your 6m ideal date is : DATEADD(DATENOW ,182) [Fieldworker: Please schedule an appointment with the participant as close to the ideal date as possible] NB!! Please fill in the appointment card and diary!

Expects a date response (**required**)

14.3 followup_days_diff_0

Numeric

This field is not displayed on the device, Value: 0

14.4 followup_days_setter_0

Operator

This field is not displayed on the device, Operator: __{SET(523979,__{ROUND(__{DAYSBETWEEN(DATEADD(DATENOW ,182) , **followup_scheduled_0 (14.2)**)}__,0)}__)}__

Prerequisites
Skip when ***followup_days_diff_0 (14.3)*** **Greater Than '-14'** AND
Skip when ***followup_days_diff_0 (14.3)*** **Less Than '14'**

14.5 followup_scheduled_warning_0

WARNING! The date you selected is more than 2 weeks outside the ideal date. Choose Correct It to change the appointment date, or select Proceed to confirm the appointment for **followup_scheduled_0 (14.2)**

Expects a single option response (**required**), Default: Correct it

- ☐ Correct it [0]
- ☐ Proceed [1]

Branches

If response **Equals 'Correct it [0]'** then skip to ***followup_scheduled_0 (14.2)***

14.6 followup12_scheduled_dyna_0

Operator

This field is not displayed on the device, Operator: Set(**followup12m_scheduled_0 (14.7)** , DATEADD(DATENOW ,365))

14.7 followup12m_scheduled_0

Please schedule your 12m assessment. Your 12m ideal date is : DATEADD(DATENOW ,365) [Fieldworker: Please schedule an appointment with the participant as close to the ideal date as possible]

Expects a date response (**required**)

14.8 followup12m_days_diff_0

Numeric

This field is not displayed on the device, Value: 0

14.9 followup12m_days_setter_0

Operator

This field is not displayed on the device, Operator: __{SET(525930,__{ROUND(__{DAYSBETWEEN(DATEADD(DATENOW ,365) , **followup12m_scheduled_0 (14.7)**)}__,0)}__)}__

Prerequisites
Skip when ***followup12m_days_diff_0 (14.8)*** **Greater Than '-14'** AND
Skip when ***followup12m_days_diff_0 (14.8)*** **Less Than '14'**

14.10 followup12m_scheduled_warning_0

WARNING! The date you selected is more than 2 weeks outside the ideal date. Choose Correct It to change the appointment date, or select Proceed to confirm the appointment for **followup12m_scheduled_0 (14.7)**

Expects a single option response (**required**), Default: Correct it

- ☐ Correct it [0]
- ☐ Proceed [1]

Branches

If response **Equals 'Correct it [0]'** then skip to ***followup12m_scheduled_0 (14.7)***

Section 15. End

15.1 comment_0

Text

This field is not displayed on the device

Prerequisites
Skip when *phq9b_0 (5.11)* Equals '1 - 7 days [1]' OR
Skip when *phq9b_0 (5.11)* Equals '0 days [0]'

15.2 phqscorefb_1_0

[Please read the following:] I am concerned about how you are feeling and would like to ask a nurse to see you at the end of this interview. [Please fill in the Mental Health referral letter' and lead the patient to the nurse at the end of the interview.]

Prerequisites
Skip when *phq9b_0 (5.11)* Equals '8 - 11 days [2]' OR
Skip when *phq9b_0 (5.11)* Equals '12 -14 days [3]' OR
Skip when *phq9b_0 (5.11)* Equals '0 days [0]'

15.3 phqscorefb_2_0

[Please read the following:] At the end of the interview I will give you a leaflet with some contact details for people who will be able to help with how you are feeling.

Prerequisites
Skip when *ref_bp_0 (1.9)* Equals '0'

15.4 high_bp_warn_0

I am concerned about your blood pressure which was very high. I would like to ask a nurse to see you today to assess it further. [Please fill in the 'high BP referral letter' and lead the patient to the nurse at the end of the interview.]

Prerequisites
Skip when *ref_vl_0 (1.8)* Equals '0'

15.5 vl_ref_letter

I would like to request that you have a viral load test done today as part of the study. [Please fill in the 'Viral load referral letter' and lead the patient to the nurse at the end of the interview.]

Prerequisites
Skip when *preconsent_0 (4.2)* Equals 'Yes [1]'

15.6 no_preconsent_0

You do not qualify for the study. However, we would like to thank you for your time today and we wish you well. [Select PROCEED to continue to the end of the survey.]

Expects a single option response (**required**)

☐ Proceed [1]

Branches

If response **Equals 'Proceed [1]'** then skip to ***end_0 (15.10)***

Prerequisites
Skip when *age_under18_0 (4.6)* **Not Equal 'Proceed [1]'**

15.7 age_less18_0

You do not qualify for the study, However, we would like to thank you for your time today and we wish you well. [Select PROCEED to continue to the end of the survey.]

Expects a single option response (**required**)

☐ Proceed [1]

Branches

If response **Equals 'Proceed [1]'** then skip to ***end_00 (15.11)***

Prerequisites
Skip when *area_0 (4.7)* **Equals 'Yes [1]'**

15.8 no_area_0

You do not qualify for the study. However, we would like to thank you for your time today and we wish you well. [Select PROCEED to continue to the end of the survey.]

Expects a single option response (**required**)

☐ Proceed [1]

Branches

If response **Equals 'Proceed [1]'** then skip to ***end_00 (15.11)***

Prerequisites
Skip when *area_0 (4.7)* **Equals 'No [0]'** OR
Skip when *preconsent_0 (4.2)* **Equals 'No [0]'** OR
Skip when *age_under18_0 (4.6)* **Equals 'Proceed [1]'**

15.9 voucherno_0

Please issue a voucher for the patient and record the voucher number:

Expects a single line text response (**required**)

Prerequisites
Skip when *area_0 (4.7)* **Equals 'No [0]'** OR
Skip when *preconsent_0 (4.2)* **Equals 'No [0]'** OR
Skip when *age_under18_0 (4.6)* **Equals 'Proceed [1]'**

15.10 end_0

You have reached the end of the baseline survey. Press BACK to update any responses. MENUACTION(CUSTOMWEB,PARTICIPANT OVERVIEW)

Prerequisites
Skip when *area_0 (4.7)* **Equals 'Yes [1]'** OR
Skip when *preconsent_0 (4.2)* **Equals 'Yes [1]'**

15.11 end_00

You have reached the end of the baseline survey. Press BACK to update any responses.

Prerequisites
Skip when *area_0 (4.7)* **Equals 'No [0]'** OR
Skip when *preconsent_0 (4.2)* **Equals 'No [0]'** OR
Skip when *age_under18_0 (4.6)* **Equals 'Proceed [1]'**

15.12 set_reached_end_0

Operator

This field is not displayed on the device, Operator: Set(**reached_end_0 (1.18)** ,1)

15.13 end_000

Press NEXT to submit.
